# Synthesis and Cyclooxygenase Inhibition of Sulfonamide-Substituted (Dihydro)Pyrrolo[3,2,1-*hi*]indoles and Their Potential Prodrugs

**DOI:** 10.3390/molecules24203807

**Published:** 2019-10-22

**Authors:** Markus Laube, Cemena Gassner, Torsten Kniess, Jens Pietzsch

**Affiliations:** 1 Department of Radiopharmaceutical and Chemical Biology, Institute of Radiopharmaceutical Cancer Research, Helmholtz-Zentrum Dresden-Rossendorf, Bautzner Landstrasse 400, 01328 Dresden, Germany; c.gassner@hzdr.de (C.G.); t.kniess@hzdr.de (T.K.); 2 Faculty of Chemistry and Food Chemistry, School of Science, Technische Universität Dresden, Mommsenstrasse 4, 01062 Dresden, Germany

**Keywords:** cancer, imaging, inflammation, lipophilicity, McMurry cyclization, structure-activity-relationship

## Abstract

Non-invasive imaging of cyclooxygenase-2 (COX-2) by radiolabeled ligands is attractive for the diagnosis of cancer, and novel highly affine leads with optimized pharmacokinetic profile are of great interest for future developments. Recent findings have shown that methylsulfonyl-substituted (dihydro)pyrrolo[3,2,1-*hi*]indoles represent highly potent and selective COX-2 inhibitors but possess unsuitable pharmacokinetic properties for radiotracer applications. Based on these results, we herein present the development and evaluation of a second series of sulfonamide-substituted (dihydro)pyrrolo[3,2,1-*hi*]indoles and their conversion into the respective more hydrophilic *N*-propionamide-substituted analogs. In comparison to the methylsulfonyl-substituted leads, COX inhibition potency and selectivity was retained in the sulfonamide-substituted compounds; however, the high lipophilicity might hinder their future use. The *N*-propionamide-substituted analogs showed a significantly decreased lipophilicity and, as expected, lower or no COX-inhibition potency. Hence, the *N*-(sulfonyl)propionamides can be regarded as potential prodrugs, which represents a potential approach for more sophisticated radiotracer developments.

## 1. Introduction

Cyclooxygenases (COX) convert arachidonic acid into prostaglandin H_2_, which is the rate limiting step in the synthesis of prostanoids. These are potent lipid mediators that control a variety of physiological and pathophysiological processes. COX exists in two isoforms—the constitutively expressed COX-1 and the inducible COX-2 isoform. While COX-1 is mainly responsible for the production of prostanoids to maintain homeostatic processes, e.g., in the gastric mucosa, COX-2 represents a key player in inflammation. It is nearly absent in most tissues under physiological conditions, but its expression is induced by inflammatory and proliferative stimuli to provide COX-2-derived prostanoids locally for the regulation of the inflammatory process. Overexpression of COX-2 is associated with acute and chronic inflammatory diseases, neurological disorders, and cancer [1,2,3,4,5,6]. In this regard, non-invasive imaging of COX-2 in vivo by means of radiolabeled probes for single photon emission computed tomography (SPECT) or positron emission tomography (PET), e.g., by ^18^F-labeled COX-2 inhibitors, for early diagnosis or therapy monitoring of cancer represents an unmet need [7,8,9,10,11].

**Figure 1:**
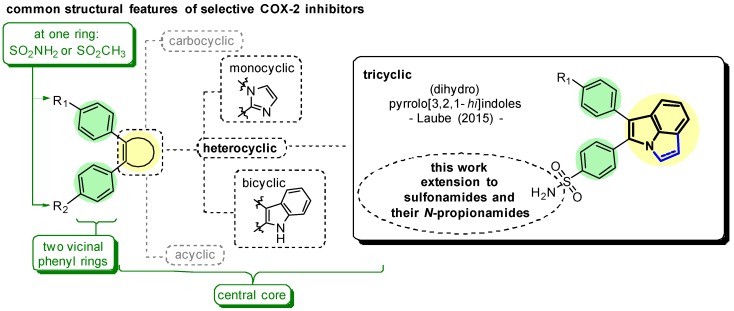
General structure of cyclooxygenase-2 (COX-2) inhibitors and target compounds of this work.

Inhibition of COX-2 has anti-inflammatory, antipyretic, and analgesic effects and can be achieved—based on the COX-1/COX-2-isoform selectivity—either by non-selective nonsteroidal anti-inflammatory drugs (NSAIDs) or selective COX-2 inhibitors (COXIBs). The most common structural characteristic of COXIBs is the methyl- or aminosulfonyl-substituent at one of two vicinal phenyl rings, which are linked to an acyclic, carbocyclic, or heterocyclic core (Figure 1) [2,12]. Of note, the indole heterocycle, a prominent pharmacophore in medicinal chemistry, was previously described as part of potent and selective COXIBs, some of them showing even fluorescent or antioxidant properties [3,13,14]. However, an ^18^F-labeled indole-containing radiotracer developed by us turned out to be unsuitable for targeting COX-2 in vivo [15].

Based on our ongoing interest to develop novel probes in this field, we found a set of methylsulfonyl-substituted tricyclic 1,2-dihydropyrrolo[3,2,1-*hi*]indoles and pyrrolo[3,2,1-*hi*]indoles that show highly potent and selective COX-2 inhibition and radiolabeled one derivative with fluorine-18 [16,17]. Unfortunately, in vitro cell uptake studies as well as in vivo studies using the COX-2-positive human melanoma cell line A2058 revealed that this radiotracer was unable to target COX-2 due to high unspecific binding in cells and off-target tissues caused by its high lipophilicity as well as due to fast hepatobiliary excretion. In this regard, we herein report the development of a complementary sulfonamide-substituted set of 1,2-dihydropyrrolo[3,2,1-*hi*]indole- and pyrrolo[3,2,1-*hi*]indole-based compounds, as well as their conversion to their more hydrophilic *N*-propionamide-substituted prodrugs (Figure 1), in order to identify leads with improved pharmacokinetics for the development of COX-2 targeting radiotracers.

## 2. Results

### 2.1. Synthesis

**Scheme 1:**
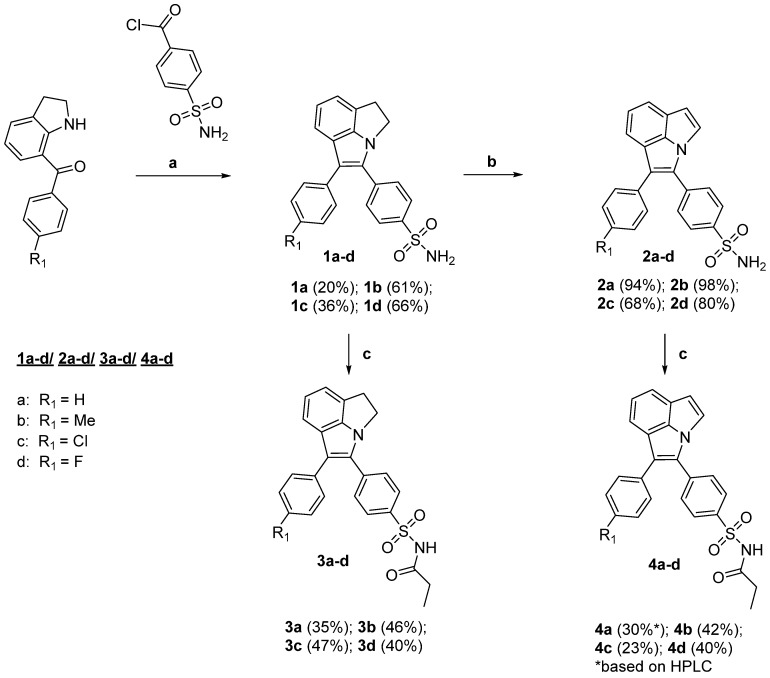
Synthesis sequence for (dihydro)pyrrolo[3,2,1-*hi*]indoles and their *N*-propionamides^a^ Reagents and conditions: (**a**) i: 4-(sulfamoyl)benzoic acid chloride, triethylamine, THF, r.t., ii: TiCl_4_, Zn, THF, 70 °C; (**b**) DDQ, benzene, 100 °C; (**c**) propionyl chloride, DMAP, DCM/THF, r.t. Yields are given in parentheses.

The 1,2-dihydropyrrolo[3,2,1-*hi*]indoles **1a**–**d** and pyrrolo[3,2,1-*hi*]indoles **2a**–**d** were synthesized with our previously developed regioselective synthetic route starting from indoline. In this procedure, well defined *N*,7-dibenzoyl-substituted indolines are prepared by BCl_3_-mediated Friedel-Crafts acylation followed by reaction with benzoyl chlorides. Afterwards, a McMurry based cyclization reaction forms in a regioselective manner the 4,5-diphenyl-substituted 1,2-dihydropyrrolo[3,2,1-*hi*]indoles **1a**–**d**. The oxidation with 2,3-dichloro-5,6-dicyano-1,4-benzoquinone (DDQ) allows the conversion to the 1,2-diphenyl-pyrrolo[3,2,1-*hi*]indoles **2a**–**d** (Scheme 1). In this work, we utilized the previously described unsubstituted as well as 4-methyl-, 4-chloro-, and 4-fluoro-substituted 7-benzoylindolines as starting material and converted them by an optimized two-step one-pot reaction directly to the respective 1,2-dihydropyrrolo[3,2,1-*hi*]indoles **1a**–**d**. For that, 4-sulfamoylbenzoic acid chloride was reacted with the 7-benzoylindolines and triethylamine in THF at room temperature to form the *N,*7-dibenzoyl-substituted indolines followed by direct addition of zinc and titanium tetrachloride and reaction at reflux to perform McMurry cyclization. By this route, the 1,2-dihydropyrrolo[3,2,1-*hi*]indoles **1a**–**d** were formed in 20–66% yield. The following dehydrogenation with DDQ in benzene formed the pyrrolo[3,2,1-*hi*]indoles **2a**–**d** in 68–98% yield. Both 1,2-dihydropyrrolo[3,2,1-*hi*]indoles **1a**–**d** and pyrrolo[3,2,1-*hi*]indoles **2a**–**d** were converted to the respective *N*-propionamides **3a**–**d** and **4a**–**d** (Scheme 1). Optimization experiments using propionyl chloride and triethylamine as base showed only low conversion to the respective *N*-propionamides. Instead, the formation of the bis-*N,N*-propionamides was favored, as indicated by mass spectrometry (see Supplementary Materials Figures S55 and S56). In contrast, the reaction of **1a**–**d** and **2a**–**d** with propionyl chloride and 4-dimethylaminopyridine (DMAP) as a base was found to be quantitative within 20 min at room temperature. This provided the *N*-propionamides **3a**–**d** and **4a**–**d** in microscale experiments in yields of 35–47%.

### 2.2. COX Inhibitory Activity and Lipophilicity

COX inhibition potency of the synthesized 1,2-dihydropyrrolo[3,2,1-*hi*]indoles **1a**–**d**, pyrrolo[3,2,1-*hi*]indoles **2a**–**d**, and their *N*-propionamide-substituted analogs **3a**–**d** and **4b**–**d** was determined in vitro using a commercial COX assay (“COX Fluorescent Inhibitor Screening Assay Kit,” Item No. 700100, Cayman Chemical, Ann Arbor, MI, USA). Celecoxib served as a reference. The results are shown in Table 1.

The sulfonamide-substituted 1,2-dihydropyrrolo[3,2,1-*hi*]indoles **1a**–**d** and pyrrolo[3,2,1-*hi*]indoles **2a**–**d** showed COX-2 selective inhibition, which is in accordance with the previous finding that the methylsulfonyl-substituted analogs showed potent and selective COX-2 inhibition [17]. Comparing both substance classes **1a**–**d** and **2a**–**d** of this work, the 1,2-dihydropyrrolo[3,2,1-*hi*]indoles **1a**–**d** were found to be less potent exerting COX-2 inhibition with IC_50_ in the upper nanomolar range (0.092–0.253 µM). Interestingly, a distinct ability to inhibit COX-1 in the micromolar range (7.8–59.9 µM) was found for these compounds that was not present in other (dihydro)pyrrolo[3,2,1-*hi*]indoles [17]. The pyrrolo[3,2,1-*hi*]indoles **2a**–**d** were found to be more potent as well as highly selective COX-2 inhibitors with IC_50_ for COX-2 in a narrow range of 0.053–0.092 µM. The most potent and selective derivative represents the phenyl-derivative **2a** having an IC_50_ for COX-2 of 53 nM. Within the respective sub-class, a general trend for the structure-activity-relationship can’t be deduced from this small set of compounds. However the finding that the pyrrolo[3,2,1-*hi*]indoles **2a**–**d** are more potent (e.g., **1a** vs. **2a**, **1b** vs. **2b**) and selective inhibitors than the respective 1,2-dihydropyrrolo[3,2,1-*hi*]indoles suggests a positive influence of the extended π-system that was similarly found for the methylsulfonyl-substituted analogs [17]. In comparison, the *N*-propionamide substituted analogs **3a**–**d** and **4b**–**d** were found to have considerably lower (**3c**, **4b**–**d**: IC_50_ 11.0–33.9 µM) or even no (**3a**–**b**, **3d**) COX-2 inhibition potency, while all tested compounds did not inhibit COX-1. The loss of COX-2 inhibition potency is in accordance with the role of the sulfonamide group as an important binding motif of COX-2 selective inhibitors [2,12].

The lipophilicity of all synthesized compounds was determined as log*D*_7.4HPLC_ value (Table 1) using an HPLC method originally described by Donovan and Pescatore [18]. All 1,2-dihydropyrrolo[3,2,1-*hi*]indoles **1a**–**d** and pyrrolo[3,2,1-*hi*]indoles **2a**–**d** were found to be highly lipophilic with log*D*_7.4HPLC_ values ranging from 4.44–4.90, while a direct comparison between each pair revealed a higher lipophilicity of the pyrrolo[3,2,1-*hi*]indoles (Δ0.11–0.17) except for **1c** vs. **2c** (Δ–0.01). The introduction of the propionamide-group in **3a**–**d** and **4a**–**d** markedly lowered the lipophilicity by more than two orders of magnitude (Δ2.25 (**2b** vs. **4b)** to Δ2.41 (**1c** vs. **3c**)), resulting in log*D*_7.4HPLC_ values in the range of 2.05–2.60. The reason for that behavior is well known [19] and caused by the weakly acidic *N*-(sulfonyl)propionamide functionality, which is deprotonated under physiological pH, and so the anionic—and hence more hydrophilic—species is formed.

## 3. Discussion

COX-2 represents an interesting target for non-invasive imaging by positron emission tomography because of its clinical relevance in inflammatory diseases and cancer. While for the generation of potent COX-2 inhibitors a variety of strategies are available, the development of a successful radiolabeled probe to target COX-2 has still not been accomplished. Low metabolic stability, fast excretion from the body, and high lipophilicity, which is in principal needed to address the lipophilic binding site in COX-2—and in turn leads to high binding in off-target tissues like white adipose tissue—can be considered as main reasons for the inability of previously synthesized imaging agents to visualize COX-2 in vivo [7,8,9]. In this regard, novel approaches for the development of COX-2 targeted imaging agents are highly needed.

Recently, a potent and selective COX-2 inhibitor having a 1,2-dihydropyrrolo[3,2,1-*hi*]indole core and a methylsulfonyl group was labeled with fluorine-18 by us and evaluated in vitro and in vivo. This tracer failed to visualize COX-2 in vivo because of its fast hepatobiliary excretion as well as high non-specific binding, too [16,17]. In this study we aimed for the investigation of a set of 1,2-dihydropyrrolo[3,2,1-*hi*]indoles and pyrrolo[3,2,1-*hi*]indoles with a sulfonamide-group to overcome the previous drawbacks and find novel leads for the development of radiolabeled COX-2 inhibitors. In general, sulfonamide-substituted COXIBs are commonly known to exert slower blood clearance because of binding to carboanhydrase in the blood pool, but regarding their lipophilicity, they are comparable to their methylsulfonyl-substituted analogs [7,8,9]. Hence, to decrease the lipophilicity of this substance class without compromising the high COX-inhibition potency we aimed to follow the elegant approach of converting the sulfonamides into their respective *N*-propionamides, as previously shown for the clinically approved COXIB parecoxib [19]. Parecoxib sodium represents a water-soluble (>50 mg/mL) and injectable prodrug that is hydrolyzed in vivo to the COX-2 selective inhibitor valdecoxib (4-(5-methyl-3-phenyl-4-isoxazolyl)benzenesulfonamide), which is used, e.g., in the treatment of acute pain [19,20,21,22]. “Inactive prodrugs” like parecoxib are pharmacologically inactive compounds that are converted into an active substance form in the body. Instead of administering a drug directly, an appropriate prodrug can be used instead to improve how a compound is absorbed, distributed, metabolized, and excreted (ADME). A prodrug can be used also to improve how selectively the drug interacts with cells, organs, or processes that are not its target. From the radiochemist’s or radiopharmacologist’s point of view, the terms prodrug—drug or pro-radiotracer—radiotracer are used synonymously here.

Both, 1,2-dihydropyrrolo[3,2,1-*hi*]indoles **1a**–**d** and pyrrolo[3,2,1-*hi*]indoles **2a**–**d** were successfully synthesized by a regioselective, McMurry-based approach that was recently presented by us [17]. As a proof of concept, the *N*-propionamides **3a**–**d** and **4a**–**d** were generated in microscale experiments. While the sulfonamides **1a**–**d** and **2a**–**d** were found to be potent and selective (but highly lipophilic) COX-2 inhibitors, their respective *N*-propionamides **3a**–**d** and **4a**–**d** turned out to be only weak or no COX-2 inhibitors but also showing a significant decreased lipophilicity. This is in accordance with the behavior of valdecoxib (IC_50_ hCOX-2 = 5 nM) and its prodrug parecoxib (IC_50_ hCOX-2 = 20 µM) [19]. Taking into account that only the ^18^F-labeled 1,2-dihydropyrrolo[3,2,1-*hi*]indole but not the respective ^18^F-labeled pyrrolo[3,2,1-*hi*]indole was accessible via radiosynthesis before [16], compound **3d** represents the most promising lead for further PET tracer developments. As a non-COX-active and hydrophilic prodrug this probe could be administered and, hypothetically, be converted into the potent radiolabeled COX-2 inhibitor in the liver to finally distribute comparable to the parent drug afterward. Beside the possibility to administer a more hydrophilic drug intravenously, this can mitigate the release of the original radiotracer in the blood, leading to a retardation of the excretion and hence a longer circulation in the body. Exemplarily, the elimination half-life of parecoxib leading to valdecoxib in rat plasma (T_1/2_ = 0.135 h [19], T_1/2_ = 0.69 h [23]) suggests a suitable conversion rate of the prodrug to the drug within the time window that is available for a radiopharmacological evaluation with fluorine-18 (t_1/2_ ≈ 110 min) or other radionuclides with longer physical half-life, e.g., iodine-123 (t_1/2_ ≈ 13 h). In this sense, the use of *N*-propionamides **3a**–**d** and **4a**–**d** hold promise to act as hydrophilic prodrugs for their respective highly selective COX-2 inhibitors but, although *N*-propionamides are known to be cleaved in vivo, mainly in the liver [19,20], it remains to be verified that this is also true for the *N*-propionamides **3a**–**d** and **4a**–**d**. Moreover, the extent to which the delayed release of the actual radiotracer, which is again characterized by stronger lipophilicity, then influences its availability or on-target enrichment behavior, still has to be investigated.

With respect to radiotracer development, prodrug strategies for the development of COX-targeting imaging agents are rare. Takashima-Hirano et al. [24,25] presented the synthesis of the methyl esters of [^11^C]ibuprofen, [^11^C]naproxen, [^11^C]flurbiprofen, [^11^C]fenoprofen, [^11^C]ketoprofen, and [^11^C]loxoprofen as proradiotracers and exemplarily confirmed that [^11^C]ketoprofen methyl ester enters the brain and is then readily hydrolyzed to [^11^C]ketoprofen, which has a free carboxylic acid group. [^11^C]Celecoxib and its major metabolite [^11^C]SC-62807, bearing a carboxylic acid instead of the methyl group at one phenyl ring, was investigated by Takashima-Hirano et al. with focus on drug transporter function in biliary excretion [26]. In this example, [^11^C]celecoxib acted as a prodrug since only the metabolite was a transporter substrate. In comparison to that, for the development of COX-2 inhibitors a variety of different prodrug approaches, e.g., esters, amides, or hybrid prodrugs (NO-NSAIDs, AChEI-NSAIDs, Phospho-NSAIDs) were already successfully applied [27,28,29], showing the potential of this approach for future developments.

In summary, while sulfonamide-substituted 1,2-dihydropyrrolo[3,2,1-*hi*]indoles **1a**–**d** and pyrrolo[3,2,1-*hi*]indoles **2a**–**d** showed high COX-inhibition potency and selectivity but high lipophilicity that might hinder their future use as radiotracers, the prodrug approach created the respective more hydrophilic analogs **3a**–**d** and **4a**–**d**, which represents a potential approach for more sophisticated radiotracer developments.

## 4. Materials and Methods

All commercial reagents and solvents were used without further purification unless otherwise specified. The 7-acyl-indolines used as starting material for the synthesis of **1a**–**d** were prepared as previously reported [17].

Column chromatography was performed using silica gel (mesh size 40–63 µm). Thin-layer chromatography (TLC) was performed on silica gel F-254 aluminum plates and visualized using UV (254 nm/366 nm). Analytical HPLC analysis was carried out with the following systems: (system 1) Agilent 1200 HPLC (Santa Clara, CA, USA; pump G1311A, autosampler G1329A, column oven G1316A, degasser G1322A, UV detector G1315D, γ detector Gabi Star (Raytest), Luna C18 column (Phenomenex, 250 × 4.6 mm), flow rate = 1 mL/min, isocratic eluent (MeCN/0.1% TFA in H_2_O 70/30 (*v*/*v*)); (system 2) Agilent 1100 HPLC (Santa Clara, CA, USA; binary pump G1312A, autosampler G1313A, column oven G1316A, degasser G1322A, UV detector G1314A, γ detector Gabi Star (Raytest, Straubenhardt, Germany); column ODP-50 4B (Shodex Asahipak 50 × 4.6 mm); eluent: MeOH/PBS (10 mM, pH 7.4) gradient *t*_0 min_ 30/70 - *t*_25 min_ 95/5 - *t*_27 min_ 95/5 - *t*_28 min_ 30/70 - *t*_40 min_ 30/70, flow rate = 0.6 mL/min; (system 3) waters UPLC *I*-Class (Milford, MA, USA; binary gradient pump BSM, autosampler FTN, column manager CM, and diode array detector PDAeλ coupled to Waters Xevo TQ-S), column Aquity UPLC^®^ BEH C18 column (waters, 100 × 2.1 mm, 1.7 µm, 130 Å), eluent: (A): 0.1% acetic acid in MeCN/MeOH 1/1/(B): 0.1% acetic acid in H_2_O; flow rate 0.4 mL/min), gradient: *t*_0 min_ 45/55 - *t*_0.5 min_ 45/55 - *t*_5.5 min_ 95/5 - *t*_7.0 min_ 95/5 - *t*_8.0 min_ 45/55 – *t*_8.5 min_ 45/55; (system 4) column (Kinetex C-18 (Phenomenex 50 × 2.1 mm, 1.7 µm, 100 Å), Shimadzu Nexera X2 UHPLC system (Kyoto, Japan; degasser DGU-20A_3R_ and DGU-20A_5R_, pump LC-30AD, autosampler SIL-30AC, column oven CTO-20AC with two column switching valves FCV-14AH, diode array detector SPD-M30A, γ detector Gabi Star (Raytest, Straubenhardt, Germany), communication bus module CBM-20A), eluent: (A): MeCN, (B): 0.1% trifluoroacetic acid in H_2_O; flow rate 0.5 mL/min), gradient: *t*_0 min_ 25/75 - *t*_0.3 min_ 25/75 - *t*_4.0 min_ 75/25 - *t*_4.5 min_ 95/5 - *t*_5.5 min_ 95/5 - *t*_6.0 min_ 25/75 – *t*_7.5 min_ 25/75. The products were monitored by an UV detector at 254 nm and purity of all compounds exceeded 95% as determined by analytical HPLC analysis (system 1 or system 3), unless otherwise stated. Semi-preparative HPLC was performed using the following system: column (C-18 Jupiter Proteo (Phenomenex 250 × 21.1 mm, 4 µm, 90 Å), Shimadzu prominence modular HPLC system (Kyoto, Japan; degasser DGU-20A_5R_, 2× pump LC-20AR, autosampler SIL-20AC _HT_, column oven CTO-20AC with column switching valve, diode array detector SPD-M20A, fluorescence detector RF-20A, and fraction collector FRC-10A, communication bus module CBM-20A), isocratic eluent 0.1% TFA in MeCN/0.1% TFA in H_2_O 70/30, flow rate = 10 mL/min.

Low resolution mass spectra were obtained using electrospray or ASAP ionization (atmospheric solids analysis probe) using system 3. High resolution mass spectra were obtained on a Q-TOF MS using electrospray ionization: Agilent 1260 Infinity II HPLC (Santa Clara, CA, USA; pump G7111B, autosampler G7129A, column oven G7116N, UV detector G7717C, eluent MeCN/water acidified with 0.1% formic acid, bypass mode) coupled to UHD Accurate Mass Q-TOF LC MS G6538A.

Melting points were determined with a melting point apparatus (Cambridge Instruments, London, UK; Galen_TM_ III, Testotherm testo 700; heater: Leica) and are uncorrected. Nuclear magnetic resonance spectra (NMR) were recorded on a 400 MHz (Varian, Palo Alto, CA, USA; Unity INOVA 400 MHz) spectrometer. NMR spectra were referenced to the residual solvent shifts for ^1^H and ^13^C as internal standard. DHPI and PI served as abbreviations for 1,2-dihydropyrrolo[3,2,1-*hi*]indole and pyrrolo[3,2,1-*hi*]indole, respectively.

### 4.1. Syntheses

General Procedure A—Synthesis of 1,2-dihydropyrrolo[3,2,1-*hi*]indoles **1a**–**d**:

As a starting material, 4-(sulfamoyl)benzoic acid chloride was prepared under Schlenk conditions from 4-(sulfamoyl)benzoic acid (1.504 g, 7.48 mmol, 1.0 equiv) by addition of SOCl_2_ (6.3 mL, 86.7 mmol, 11.5 equiv) followed by heating under reflux (60–70 °C) for 24 h. After removal of SOCl_2_ under reduced pressure, three times a sequence of benzene (8 mL) addition, stirring at room temperature, and removal of solvent was performed to remove traces of SOCl_2_. The resulting yellow solid (1.937 g, purity 85% calculated for quantitative conversion) was used without further purification for the synthesis of **1a−d**.

Under Schlenk conditions, the 7-acyl-indoline (1.26 mmol, 1 equiv) was dissolved in THF (1.6 mL), followed by the addition of triethylamine (199.6 µL, 145 mg, 1.43 mmol, 1.14 equiv) and 4-(sulfamoyl)benzoic acid chloride (303 mg*, 1.38 mmol, 1.1 equiv;*the used amount of crude 4-(sulfamoyl)benzoic acid chloride was corrected for the given calculated purity) in THF (4.8 mL). The mixture was stirred at room temperature for 2 h. Then, THF (3.2 mL), zinc dust (328 mg, 5.02 mmol, 4 equiv) and TiCl_4_ (291.2 µL, 501 mg, 2.64 mmol, 2.1 equiv in four portions) were added and the mixture was heated to 70 °C and stirred for 2 h. After cooling, the mixture was transferred with DCM to a second flask, adsorbed on silica gel and purified by column chromatography to give the title compounds.

*5-Phenyl-4-[4-(sulfamoyl)phenyl]-1,2-dihydropyrrolo[3,2,1-hi]indole* (**1a**): Starting from 7-benzoylindoline (314 mg, 1.41 mmol, 1 equiv), 4-(sulfamoyl)benzoic acid chloride (341 mg, 1.55 mmol, 1.1 equiv), triethylamine (223.4 µL, 162 mg, 1.60 mmol, 1.14 equiv), zinc dust (368 mg, 5.63 mmol, 4 equiv) and TiCl_4_ (326.4 µL, 561 mg, 2.96 mmol, 2.1 equiv in four portions), the product was obtained after purification by column chromatography (1. petroleum ether/EtOAc 7/3 → 2/3; 2. petroleum ether/EtOAc 7/3 → 1/1) and sublimation in vacuo as a dark yellow solid (107 mg, 20%): mp: 220–224 °C; *R*_f_ = 0.53 (petroleum ether/EtOAc 1/2); ^1^H-NMR (400 MHz, (CD_3_)_2_SO): δ = 3.78 (t, ^3^*J*_1,2_ = 7.1 Hz, 2H, H_dhpi H1_), 4.66 (t, ^3^*J*_1,2_ = 6.9 Hz, 2H, H_dhpi H2_), 6.98 (d, ^3^*J*_H,H_ = 6.6 Hz, 1H, H_dhpi_), 7.02 (t, ^3^*J*_H,H_ = 7.6 Hz, ^3^*J*_H,H_ = 6.8 Hz, 1H, H_dhpi H7_), 7.24 (t, ^3^*J*_3,4_ = 6.5 Hz, 1H, H_phenyl H4_), 7.32–7.40 (m, 5H, H_dhpi_/H_phenyl H2/H3/H5/H6_), 7.42 (s, 2H, SO_2_NH_2_), 7.63 (d, ^3^*J*_2,3_ = 8.4 Hz, 1H, H_SO2NH2-phenyl H2/H6_), 7.82 (d, ^3^*J*_2,3_ = 8.5 Hz, 1H, H_SO2NH2-phenyl H3/H5_) ppm; ^13^C-NMR* (101 MHz, (CD_3_)_2_SO): δ = 32.9 (CH_2_), 49.8 (CH_2_), 116.1 (C), 116.4 (CH_dhpi_), 117.5 (C), 119.2 (C), 122.8 (CH_dhpi_), 125.5 (CH_dhpi_), 125.9 (CH_phenyl C4_), 126.1 (2CH_SO2NH2-phenyl_), 128.7 (2CH), 128.8 (2CH), 129.0 (2CH), 133.0 (C), 135.4 (C), 135.6 (C), 142.8 (C), 147.7 (C) ppm, *^13^C NMR was recorded before sublimation in vacuo and contains signals of EtOAc; MS (ASAP^+^): *m*/*z* (%) = 374 (100) [M]^+^, 375 (88) [M + H]^+^; HRMS (ESI/QTOF) *m*/*z:* [M + H]^+^ Calcd for C_22_H_19_N_2_O_2_S 375.1162, Found 375.1162; HPLC: 98.2% (t*_R_* = 7.95 min; system 1); Log*D*_7.4 HPLC_: 4.44 (*t*_R_ = 24.50 ± 0.09 min).

*4-[4-(Sulfamoyl)phenyl]-5-(p-tolyl)-1,2-dihydropyrrolo[3,2,1-hi]indole* (**1b**): Starting from 7-(4-methylbenzoyl)indoline (298 mg, 1.26 mmol, 1 equiv), 4-(sulfamoyl)benzoic acid chloride (303 mg, 1.38 mmol, 1.1 equiv), triethylamine (199.6 µL, 145 mg, 1.43 mmol, 1.14 equiv), zinc dust (328 mg, 5.02 mmol, 4.0 equiv) and TiCl_4_ (291.2 µL, 501 mg, 2.64 mmol, 2.1 equiv in four portions), the product was obtained after purification by column chromatography (petroleum ether/EtOAc 7/3 → 2/3) as a pale yellow solid (297 mg, 61%): mp: 233–237 °C; *R*_f_ = 0.54 (petroleum ether/EtOAc 1/2); ^1^H-NMR (400 MHz, (CD_3_)_2_SO): δ = 2.32 (s, 3H, CH_3_), 3.76 (t, ^3^*J*_1,2_ = 6.9 Hz, 2H, H_dhpi H1_), 4.64 (t, ^3^*J*_1,2_ = 6.9 Hz, 2H, H_dhpi H2_), 6.97 (d, ^3^*J*_H,H_ = 6.5 Hz, 1H, H_dhpi_), 7.01 (t, ^3^*J*_H,H_ = 7.5 Hz, ^3^*J*_H,H_ = 6.8 Hz, 1H, H_dhpi H7_), 7.17 (d, ^3^*J*_2,3_ = 8.2 Hz, 2H, H_tolyl H3/H5_), 7.24 (d, ^3^*J*_2,3_ = 8.1 Hz, 2H, H_tolyl H2/H6_), 7.32 (d, ^3^*J*_H,H_ = 7.6 Hz, 1H, H_dhpi_), 7.41 (s, 2H, SO_2_NH_2_), 7.62 (d, ^3^*J*_2,3_ = 8.4 Hz, 2H, H_SO2NH2-phenyl H2/H6_), 7.82 (d, ^3^*J*_2,3_ = 8.4 Hz, 2H, H_SO2NH2-phenyl H3/H5_) ppm; ^13^C-NMR (101 MHz, (CD_3_)_2_SO): δ = 20.7 (CH_3_), 32.9 (CH_2_), 49.8 (CH_2_), 116.1 (CH_dhpi_), 116.5 (CH_dhpi_), 117.6 (C), 119.2 (C), 122.7 (CH_dhpi_), 125.5 (C), 126.1 (2CH_SO2NH2-phenyl_), 128.6 (2CH), 129.0 (2CH), 129.4 (2CH), 132.6 (C), 132.8 (C), 135.0 (C), 135.6 (C), 142.7 (C), 147.7 (C) ppm; MS (ASAP^+^): *m*/*z* (%) = 388 (100) [M]^+^, 389 (51) [M + H]^+^; HRMS (ESI/QTOF) m/z: [M + H]^+^ Calcd for C_23_H_21_N_2_O_2_S 389.1318, Found 389.1320; HPLC: 99.0% (t*_R_* = 10.05 min; system 1); Log*D*_7.4 HPLC_: 4.56 (*t*_R_ = 25.07 ± 0.04 min).

*5-(4-Chlorophenyl)-4-[4-(sulfamoyl)phenyl]-1,2-dihydropyrrolo[3,2,1-hi]indole* (**1c**): Starting from 7-(4-chlorobenzoyl)indoline (315 mg, 1.22 mmol, 1 equiv), 4-(sulfamoyl)benzoic acid chloride (303 mg, 1.38 mmol, 1.13 equiv), triethylamine (194 µL, 141 mg, 1.39 mmol, 1.14 equiv), zinc dust (319 mg, 4.74 mmol, 3.9 equiv) and TiCl_4_ (283.2 µL, 487 mg, 2.57 mmol, 2.1 equiv in four portions), the product was obtained after purification by column chromatography (petroleum ether/EtOAc 7/3 → 3/2) as a pale yellow solid (182 mg, 36%): mp: 259–265 °C (degradation starting at 265 °C); *R*_f_ = 0.53 (petroleum ether/EtOAc 1/2; ^1^H-NMR (400 MHz, (CD_3_)_2_SO): δ = 3.77 (t, ^3^*J*_1,2_ = 6.7 Hz, 2H, H_dhpi H1_), 4.65 (t, ^3^*J*_1,2_ = 6.8 Hz, 2H, H_dhpi H2_), 6.99 (d, ^3^*J*_H,H_ = 6.7 Hz, 1H, H_dhpi_), 7.04 (t, ^3^*J*_H,H_ = 7.7 Hz, ^3^*J*_H,H_ = 6.8 Hz, 1H, H_dhpi H7_), 7.31–7.38 (m, 3H, 1H_dhpi_/2H_chlorphenyl H3/H5_), 7.42 (d, ^3^*J*_2,3_ = 8.4 Hz, 4H, NH_2_/2H_chlorphenyl H2/H6_), 7.64 (d, ^3^*J*_2,3_ = 8.3 Hz, 2H, H_SO2NH2-phenyl H2/H6_), 7.85 (d, ^3^*J*_2,3_ = 8.3 Hz, 2H, H_SO2NH2-phenyl H3/H5_) ppm; ^13^C-NMR (101 MHz, (CD_3_)_2_SO): δ = 32.9 (CH_2_), 49.7 (CH_2_), 116.0 (C), 116.3 (CH_dhpi_/C)*, 119.0 (C), 123.0 (CH_dhpi_), 125.6 (C), 126.2 (2CH_SO2NH2-phenyl_), 128.8 (2CH), 129.1 (2CH), 130.3 (2CH), 130.4 (CH_dhpi_), 133.3 (C), 134.5 (C), 135.1 (C), 143.0 (C), 147.6 (C) ppm, *two carbon species with identical chemical shift; MS (ASAP^+^): *m*/*z* (%) = 101 (100), 408 (73) [M]^+^, 409 (44) [M + H]^+^; HRMS (ESI/QTOF) m/z: [M + H, ^35^Cl]^+^ Calcd for C_22_H_18_ClN_2_O_2_S 409.0772, Found 409.0773; HPLC: 93.4% (t*_R_* = 11.23 min; system 1); Log*D*_7.4 HPLC_: 4.90 (*t*_R_ = 26.76 ± 0.06 min).

*5-(4-Fluorophenyl)-4-[4-(sulfamoyl)phenyl]-1,2-dihydropyrrolo[3,2,1-hi]indole* (**1d**): Starting from 7-(4-fluorobenzoyl)indoline (304 mg, 1.26 mmol, 1 equiv), 4-(sulfamoyl)benzoic acid chloride (304 mg, 1.38 mmol, 1.1 equiv), triethylamine (200.2 µL, 145 mg, 1.44 mmol, 1.14 equiv), zinc dust (330 mg, 5.05 mmol, 4 equiv) and TiCl_4_ (292 µL, 502 mg, 2.65 mmol, 2.1 equiv in four portions), the product was obtained after purification by column chromatography (petroleum ether/EtOAc 7/3 → 1/1) as a pale yellow solid (328 mg, 66%): mp: 250–252 °C; *R*_f_ = 0.54 (petroleum ether/EtOAc 1/2); ^1^H-NMR (400 MHz, (CD_3_)_2_SO): δ = 3.77 (t, ^3^*J*_1,2_ = 6.9 Hz, 2H, H_dhpi H1_), 4.65 (t, ^3^*J*_1,2_ = 6.9 Hz, 2H, H_dhpi H2_), 6.98 (d, ^3^*J*_H,H_ = 6.7 Hz, 1H, H_dhpi_), 7.03 (t, ^3^*J*_H,H_ = 7.6 Hz, ^3^*J*_H,H_ = 6.8 Hz, 1H, H_dhpi H7_), 7.21 (t, ^3^*J*_2,3_ = 8.9 Hz, ^3^*J*_3,F_ = 8.9 Hz, 2H, H_F-phenyl H3/H5_), 7.33 (d, ^3^*J*_H,H_ = 7.7 Hz, 1H, H_dhpi_), 7.36 (dd, ^3^*J*_2,3_ = 8.8 Hz, ^4^*J*_2,F_ = 5.6 Hz, 2H, H_F-phenyl H2/H6_), 7.42 (s, 2H, SO_2_NH_2_), 7.62 (d, ^3^*J*_2,3_ = 8.5 Hz, 2H, H_SO2NH2-phenyl H3/H5_), 7.84 (d, ^3^*J*_2,3_ = 8.5 Hz, 2H, H_SO2NH2-phenyl H2/H6_) ppm; ^13^C-NMR (101 MHz, (CD_3_)_2_SO): δ = 32.9 (CH_2_), 49.8 (CH_2_), 115.7 (d, ^2^*J*_3,F_ = 21 Hz, CH_F-phenyl C3/C5_), 116.2 (CH_dhpi_), 116.3 (CH_dhpi_), 116.4 (C), 119.1 (C), 122.9 (CH_dhpi_), 125.6 (C), 126.2 (2CH_SO2NH2-phenyl_), 129.0 (2CH_SO2NH2-phenyl_), 130.5 (d, ^3^*J*_2,F_ = 8 Hz, CH_F-phenyl C2/C6_), 132.0 (d, ^4^*J*_1,F_ = 3 Hz, C_F-phenyl C1_), 133.1 (C), 135.2 (C), 142.9 (C), 147.6 (C), 160.6 (d, ^1^*J*_4,F_ = 243 Hz, C_F-phenyl C4_) ppm; ^19^F-NMR (376 MHz, (CD_3_)_2_SO): δ = −116.6 ppm; MS (ASAP^+^): *m*/*z* (%) = 392 (49) [M]^+^, 393 (100) [M + H]^+^; HRMS (ESI/QTOF) m/z: [M + H]^+^ Calcd for C_22_H_18_FN_2_O_2_S 393.1068, Found 393.1066; HPLC: 98.9% (t*_R_* = 8.29 min; system 1); Log*D*_7.4 HPLC_: 4.53 (*t*_R_ = 24.92 ± 0.01 min).

General Procedure B—Synthesis of pyrrolo[3,2,1-*hi*]indoles **2a**–**d**:

Under nitrogen atmosphere, DDQ (142 mg, 0.63 mmol, 3.5 equiv) in benzene (8.5 mL) was added to the 1,2-dihydropyrrolo[3,2,1-*hi*]indole **1a**–**d** (0.18 mmol, 1.0 equiv) in benzene (2 mL) in a Schlenk flask. The mixture was heated to 100°C and stirred at this temperature for 6 h. After cooling, the mixture was transferred with EtOAc (20 mL) into a separation funnel. The organic phase was washed with saturated sodium thiosulfate (20 mL), saturated sodium bicarbonate (20 mL), and brine (10 mL). The aqueous phase was combined and extracted with EtOAc (2 × 20 mL). After drying of the combined organic phase over sodium sulfate, the crude product was adsorbed on silica gel and further purified by column chromatography as given below to give the title compounds **2a**–**d**.

*1-Phenyl-2-[4-(sulfamoyl)phenyl]pyrrolo[3,2,1-hi]indole* (**2a**): Starting from **1a** (64.0 mg, 0.17 mmol, 1 equiv) and DDQ (136 mg, 0.6 mmol, 3.52 equiv), the product was obtained after purification by column chromatography (petroleum ether/EtOAc 7/3) as a yellow solid (59.6 mg, 94%): mp: 218–220°C; *R*_f_ = 0.44 (petroleum ether/EtOAc 1/1); ^1^H-NMR (400 MHz, (CD_3_)_2_)SO): δ = 7.00 (d, ^3^*J*_4,5_ = 3.1 Hz, 1H, H_pi H5_), 7.37 (t, ^3^*J*_3,4_ = 7.1 Hz, 1H, H_phenyl H4_), 7.46 (t, ^3^*J*_H,H_ = 7.4 Hz, ^3^*J*_H,H_ = 7.8 Hz, 2H, H_phenyl H3/H5_), 7.48–7.56 (m, 5H, SO_2_NH_2_/H_pi H7_/H_phenyl H2/H6_), 7.73–7.79 (m, 3H, 1H_pi_/H_SO2NH2-phenyl H2/H6_), 7.81 (d, ^3^*J*_H,H_ = 7.3 Hz, 1H, H_pi_), 7.90 (d, ^3^*J*_2,3_ = 8.4 Hz, 2H, H_SO2NH2-phenyl H3/H5_), 7.94 (d, ^3^*J*_4,5_ = 3.1 Hz, 1H, H_pi H4_) ppm; ^13^C-NMR (101 MHz, (CD_3_)_2_SO): δ = 111.0 (C), 119.7 (CH_pi_), 121.1 (CH_pi_), 122.0 (C), 122.1 (CH_pi_), 123.3 (C), 124.8 (CH_pi_), 125.9 (CH_pi_), 126.4 (2CH_SO2NH2-phenyl_), 127.2 (CH_phenyl C4_), 129.1 (2CH), 129.2 (2CH), 129.7 (2CH), 133.0 (C), 133.9 (C), 133.9 (C), 136.4 (C), 143.7 (C) ppm; MS (ASAP^+^): *m*/*z* (%) = 372 (100) [M]^+^; MS (ASAP^+^): *m*/*z* (%) = 374 (100) [M]^+^, 375 (88) [M + H]^+^; HRMS (ESI/QTOF) *m*/z: [M + H]^+^ Calcd for C_22_H_17_N_2_O_2_S 373.1005, Found 373.1006; HPLC: 99.3% (t*_R_* = 8.77 min; system 1); Log*D*_7.4 HPLC_: 4.59 (*t*_R_ = 25.22 ± 0.02 min).

*2-[4-(Sulfamoyl)phenyl]-1-(p-tolyl)pyrrolo[3,2,1-hi]indole* (**2b**): Starting from **1b** (100 mg, 0.26 mmol, 1 equiv) and DDQ (204 mg, 0.90 mmol, 3.46 equiv), the product was obtained after purification by column chromatography (petroleum ether/EtOAc 7/3) as a beige solid (97 mg, 98%): mp: 233–235°C; *R*_f_ = 0.47 (petroleum ether/EtOAc 1/1); ^1^H-NMR (400 MHz, (CD_3_)_2_SO): δ = 2.36 (s, 3H, CH_3 tolyl_), 6.98 (d, ^3^*J*_4,5_ = 3.1 Hz, 1H, H_pi H5_), 7.26 (d, ^3^*J*_2,3_ = 8.3 Hz, 2H, H_tolyl H3/H5_), 7.39 (d, ^3^*J*_2,3_ = 8.1 Hz, 2H, H_tolyl H2/H6_), 7.46–7.54 (m, ^3^*J*_H,H_ = 7.3 Hz, 3H, SO_2_NH_2_/H_pi H7_), 7.72–7.78 (m, ^3^*J*_2,3_ = 8.4 Hz, 3H, H_pi_/H_SO2NH2-phenyl H2/H6_), 7.80 (d, ^3^*J*_H,H_ = 7.3 Hz, 1H, H_pi_), 7.90 (d, ^3^*J*_2,3_ = 8.3 Hz, 2H, H _SO2NH2-phenyl H3/H5_), 7.93 (d, ^3^*J*_4,5_ = 3.1 Hz, 1H, H_pi H4_) ppm; ^13^C-NMR (101 MHz, (CD_3_)_2_SO): δ = 20.8 (CH_3_), 110.7 (CH_pi_), 119.6 (CH_pi_), 121.1 (CH_pi_), 122.1 (C/C)*, 123.3 (C), 124.6 (CH_pi_), 125.8 (CH_pi_), 126.4 (2CH_SO2NH2-phenyl_), 129.0 (2CH), 129.6 (2CH), 129.7 (2CH), 130.9 (C), 132.7 (C), 134.0 (C), 136.4 (C), 136.5 (C), 143.6 (C) ppm, *two carbon species with identical chemical shift; MS (ASAP^+^): *m*/*z* (%) = 101 (100), 386 (91) [M]^+^, 387 (31) [M + H]^+^; HRMS (ESI/QTOF) *m*/z: [M + H]^+^ Calcd for C_23_H_19_N_2_O_2_S 387.1162, Found 387.1163; HPLC: 99.1% (t*_R_* = 11.47 min; system 1); Log*D*_7.4 HPLC_: 4.73 (*t*_R_ = 25.91 ± 0.01 min).

*1-(4-Chlorophenyl)-2-[4-(sulfamoyl)phenyl]pyrrolo[3,2,1-hi]indole* (**2c**): Starting from **1c** (91 mg, 0.22 mmol, 1 equiv) and DDQ (177 mg, 0.78 mmol, 3.55 equiv), the product was obtained after purification by column chromatography (petroleum ether/EtOAc 4:1 → 7/3) as a pale yellow solid (62 mg, 68%): mp: 270-273°C; *R*_f_ = 0.48 (petroleum ether/EtOAc 1/1); ^1^H-NMR (400 MHz, (CD_3_)_2_SO): δ = 7.00 (d, ^3^*J*_4,5_ = 2.7 Hz, 1H, H_pi H5_), 7.47–7.56 (m, 7H, SO_2_NH_2_/H_pi H7_/H_Cl-phenyl H2/H3/H5/H6_), 7.74–7.80 (m, 3H, H_pi_/H_SO2NH2-phenyl H2/H6_), 7.82 (d, ^3^*J*_H,H_ = 7.3 Hz, 1H, H_pi_), 7.89–7.96 (m, 3H, H_pi H4_/H_SO2NH2-phenyl H3/H5_) ppm; ^13^C-NMR (101 MHz, (CD_3_)_2_SO): δ = 111.1 (CH_pi_), 119.6 (CH_pi_), 121.3 (CH_pi_), 121.7 (C), 121.9 (C), 122.1 (C), 124.8 (CH_pi_), 125.7 (CH_pi_), 126.5 (2CH_SO2NH2-phenyl_), 129.1 (2CH), 129.7 (2CH), 130.9 (2CH), 131.9 (C), 132.9 (C), 133.3 (C), 133.6 (C), 136.3 (C), 143.8 (C) ppm; MS (ASAP^+^): *m*/*z* (%) = 101 (100), 406 (49) [M, ^35^Cl]^+^, 409 (22) [M, ^37^Cl]^+^; HRMS (ESI/QTOF) *m*/z: [M + H, ^35^Cl]^+^ Calcd for C_22_H_16_ClN_2_O_2_S 407.0616, Found 407.0613; HPLC: 97.1% (t*_R_* = 7.07 min; acetonitrile/0.1% TFA in water 80:20, system 1); Log*D*_7.4 HPLC_: 4.89 (*t*_R_ = 26.71 ± 0.01 min).

*1-(4-Fluorophenyl)-2-[4-(sulfamoyl)phenyl]pyrrolo[3,2,1-hi]indole* (**2d**): Starting from **1d** (100 mg, 0.25 mmol, 1 equiv) and DDQ (203 mg, 0.89 mmol, 3.56 equiv), the product was obtained after purification by column chromatography (petroleum ether/EtOAc 7/3 → 3/2 → 1/1) as a pale yellow solid (79 mg, 80%): mp: 258-259 °C; *R*_f_ = 0.49 (petroleum ether/EtOAc 1/1); ^1^H-NMR (400 MHz, (CD_3_)_2_SO): δ = 7.00 (d, ^3^*J*_4,5_ = 3.1 Hz, 1H, H_pi H5_), 7.30 (t, ^3^*J*_2,3_ = 8.9 Hz, ^3^*J*_3,F_ = 8.9 Hz, 2H, H_F-phenyl H3/H5_), 7.47–7.58 (m, ^4^*J*_2,F_ = 5.4 Hz, 5H, 1H_pi H7_/SO_2_NH_2_/H_F-phenyl H2/H6_), 7.73–7.78 (m, ^3^*J*_H,H_ = 7.2 Hz, ^3^*J*_2,3_ = 8.4 Hz, 3H, H_pi_/H_SO2NH2-phenyl H2/H6_), 7.81 (d, ^3^*J*_H,H_ = 7.3 Hz, 1H, H_pi_), 7.91 (d, ^3^*J*_2,3_ = 8.4 Hz, 2H, H_SO2NH2-phenyl H3/H5_), 7.94 (d, ^3^*J*_4,5_ = 3.2 Hz, 1H, H_pi H4_) ppm; ^13^C-NMR (101 MHz, (CD_3_)_2_SO): δ = 111.0 (CH_pi_), 116.0 (d, ^2^*J*_3,F_ = 22 Hz, CH_F-phenyl C3/C5_), 119.5 (CH_pi_), 121.2 (CH_pi_), 121.9 (C), 122.1 (C), 122.2 (C), 124.7 (CH_pi_), 125.9 (CH_pi_), 126.4 (2CH_SO2NH2-phenyl_), 129.6 (2CH_SO2NH2-phenyl_), 130.3 (d, ^4^*J*_1,F_ = 3 Hz, C_F-phenyl C1_), 131.2 (d, ^3^*J*_2,F_ = 8 Hz, CH_F-phenyl C2/C6_), 133.0 (C), 133.7 (C), 136.3 (C), 143.7 (C), 161.4 (d, ^1^*J*_4,F_ = 244 Hz, C_F-phenyl C4_) ppm; ^19^F-NMR (376 MHz, (CD_3_)_2_SO): δ = -110.1 ppm; MS (ASAP^+^): *m*/*z* (%) = 101 (100), 390 (94) [M]^+^, 391 (34) [M + H]^+^; HRMS (ESI/QTOF) m/z: [M + H]^+^ Calcd for C_22_H_16_FN_2_O_2_S 391.0911, Found 391.0906; HPLC: 98.8% (t*_R_* = 9.36 min; system 1); Log*D*_7.4 HPLC_: 4.64 (*t*_R_ = 25.49 ± 0.05 min).

General Procedure C—Synthesis of *N*-propionamide-substituted 1,2-dihydropyrrolo[3,2,1-*hi*]indoles **3a**–**d** and pyrrolo[3,2,1-*hi*]indoles **4a**–**d**.

The (dihydro)pyrrolo[3,2,1-*hi*]indole **1a**–**d** or **2a**–**d** (25 µmol, 1.0 equiv) and 4-dimethylaminopyridine (DMAP, 6.17 mg, 50 µmol, 2 equiv) were dissolved in anhydrous THF (760 µL) and anhydrous DCM (760 µL). Then, propionyl chloride (2.86 µL, 3.06 mg, 33.1 µmol, 1.31 equiv) was added and the solution was stirred at room temperature for 20 min. Reaction control at this time point by HPLC indicated complete consumption of the starting material. Afterwards, the solvent was evaporated at room temperature in a stream of nitrogen and the crude product was redissolved in MeCN/0.1% TFA in water and purified by semi-preparative HPLC (0.1% TFA in MeCN/0.1% TFA in water 70/30 (*v*/*v*)) to give the title compounds **3a**–**d** and **4a**–**d**.

*N-{[4-(5-phenyl-1,2-dihydropyrrolo[3,2,1-hi]indol-4-yl)phenyl]sulfonyl}propionamide* (**3a**): Starting from **1a** (9.46 mg, 25.26 µmol, 1 equiv), DMAP (6.17 mg, 50.53 µmol, 2.0 equiv), and propionyl chloride (2.89 µL, 33.2 µmol, 1.3 equiv) and following general procedure C, the product was obtained after semi-preparative HPLC (*t*_R_ = 15 min) as a yellow solid (3.79 mg, 35%): mp: 212-214 °C, unstable crystal modification melted at 108-111 °C (from lyophilisation); *R*_f_ = 0.29 (n-hexane/EtOAc 1/1); ^1^H-NMR (400 MHz, Chloroform-*d*): δ = 1.12 (t, ^3^*J*_H,H_ = 7.4 Hz, 3H, CH_2_C*H*_3_), 2.33 (q, ^3^*J*_H,H_ = 7.4 Hz, 2H, C*H*_2_CH_3_), 3.84 (t, ^3^*J*_1,2_ = 7.0 Hz, 2H, H_dhpi H1_), 4.61 (t, ^3^*J*_1,2_ = 7.2 Hz, 2H, H_dhpi H2_), 7.01 (dd, ^3^*J*_H,H_ = 6.7 Hz, ^4^*J*_H,H_ = 0.8 Hz, 1H, H_dhpi_), 7.08 (dd, ^3^*J*_H,H_ = 7.9, ^3^*J*_H,H_ = 6.8 Hz, 1H, H_dhpi H7_), 7.28 (t, ^4^*J*_H,H_ = 1.5 Hz, 0.3H, part of H_phenyl H4_)*, 7.35 (t, ^3^*J*_H,H_ = 7.6 Hz, 2H, H_phenyl H3/H5_), 7.41 (dd, ^3^*J*_3,4_ = 8.3 H, ^4^*J*_2,4_ = 1.4 Hz, 2H, H_phenyl H2/H6_), 7.44 (dd, ^3^*J*_H,H_ = 7.9 Hz, ^4^*J*_H,H_ = 0.7 Hz, 1H, H_dhpi_), 7.57 (d, ^3^*J*_H,H_ = 8.8 Hz, 2H, H_SO2-phenyl H2/H6_), 7.94 (s, 1H, NH), 7.99 (d, ^3^*J*_H,H_ = 8.8 Hz, 2H, H_SO2-phenyl H3/H5_) ppm, *part of H_phenyl H4_ signal which overlaps with residual solvent signal of CDCl_3_; ^13^C-NMR (101 MHz, Chloroform-*d*): δ = 8.4 (CH_2_*C*H_3_), 29.8 (*C*H_2_CH_3_), 33.7 (CH_2_), 50.7 (CH_2_), 116.8 (CH_dhpi_), 117.6 (CH_dhpi_), 120.3 (C), 120.5 (C), 123.2 (CH_dhpi_), 125.1 (C), 126.5 (CH_phenyl H4_), 128.8 (2CH_SO2-phenyl_), 128.9 (2CH_phenyl_), 129.2 (2CH_SO2-phenyl_), 129.5 (2CH_phenyl_), 133.0 (C), 135.7 (C), 136.7 (C), 138.9 (C), 148.9 (C), 171.1 (CONH) ppm; MS (ASAP^+^): *m*/*z* (%) = 374 (100) [M]^+^, 375 (88) [M + H]^+^; MS (ESI^+^): *m*/*z* (%) = 296.3 (11) [M + H-SO_2_NHCOC_2_H_5_]^+^, 431.3 (100) [M + H]^+^, 533.9 (15); HRMS (ESI/QTOF) m/z: [M + H]^+^ Calcd for C_25_H_23_N_2_O_3_S 431.1424, Found 431.1416; HPLC: 98.8% (t*_R_* = 4.56 min; system 3); Log*D*_7.4 HPLC_: 2.05 (*t*_R_ = 13.38 ± 0.08 min).

*N-({4-[5-(p-tolyl)-1,2-dihydropyrrolo[3,2,1-hi]indol-4-yl]phenyl}sulfonyl)propionamide* (**3b**): Starting from **1b** (9.52 mg, 24.51 µmol, 1 equiv), DMAP (5.98 mg, 49.01 µmol, 2.0 equiv), and propionyl chloride (2.80 µL, 32.1 µmol, 1.3 equiv) and following general procedure C, the product was obtained after semi-preparative HPLC (*t*_R_ = 19 min) as a yellow solid (4.96 mg, 46%): mp: 116-117 °C (from lyophilisation); *R*_f_ = 0.28 (n-hexane/EtOAc 1/1); ^1^H-NMR (400 MHz, Chloroform-*d*): δ = 1.12 (t, ^3^*J*_H,H_ = 7.4 Hz, 3H, CH_2_C*H*_3_), 2.34 (q, ^3^*J*_H,H_ = 7.4 Hz, 2H, C*H*_2_CH_3_), 2.38 (s, 3H, CH_3_), 3.83 (t, ^3^*J*_1,2_ = 7.0 Hz, 2H, H_dhpi H1_), 4.59 (dd, ^3^*J*_1,2_ = 7.5, 6.5 Hz, 2H, H_dhpi H2_), 7.00 (d, ^3^*J*_H,H_ = 7.1 Hz, 1H, H_dhpi_), 7.07 (dd, ^3^*J*_H,H_ = 7.9, 6.7 Hz, 1H, H_dhpi H7_), 7.16 (d, ^3^*J*_2,3_ = 7.8 Hz, 2H, H_tolyl H3/H5_), 7.30 (d, ^3^*J*_2,3_ = 8.1 Hz, 2H, H_tolyl H2/H6_), 7.43 (dd, ^3^*J*_H,H_ = 7.9, 0.7 Hz, 1H, H_dhpi_), 7.58 (d, ^3^*J*_2,3_ = 8.7 Hz, 2H, H_SO2-phenyl H2/H6_), 7.99 (d, ^3^*J*_2,3_ = 8.6 Hz, 2H, H_SO2-phenyl H3/H5_), 8.01 (s, 1H, NH) ppm; ^13^C-NMR (101 MHz, Chloroform-*d*): δ = 8.4 (CH_2_*C*H_3_), 21.4 (CH_3_), 29.8 (*C*H_2_CH_3_), 33.7 (CH_2_), 50.7 (CH_2_), 116.8 (CH_dhpi_), 117.6 (CH_dhpi_), 120.4 (C), 120.6 (C), 123.0 (CH_dhpi_), 125.0 (C), 128.7 (2CH_SO2-phenyl_), 129.1 (2CH_SO2-phenyl_), 129.4 (2CH_tolyl_), 129.7 (2CH_tolyl_), 132.7 (C), 132.8 (C), 136.2 (C), 136.6 (C), 139.1 (C), 148.9 (C), 171.2 (CONH) ppm; MS (ESI^+^): *m*/*z* (%) = 309.3 (9) [M + H-SO_2_NHCOC_2_H_5_]^+^, 445.5 (100) [M + H]^+^; HRMS (ESI/QTOF) m/z: [M + H]^+^ Calcd for C_26_H_25_N_2_O_3_S 445.1581, Found 445.1576; HPLC: 99.5% (t*_R_* = 4.56 min; system 3); Log*D*_7.4 HPLC_: 2.31 (*t*_R_ = 13.92 ± 0.08 min).

*N-({4-[5-(4-chlorophenyl)-1,2-dihydropyrrolo[3,2,1-hi]indol-4-yl]phenyl}sulfonyl)propionamide* (**3c**): Starting from **1c** (9.32 mg, 22.79 µmol, 1 equiv), DMAP (5.57 mg, 45.59 µmol, 2.0 equiv), and propionyl chloride (2.61 µL, 29.88 µmol, 1.3 equiv) and following general procedure C, the product was obtained after semi-preparative HPLC (*t*_R_ = 20 min) as a yellow solid (5.02 mg, 47%): mp: 232–235 °C, unstable crystal modification melted at 120-122°C (from lyophilisation); *R*_f_ = 0.28 (n-hexane/EtOAc 1/1); ^1^H-NMR (400 MHz, Chloroform-*d*): δ = 1.13 (t, ^3^*J*_H,H_ = 7.4 Hz, 3H, CH_2_C*H_3_*), 2.34 (q, ^3^*J*_H,H_ = 7.4 Hz, 2H, C*H*_2_CH_3_), 3.84 (t, ^3^*J*_1,2_ = 7.0 Hz, 2H, H_dhpi H1_), 4.60 (t, ^3^*J*_1,2_ = 7.0 Hz, 2H, H_dhpi H2_), 7.02 (d, ^3^*J*_H,H_ = 7.0 Hz, 1H, H_dhpi_), 7.09 (dd, ^3^*J*_H,H_ = 7.9, 6.8 Hz, 1H, H_dhpi H7_), 7.27–7.37 (m, 4H, H_Cl-phenyl_), 7.40 (dd, ^3^*J*_H,H_ = 7.9 Hz, ^4^*J*_H,H_ = 0.7 Hz, 1H, H_dhpi_), 7.56 (d, ^3^*J*_2,3_ = 8.8 Hz, 2H, H_SO2-phenyl H2/H6_), 7.98–8.05 (m, 3H, NH/2H_SO2-phenyl H3/H5_) ppm; ^13^C-NMR (101 MHz, Chloroform-*d*): δ = 8.4 (CH_2_*C*H_3_), 29.8 (*C*H_2_CH_3_), 33.7 (CH_2_), 50.7 (CH_2_), 117.0 (CH_dhpi_), 117.3 (CH_dhpi_), 119.1 (C), 120.1 (C), 123.4 (CH_dhpi_), 125.1 (C), 128.9 (2CH_SO2-phenyl_), 129.2 (2CH), 129.2 (2CH), 130.7 (2CH_Cl-phenyl_), 132.2 (C), 133.1 (C), 134.3 (C), 137.1 (C), 138.6 (C), 148.8 (C), 171.2 (CONH) ppm; MS (ESI^+^): *m*/*z* (%) = 329.5 (26) [M + H-SO_2_NHCOC_2_H_5_, ^35^Cl]^+^, 465.2 (100) [M + H, ^35^Cl]^+^; HRMS (ESI/QTOF) m/z: [M + H, ^35^Cl]^+^ Calcd for C_25_H_22_ClN_2_O_3_S 465.1034, Found 465.1029; HPLC: 99.2% (t*_R_* = 5.04 min; system 3); Log*D*_7.4 HPLC_: 2.49 (*t*_R_ = 14.81 ± 0.00 min).

*N-({4-[5-(4-fluorophenyl)-1,2-dihydropyrrolo[3,2,1-hi]indol-4-yl]phenyl}sulfonyl)propionamide* (**3d**): Starting from **1d** (10.19 mg, 25.97 µmol, 1 equiv), DMAP (6.34 mg, 51.93 µmol, 2.0 equiv), and propionyl chloride (2.97 µL, 34.04 µmol, 1.3 equiv) and following general procedure C, the product was obtained after semi-preparative HPLC (*t*_R_ = 15 min) as a pale yellow solid (4.70 mg, 40%): mp: 119-126 °C (from lyophilisation); *R*_f_ = 0.26 (n-hexane/EtOAc 1/1); ^1^H-NMR (400 MHz, Chloroform-*d*): δ = 1.13 (t, ^3^*J*_H,H_ = 7.4 Hz, 3H, CH_2_C*H*_3_), 2.33 (q, ^3^*J*_H,H_ = 7.4 Hz, 2H, C*H*_2_CH_3_), 3.84 (t, ^3^*J*_1,2_ = 7.0 Hz, 2H, H_dhpi H1_), 4.60 (t, ^3^*J*_1,2_ = 7.0 Hz, 2H, H_dhpi H2_), 7.00–7.11 (m, 4H, 2H_dhpi_/H_F-phenyl H3/H5_), 7.35 (dd, ^3^*J*_2,3_ = 8.9 Hz, ^4^*J*_2,F_ = 5.4 Hz, 2H, H _F-phenyl H2/H6_), 7.39 (d, ^3^*J*_H,H_ = 8.2 Hz, 1H, H_dhpi_), 7.55 (d, ^3^*J*_2,3_ = 8.8 Hz, 2H, H_SO2-phenyl H2/H6_), 7.94 (s, 1H, NH), 8.00 (d, ^3^*J*_2,3_ = 8.8 Hz, 2H, H_SO2-phenyl H3/H5_) ppm; ^13^C-NMR (101 MHz, Chloroform-*d*): δ = 8.4 (CH_2_*C*H_3_), 29.8 (*C*H_2_CH_3_), 33.7 (CH_2_), 50.7 (CH_2_), 115.9 (d, ^2^*J*_3,F_ = 22 Hz, CH_F-phenyl C3/C5_), 116.9 (CH_dhpi_), 117.3 (CH_dhpi_), 119.4 (C), 120.3 (C), 123.3 (CH_dhpi_), 125.1 (C), 128.9 (2CH_SO2-phenyl_), 129.1 (2CH_SO2-phenyl_), 131.0 (d, ^3^*J*_2,F_ = 8 Hz, CH_F-phenyl C2/C6_), 131.7 (d, ^4^*J*_1,F_ = 3 Hz, C_F-phenyl C1_), 133.0 (C), 136.9 (C), 138.7 (C), 148.8 (C), 171.1 (CONH) ppm, signal of 1 quaternary carbon not resolved; ^19^F-NMR (376 MHz, Chloroform-*d*): δ = −116.0 ppm; MS (ESI^+^): *m*/*z* (%) = 313.3 (26) [M + H-SO_2_NHCOC_2_H]^+^, 449.1 (100) [M + H]^+^; HRMS (ESI/QTOF) m/z: [M + H]^+^ Calcd for C_25_H_22_FN_2_O_3_S 449.1330, Found 449.1329; HPLC: 99.6% (t*_R_* = 4.64 min; system 3); Log*D*_7.4 HPLC_: 2.20 (*t*_R_ = 13.38 ± 0.08 min).

*N-{[4-(1-phenylpyrrolo[3,2,1-hi]indol-2-yl)phenyl]sulfonyl}propionamide* (**4a**): Starting from **2a** (0.86 mg, 2.31 µmol, 1 equiv), DMAP (0.56 mg, 4.62 µmol, 2.0 equiv), and propionyl chloride (0.26 µL, 3.02 µmol, 1.3 equiv) and following general procedure C, the conversion as indicated by UPLC was incomplete after the indicated reaction time. After portionwise addition of further propionyl chloride (4× 0.26 µL, 3.02 µmol, 1.3 equiv, then 1× 40 µL, 464 µmol, 200 equiv) and DMAP (0.56 mg, 4.62 µmol, 2.0 equiv) and stirring at room temperature for 20 min intervals, the reaction was stopped and the product was obtained after semi-preparative HPLC (*t*_R_ = 17 min) as a solid (30% conversion based on HPLC system 4): *R*_f_ = 0.34 (n-hexane/EtOAc 1/1); MS (ESI^+^): *m*/*z* (%) = 293.2 (100) [M + H-SO_2_NHCOC_2_H]^+^, 429.2 (77) [M + H]^+^, 451.2 (58) [M + Na]^+^; HRMS (ESI/QTOF) m/z: [M + H]^+^ Calcd for C_25_H_21_N_2_O_3_S 429.1268, Found 429.1262; HPLC: 100% (t*_R_* = 4.76 min; system 3); Log*D*_7.4 HPLC_: 2.24 (*t*_R_ = 13.56 ± 0.05 min).

*N-({4-[1-(p-tolyl)pyrrolo[3,2,1-hi]indol-2-yl]phenyl}sulfonyl)propionamide* (**4b**): Starting from **2b** (11.27 mg, 29.16 µmol, 1 equiv), DMAP (7.12 mg, 58.32 µmol, 2.0 equiv), and propionyl chloride (3.33 µL, 38.20 µmol, 1.3 equiv) and following general procedure C, the product was obtained after semi-preparative HPLC (*t*_R_ = 20 min) as a yellow solid (5.44 mg, 42%): mp: 110-115 °C (from lyophilisation); *R*_f_ = 0.35 (n-hexane/EtOAc 1/1); ^1^H-NMR (400 MHz, (Chloroform-*d*): δ = 1.13 (t, ^3^*J*_H,H_ = 7.4 Hz, 3H), 2.35 (q, ^3^*J*_H,H_ = 7.4 Hz, 2H, CH_2_C*H*_3_), 2.41 (s, 3H, CH_3_), 6.89 (d, ^3^*J*_4,5_ = 3.1 Hz, 1H, H_pi_), 7.22 (d, ^3^*J*_2,3_ = 7.8 Hz, 2H, H_tolyl_), 7.39 (d, ^3^*J*_2,3_ = 8.0 Hz, 2H, H_tolyl_), 7.51 (t, ^3^*J*_H,H_ = 7.4 Hz, 1H, H_pi H7_), 7.57 (d, ^3^*J*_4,5_ = 3.1 Hz, 1H, H_pi_), 7.73 (d, ^3^*J*_2,3_ = 8.8 Hz, 2H, H_SO2-phenyl_)*, 7.73–7.80 (m, 2H, 2H_pi_), 8.04–8.11 (m, 3H, NH/H_SO2-phenyl_)* ppm, *integral 1.5 H, two further aromatic signals at 7.59 (d, *J* = 8.2 Hz, ‘0.6H’) and 7.97 (d, *J* = 8.5 Hz, ‘0.6H’) were detected that account for the 2-phenyl ring of the deprotonated species; MS (ESI^+^): *m*/*z* (%) = 307.2 (98) [M + H-SO_2_NHCOC_2_H]^+^, 443.2 (100) [M + H]^+^; HRMS (ESI/QTOF) m/z: [M + H]^+^ Calcd for C_26_H_22_N_2_O_3_S 443.1424, Found 443.1418; HPLC: 99.7% (t*_R_* = 5.12 min; system 3); Log*D*_7.4 HPLC_: 2.48 (*t*_R_ = 14.75 ± 0.01 min).

*N-({4-[1-(4-chlorophenyl)pyrrolo[3,2,1-hi]indol-2-yl]phenyl}sulfonyl)propionamide* (**4c**): Starting from **2c** (1.01 mg, 2.48 µmol, 1 equiv), DMAP (0.61 mg, 4.96 µmol, 2.0 equiv), and propionyl chloride (0.28 µL, 3.25 µmol, 1.3 equiv) and following general procedure C, the conversion as indicated by UPLC was incomplete after the indicated reaction time. After addition of further propionyl chloride (1 × 40 µL, 464 µmol, 187 equiv) and DMAP (0.56 mg, 4.62 µmol, 2.0 equiv) and stirring at room temperature for 20 min, the reaction was stopped and the product was obtained after semi-preparative HPLC (*t*_R_ = 22 min) as a yellow solid (0.26 mg, 23%): *R*_f_ = 0.34 (n-hexane/EtOAc 1/1); MS (ESI^+^): *m*/*z* (%) = 327.2 (100) [M + H-SO_2_NHCOC_2_H_5_, ^35^Cl]^+^, 409.1 (91), 463.1 (78) [M + H, ^35^Cl]^+^; 485.1 (93) [M + Na, ^35^Cl]^+^; HRMS (ESI/QTOF) m/z: [M + H, ^35^Cl]^+^ Calcd for C_25_H_20_ClN_2_O_3_S 463.0878, Found 463.0875; HPLC: 100% (t*_R_* = 5.17 min; system 3); Log*D*_7.4 HPLC_: 2.60 (*t*_R_ = 15.37 min).

*N-({4-[1-(4-fluorophenyl)pyrrolo[3,2,1-hi]indol-2-yl]phenyl}sulfonyl)propionamide* (**4d**): Starting from **2d** (11.62 mg, 29.76 µmol, 1 equiv), DMAP (7.27 mg, 59.52 µmol, 2.0 equiv), and propionyl chloride (3.40 µL, 38.99 µmol, 1.3 equiv) and following general procedure C, the product was obtained after semi-preparative HPLC (*t*_R_ = 17 min) as a yellow solid (5.32 mg, 40%): mp: 202–206 °C; *R*_f_ = 0.35 (n-hexane/EtOAc 1/1); ^1^H-NMR (400 MHz, Chloroform-*d*): δ = 1.14 (t, ^3^*J*_H,H_ = 7.4 Hz, 3H, CH_2_C*H*_3_), 2.34 (q, ^3^*J*_H,H_ = 7.3 Hz, 2H, C*H*_2_CH_3_), 6.91 (d, ^3^*J*_4,5_ = 3.1 Hz, 1H, H_pi_), 7.11 (t, ^3^*J*_2,3_ = ^3^*J*_3,F_ = 8.8 Hz, 2H, H_F-phenyl H3/H5_), 7.46 (dd, ^3^*J*_2,3_ = 8.8, ^4^*J*_2,F_ = 5.4 Hz, 2H, H_F-phenyl H2/H6_), 7.52 (t, ^3^*J*_H,H_ = 7.4 Hz, 1H, H_pi H7_), 7.57 (d, ^3^*J*_4,5_ = 3.1 Hz, 1H, H_pi_), 7.69–7.76 (m, 3H, H_pi_/H_SO2-phenyl H2/H6_), 7.78 (d, ^3^*J*_H,H_ = 7.3 Hz, 1H, H_pi_), 8.00 (s, 1H, NH), 8.09 (d, ^3^*J*_H,H_ = 8.8 Hz, 2H, H_SO2-phenyl H3/H5_) ppm; ^13^C-NMR (101 MHz, Chloroform-*d*): δ = 8.4 (CH_2_*C*H_3_), 29.8 (*C*H_2_CH_3_), 111.3 (CH_pi_), 116.2 (d, ^2^*J*_3,F_ = 22 Hz, CH_F-phenyl C3/C5_), 120.1 (CH_pi_), 121.7 (CH_pi_), 122.6 (C), 122.7 (C), 124.3 (C), 124.8 (CH_pi_), 124.9 (CH_pi_), 129.1 (CH_SO2-phenyl_), 129.7 (CH_SO2-phenyl_), 130.3 (d, ^4^*J*_1,F_ = 3 Hz, C_F-phenyl C1_), 131.3 (d, ^3^*J*_2,F_ = 8 Hz, CH_F-phenyl C2/C6_), 132.6 (C), 137.5 (C), 137.8 (C), 162.3 (d, ^1^*J*_4,F_ = 248 Hz, C_F-phenyl C4_), 171.2 (CONH) ppm; ^19^F-NMR (376 MHz, (CD_3_)_2_SO): δ = −114.3 ppm*, signal of trifluoroacetic acid visible at 76.7 ppm with a molar ratio of product/TFA = 71/1; MS (ESI^+^): *m*/*z* (%) = 311.3 (100) [M + H-SO_2_NH_2_COC_2_H]^+^, 447.2 (64) [M + H]^+^; HRMS (ESI/QTOF) m/z: [M + H]^+^ Calcd for C_25_H_20_FN_2_O_3_S 447.1173, Found 447.1171; HPLC: 99.9% (t*_R_* = 4.79 min; system 3); Log*D*_7.4 HPLC_: 2.30 (*t*_R_ = 13.90 ± 0.03 min).

### 4.2. Lipophilicity

The log*D*_7.4HPLC_ value was determined as previously reported by us [16] utilizing an HPLC method originally described by Donovan and Pescatore[18]. Hydrocortisone (*t*_R_ 10.65 min, log*D*_7.4_ 1.65), toluene (*t*_R_ 16.41 min, log*D*_7.4_ 2.85), and triphenylene (*t*_R_ 29.69 min, log*D*_7.4_ 5.49) served as references using HPLC system 2.

### 4.3. COX Inhibition Studies

The COX inhibition potency against ovine COX-1 and human COX-2 was determined using the fluorescence-based COX assay “COX Fluorescent Inhibitor Screening Assay Kit” (catalog number 700100; Cayman Chemical, Ann Arbor, MI, USA) according to the manufacturer’s instructions as previously reported by us [17].

## Figures and Tables

**Table 1:** COX-inhibitory activity of 1,2-dihydropyrrolo[3,2,1-*hi*]indoles and pyrrolo[3,2,1-*hi*]indoles.

			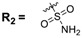					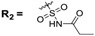			
			IC_50_ [µM]			Log*D*		IC_50_ [µM]			Log*D*
	R_1_	No.	COX-1	COX-2	SI *	_pH 7.4_ _HPLC_	No.	COX-1	COX-2	SI *	_pH 7.4_ _HPLC_
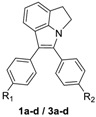	H	**1a**	38	0.107	355	4.44	**3a**	>100 **	>100 **	-	2.05
	CH_3_	**1b**	59.9	0.253	237	4.56	**3b**	>100 **	>100 **	-	2.31
	Cl	**1c**	15.3	0.106	144	4.90	**3c**	>100	26.4	>3.8	2.49
	F	**1d**	7.8	0.092	85	4.53	**3d**	>100 **	>100 **	-	2.20
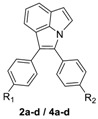	H	**2a**	>100	0.053	>1887	4.59	**4a**	n.d.	n.d.	-	2.24
	CH_3_	**2b**	>100	0.092	>1087	4.73	**4b**	>100	33.9	>2.9	2.48
	Cl	**2c**	>100	0.088	>1136	4.89	**4c**	>100	11.0	>9.1	2.60
	F	**2d**	>100	0.089	>1124	4.64	**4d**	>100	26.5	>3.8	2.30
	**Celecoxib**		>100	0.038 ± 0.020	>2631						

* SI: Selectivity index, SI = IC_50_ (COX-1)/IC_50_ (COX-2); ** compounds were screened at a concentration of 100 µM; n.d. not determined.

## Supplementary Materials

Copies of NMR and HRMS spectra as well as exemplary LC-MS data of the optimization (*N*-propionamide synthesis) are available online.

## References

[1] Qiu J., Shi Z., Jiang J. (2017). Cyclooxygenase-2 in glioblastoma multiforme. Drug Discov. Today.

[2] Carullo G., Galligano F., Aiello F. (2017). Structure–activity relationships for the synthesis of selective cyclooxygenase 2 inhibitors: An overview (2009–2016). MedChemComm.

[3] Laube M., Kniess T., Pietzsch J. (2016). Development of antioxidant cox-2 inhibitors as radioprotective agents for radiation therapy—a hypothesis-driven review. Antioxidants.

[4] Marnett L.J. (2012). Inflammation and cancer: Chemical approaches to mechanisms, imaging, and treatment. J. Org. Chem..

[5] Ghosh N., Chaki R., Mandal V., Mandal S.C. (2010). Cox-2 as a target for cancer chemotherapy. Pharmacol. Rep..

[6] Marnett L.J. (2009). The coxib experience: A look in the rearview mirror. Annu. Rev. Pharmacol. Toxicol..

[7] Pacelli A., Greenman J., Cawthorne C., Smith G. (2014). Imaging cox-2 expression in cancer using pet/spect radioligands: Current status and future directions. J. Label. Compd. Radiopharm..

[8] Tietz O., Marshall A., Wuest M., Wang M., Wuest F. (2013). Radiotracers for molecular imaging of cyclooxygenase-2 (cox-2) enzyme. Curr. Med. Chem..

[9] Laube M., Kniess T., Pietzsch J. (2013). Radiolabeled cox-2 inhibitors for non-invasive visualization of cox-2 expression and activity—A critical update. Molecules.

[10] Prabhakaran J., Underwood M., Zanderigo F., Simpson N.R., Cooper A.R., Matthew J., Rubin-Falcone H., Parsey R.V., Mann J.J., Dileep Kumar J.S. (2018). Radiosynthesis and in vivo evaluation of [11c]mov as a pet imaging agent for cox-2. Bioorg. Med. Chem. Lett..

[11] Elie J., Vercouillie J., Arlicot N., Lemaire L., Bidault R., Bodard S., Hosselet C., Deloye J.B., Chalon S., Emond P. (2019). Design of selective cox-2 inhibitors in the (aza)indazole series. Chemistry, in vitro studies, radiochemistry and evaluations in rats of a [(18)f] pet tracer. J. Enzyme Inhib. Med. Chem..

[12] Zarghi A., Arfaei S. (2011). Selective cox-2 inhibitors: A review of their structure-activity relationships. Iran. J. Pharm. Res..

[13] Tondera C., Ullm S., Laube M., Meister S., Neuber C., Mosch B., Kniess T., Pietzsch J. (2015). Optical imaging of cox-2: Studies on an autofluorescent 2,3-diaryl-substituted indole-based cyclooxygenase-2 inhibitor. Biochem. Biophys. Res. Commun..

[14] Laube M., Tondera C., Sharma S.K., Bechmann N., Pietzsch F.-J., Pigorsch A., Kockerling M., Wuest F., Pietzsch J., Kniess T. (2014). 2,3-diaryl-substituted indole based cox-2 inhibitors as leads for imaging tracer development. RSC Adv..

[15] Kniess T., Laube M., Bergmann R., Sehn F., Graf F., Steinbach J., Wuest F., Pietzsch J. (2012). Radiosynthesis of a 18f-labeled 2,3-diarylsubstituted indole via mcmurry coupling for functional characterization of cyclooxygenase-2 (cox-2) in vitro and in vivo. Bioorg. Med. Chem..

[16] Gassner C., Neuber C., Laube M., Bergmann R., Kniess T., Pietzsch J. (2016). Development of a 18f-labeled diaryl-substituted dihydropyrrolo[3,2,1-*hi*]indole as potential probe for functional imaging of cyclooxygenase-2 with pet. ChemistrySelect.

[17] Laube M., Gassner C., Sharma S.K., Gunther R., Pigorsch A., Konig J., Kockerling M., Wuest F., Pietzsch J., Kniess T. (2015). Diaryl-substituted (dihydro)pyrrolo[3,2,1-hi]indoles, a class of potent cox-2 inhibitors with tricyclic core structure. J. Org. Chem..

[18] Donovan S.F., Pescatore M.C. (2002). Method for measuring the logarithm of the octanol-water partition coefficient by using short octadecyl-poly(vinyl alcohol) high-performance liquid chromatography columns. J. Chromatogr. A.

[19] Talley J.J., Bertenshaw S.R., Brown D.L., Carter J.S., Graneto M.J., Kellogg M.S., Koboldt C.M., Yuan J., Zhang Y.Y., Seibert K. (2000). N-[[(5-methyl-3-phenylisoxazol-4-yl)-phenyl]sulfonyl]propanamide, sodium salt, parecoxib sodium: A potent and selective inhibitor of cox-2 for parenteral administration. J. Med. Chem..

[20] Larsen J.D., Bundgaard H., Lee V.H.L. (1988). Prodrug forms for the sulfonamide group .II. Water-soluble amino-acid derivatives of n-methylsulfonamides as possible prodrugs. Int. J. Pharm..

[21] Teagarden D.L., Nema S., Stella V.J., Borchardt R.T., Hageman M.J., Oliyai R., Maag H., Tilley J.W. (2007). Case study: Parecoxib: A prodrug of valdecoxib. Prodrugs: Challenges and Rewards Part 1.

[22] Karim A., Laurent A., Slater M.E., Kuss M.E., Qian J., Crosby-Sessoms S.L., Hubbard R.C. (2001). A pharmacokinetic study of intramuscular (i.M.) parecoxib sodium in normal subjects. J. Clin. Pharmacol..

[23] Liu M., Yu Q., Li P., Zhu M., Fang M., Sun B., Sun M., Sun Y., Zhang P., He Z. (2016). Simultaneous determination of parecoxib sodium and its active metabolite valdecoxib in rat plasma by uplc-ms/ms and its application to a pharmacokinetic study after intravenous and intramuscular administration. J. Chromatogr. B Analyt. Technol. Biomed. Life Sci..

[24] Takashima-Hirano M., Shukuri M., Takashima T., Goto M., Wada Y., Watanabe Y., Onoe H., Doi H., Suzuki M. (2010). General method for the 11c-labeling of 2-arylpropionic acids and their esters: Construction of a pet tracer library for a study of biological events involved in coxs expression. Chem. Eur. J..

[25] Ohnishi A., Senda M., Yamane T., Sasaki M., Mikami T., Nishio T., Ikari Y., Nishida H., Shukuri M., Takashima T. (2014). Human whole-body biodistribution and dosimetry of a new pet tracer, [(11)c]ketoprofen methyl ester, for imagings of neuroinflammation. Nucl. Med. Biol..

[26] Takashima-Hirano M., Takashima T., Katayama Y., Wada Y., Sugiyama Y., Watanabe Y., Doi H., Suzuki M. (2011). Efficient sequential synthesis of pet probes of the cox-2 inhibitor 11c celecoxib and its major metabolite 11c sc-62807 and in vivo pet evaluation. Bioorg. Med. Chem..

[27] Qandil A.M. (2012). Prodrugs of nonsteroidal anti-inflammatory drugs (nsaids), more than meets the eye: A critical review. Int. J. Mol. Sci..

[28] Lee Y., Kim J., Kim H., Kang S., Yoon J.H., Kim D.D., Kim Y.M., Jung Y. (2012). N-succinylaspart-1-yl celecoxib is a potential colon-specific prodrug of celecoxib with improved therapeutic properties. J. Pharm. Sci..

[29] Jornada D.H., dos Santos Fernandes G.F., Chiba D.E., de Melo T.R., dos Santos J.L., Chung M.C. (2016). The prodrug approach: A successful tool for improving drug solubility. Molecules.

