# Supplementary material for: Synthesis and Cyclooxygenase Inhibition of Sulfonamide-Substituted (Dihydro)Pyrrolo[3,2,1-hi]indoles and Their Potential Prodrugs
Source: Molecules. 2019 Oct 22;24(20):3807. doi: 10.3390/molecules24203807 (PMC6832141; doi:10.3390/molecules24203807)
Supplement: Supplementary file 1 [file molecules-24-03807-s001.pdf]

*molecules*

*Communication*

# Synthesis and Cyclooxygenase Inhibition of Sulfonamide-Substituted (Dihydro)Pyrrolo[3,2,1-*hi*]indoles and Their Potential Prodrugs

# Supporting Information

**Markus Laube [1,*], Cemena Gassner [1], Torsten Kniess [1] and Jens Pietzsch [1,2,*]**

[1] Department of Radiopharmaceutical and Chemical Biology, Institute of Radiopharmaceutical Cancer Research, Helmholtz-Zentrum Dresden-Rossendorf, Bautzner Landstrasse 400, 01328 Dresden, Germany; m.laube@hzdr.de (M.L.); c.gassner@hzdr.de (C.G.); t.kniess@hzdr.de (T.K.); j.pietzsch@hzdr.de (J.P.)

[2] Faculty of Chemistry and Food Chemistry, School of Science, Technische Universität Dresden, Mommsenstrasse 4, 01062 Dresden, Germany; j.pietzsch@hzdr.de (J.P.)

* Correspondence: m.laube@hzdr.de (M.L.); j.pietzsch@hzdr.de (J.P.); Tel.: +49-351-260-2810 (M.L.); Tel.: +49-351-260-2622 (J.P.)

**Table of contents**

Copies of NMR and HRMS data ........................................ 2

Optimization of *N*-propionamide formation ........................................ 31

## Copies of NMR and HRMS data

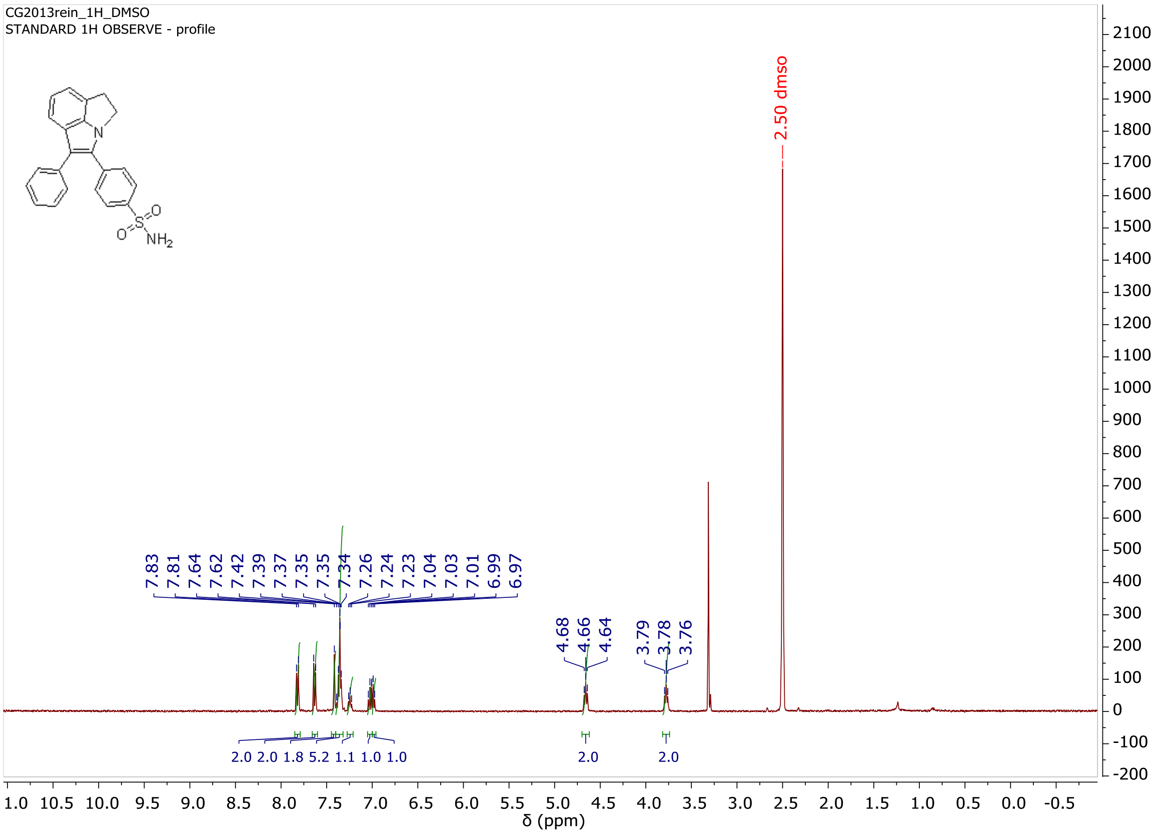


Figure S1. $^{1}$H NMR spectrum of compound **1a** in DMSO-*d*6

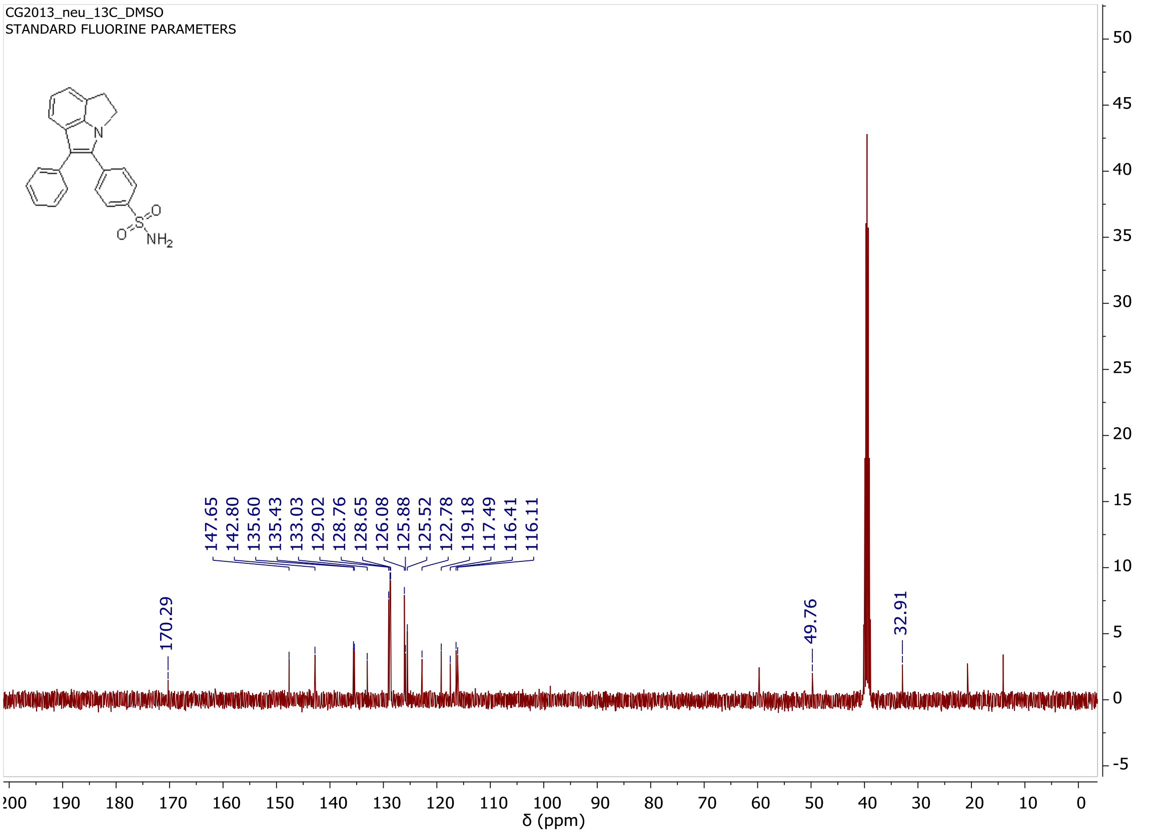


Figure S2. $^{13}$C NMR spectrum of compound **1a** in DMSO-*d*6

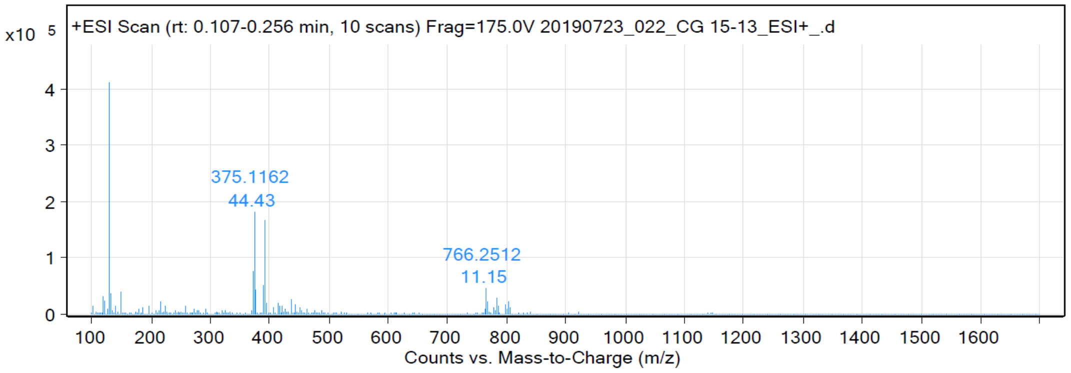


**Peak List**

| *m/z* | z | Abund |
|---|---|---|
| 130.1591 | 1 | 412724.19 |
| 131.1625 | 1 | 36210.37 |
| 149.0234 | | 40093.52 |
| 371.3158 | 1 | 76414.01 |
| 375.1162 | 1 | 183374.05 |
| 376.1195 | 1 | 44520 |
| 388.3421 | 1 | 51474.57 |
| 391.2844 | 1 | 169154.28 |
| 392.2877 | 1 | 45174.34 |
| 766.2512 | 1 | 46024.07 |

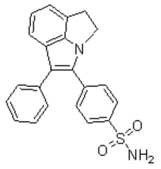


Figure S3. HRMS spectrum of compound **1a**

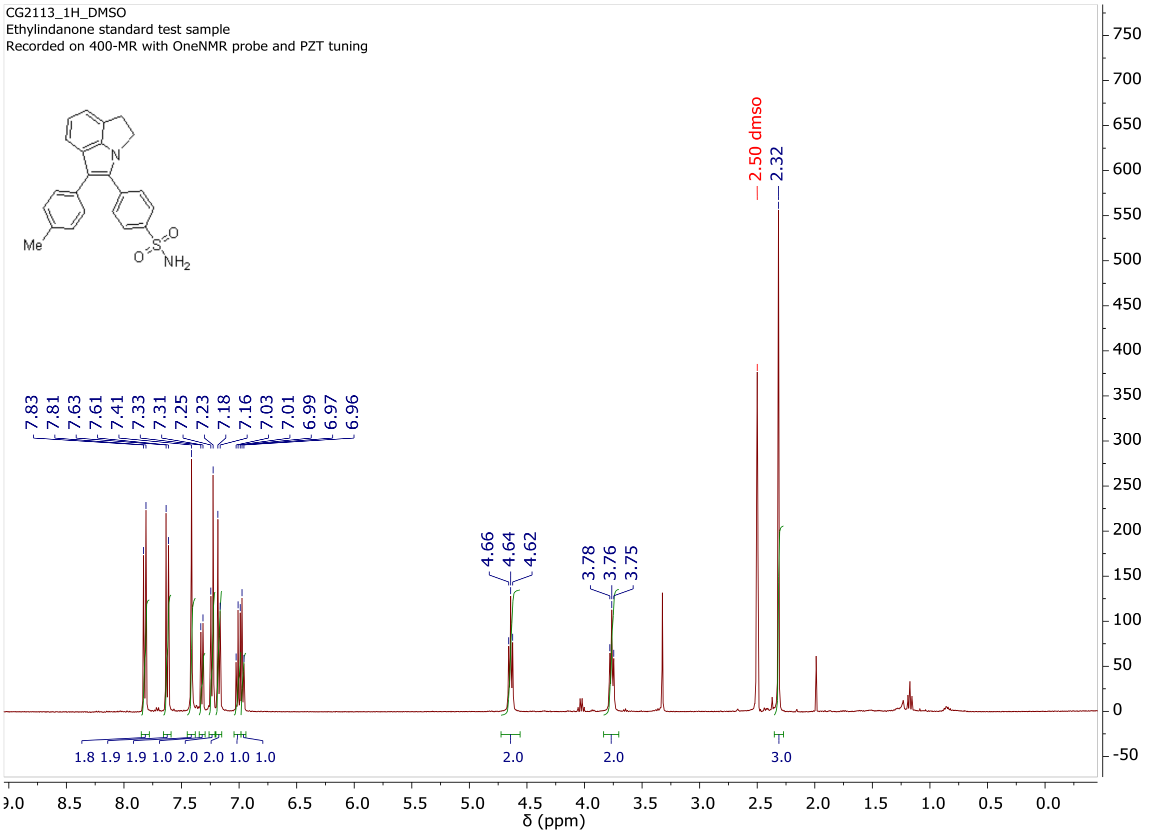


Figure S4. $^1$H NMR spectrum of compound **1b** in DMSO-$d_6$

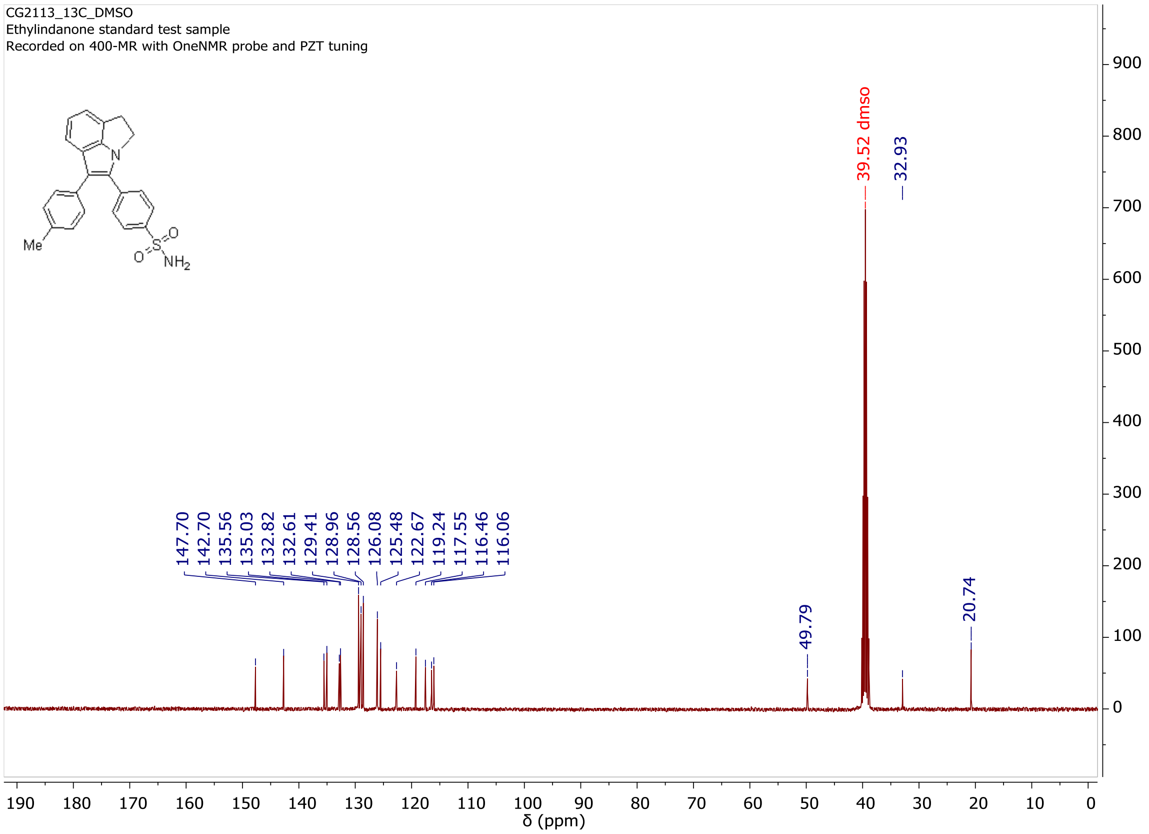


Figure S5. $^{13}$C NMR spectrum of compound **1b** in DMSO-$d_6$

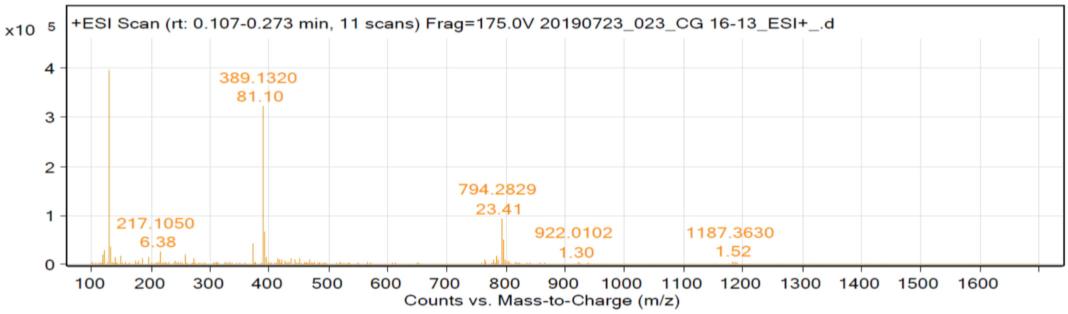


**Peak List**

| *m/z* | z | Abund |
|---|---|---|
| 121.0509 | | 28535.96 |
| 130.1592 | 1 | 396068.91 |
| 131.1626 | 1 | 36811.17 |
| 217.105 | | 25287.13 |
| 371.316 | 1 | 43780.61 |
| 389.132 | 1 | 321212.09 |
| 390.1352 | 1 | 81011.3 |
| 391.2845 | 1 | 67414.38 |
| 794.2829 | 1 | 92729.88 |
| 795.286 | 1 | 49416.65 |

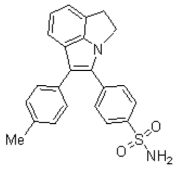


Figure S6. HRMS spectrum of compound **1b**

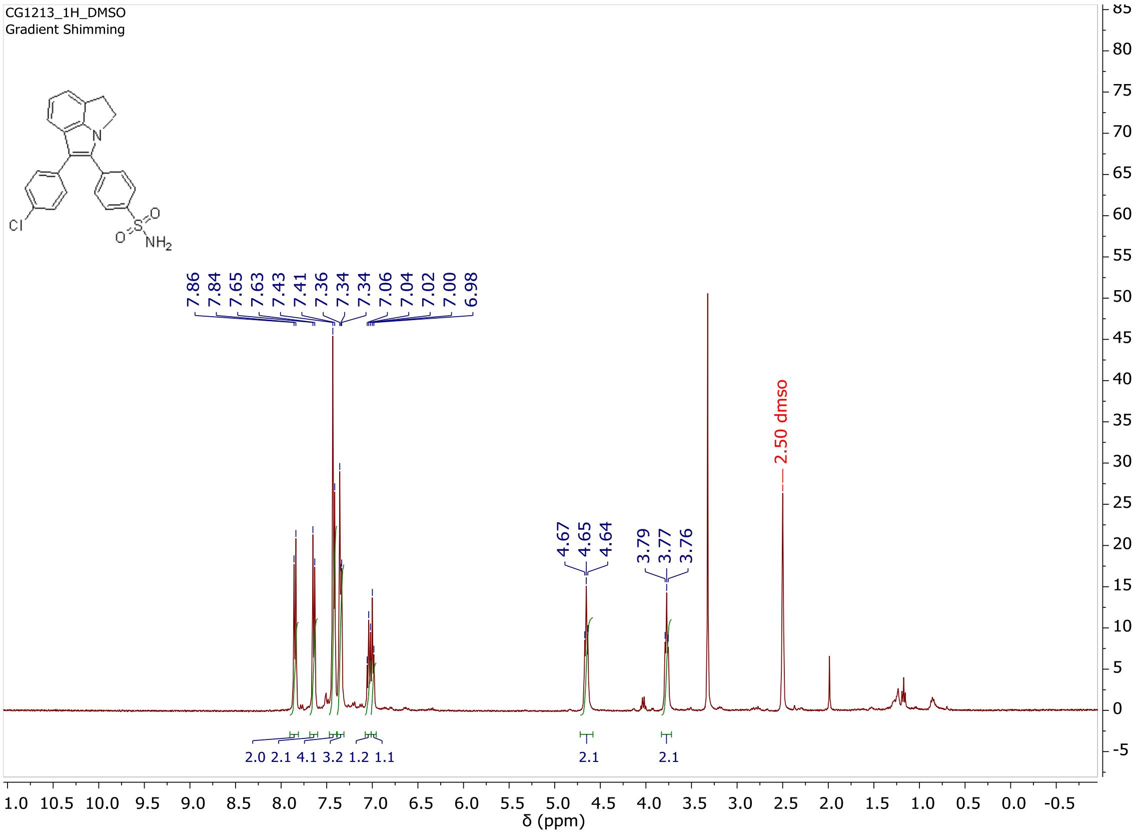


Figure S7. $^{1}$H NMR spectrum of compound **1c** in DMSO-*d*6

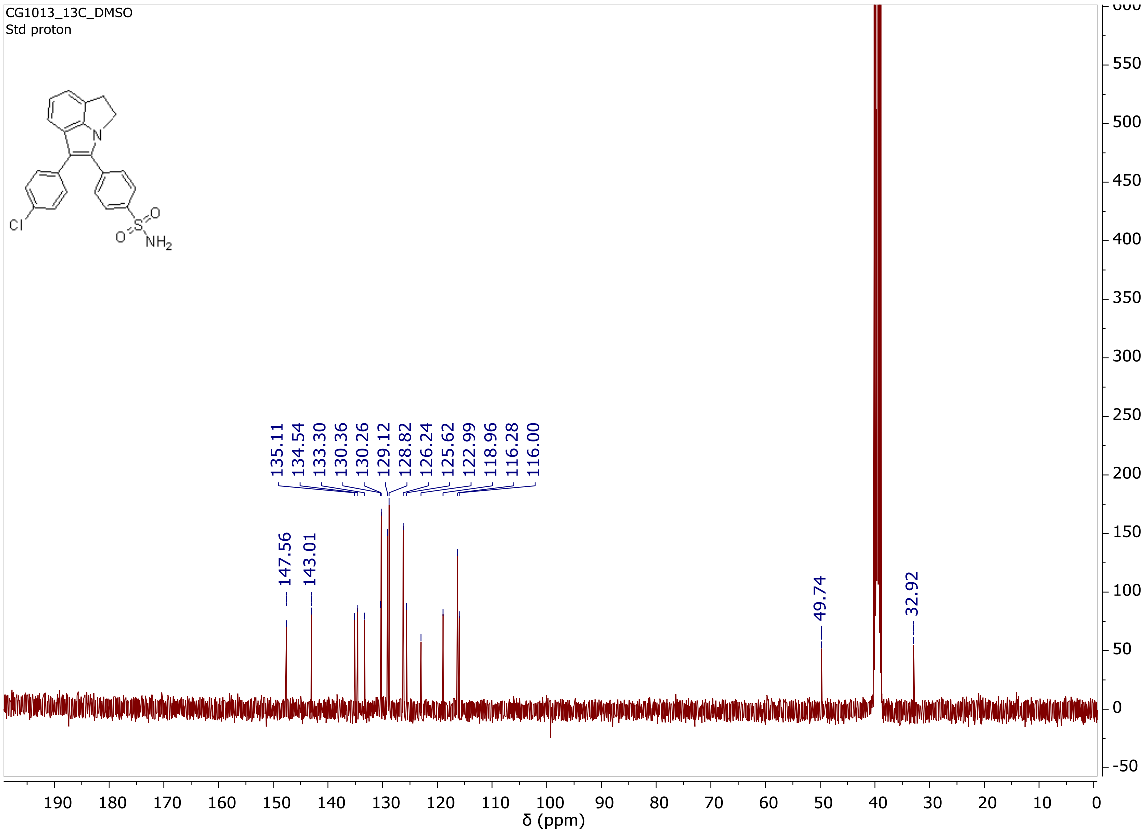


Figure S8. $^{13}$C NMR spectrum of compound **1c** in DMSO-*d*6

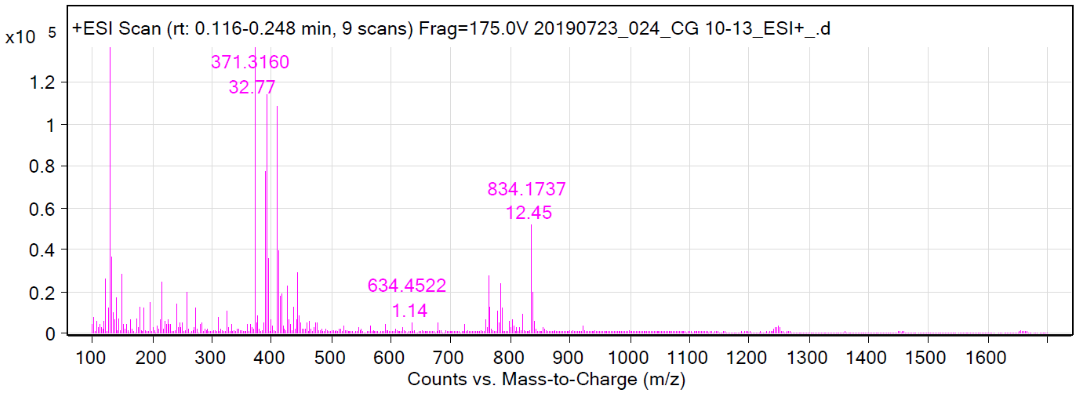


**Peak List**

| *m/z* | z | Abund |
|---|---|---|
| 130.1592 | 1 | 415979.47 |
| 131.1625 | 1 | 36817.71 |
| 371.316 | 1 | 136307.16 |
| 388.3424 | 1 | 77054.45 |
| 391.2845 | 1 | 113723.22 |
| 393.2975 | 1 | 36120.84 |
| 409.0773 | 1 | 108032.32 |
| 411.0745 | 1 | 39170.84 |
| 834.1737 | 1 | 51780 |
| 836.1723 | 1 | 42403.77 |

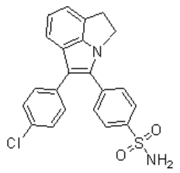


Figure S9. HRMS spectrum of compound **1c**

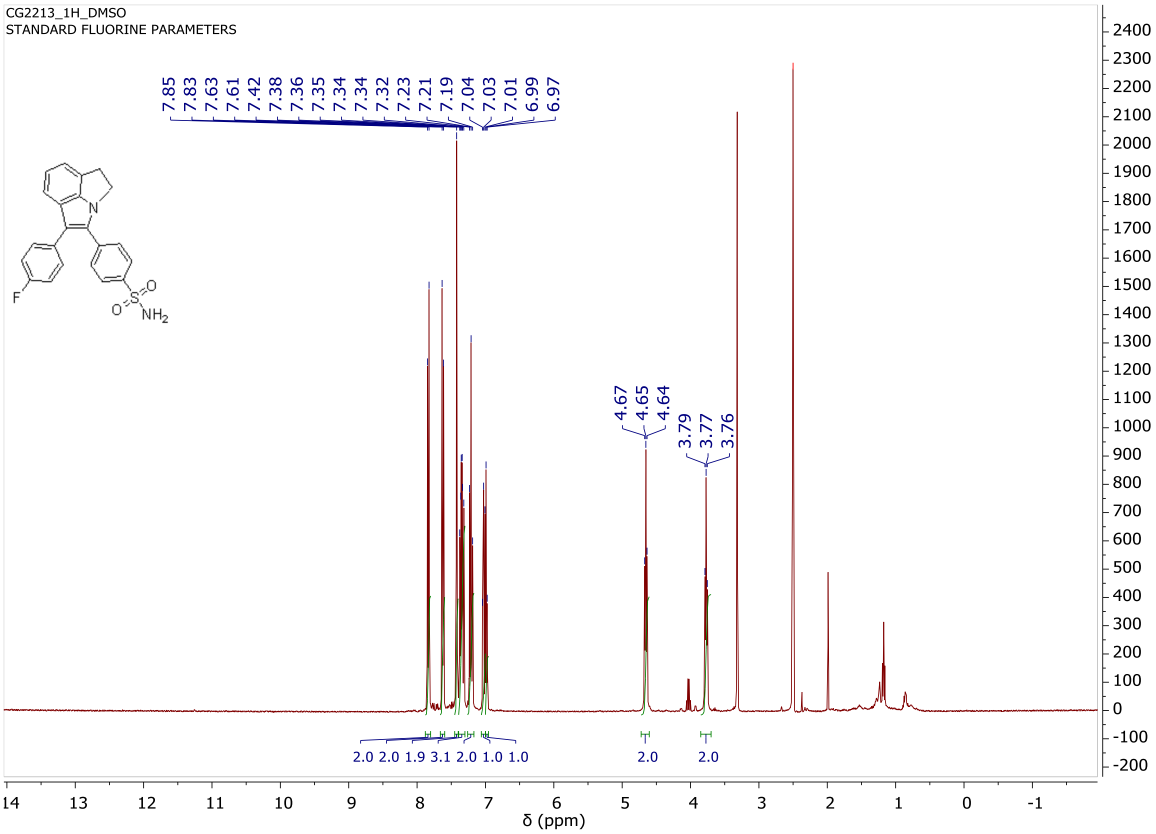


Figure S10. $^{1}$H NMR spectrum of compound **1d** in DMSO-$d_6$

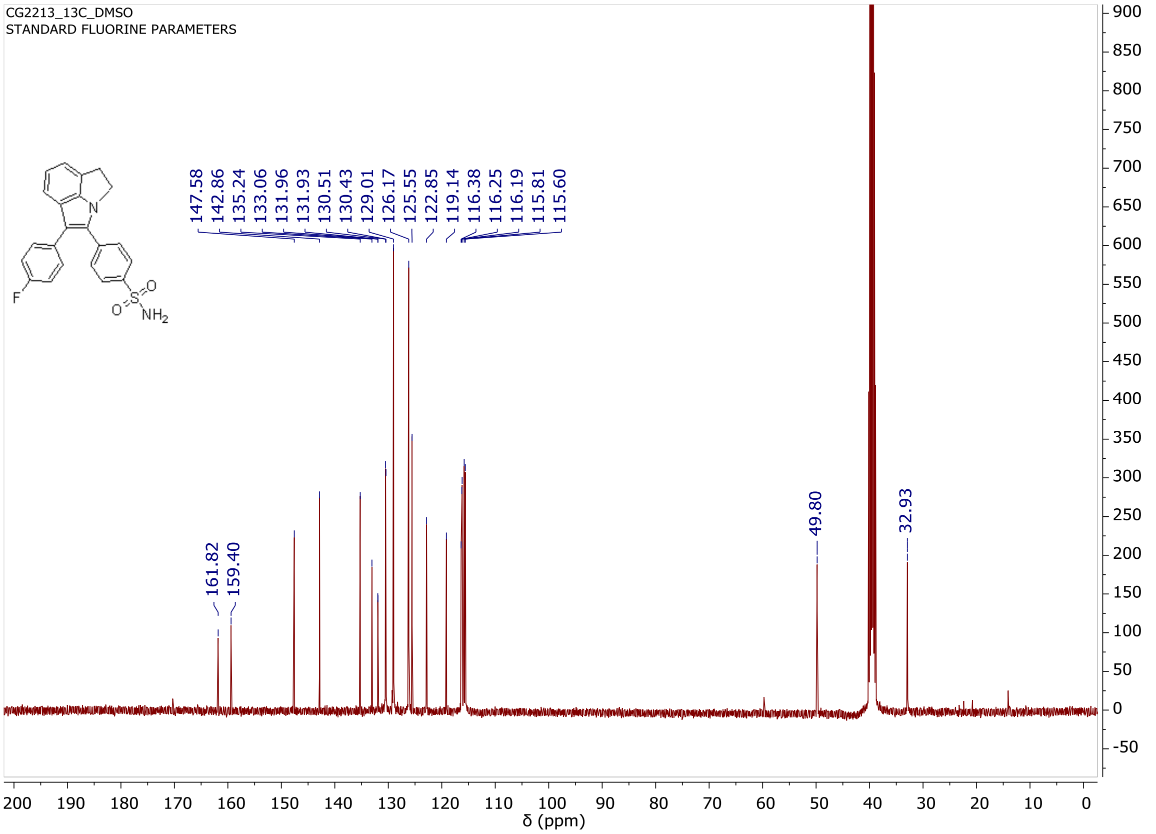


Figure S11. $^{13}$C NMR spectrum of compound **1d** in DMSO-*d*6

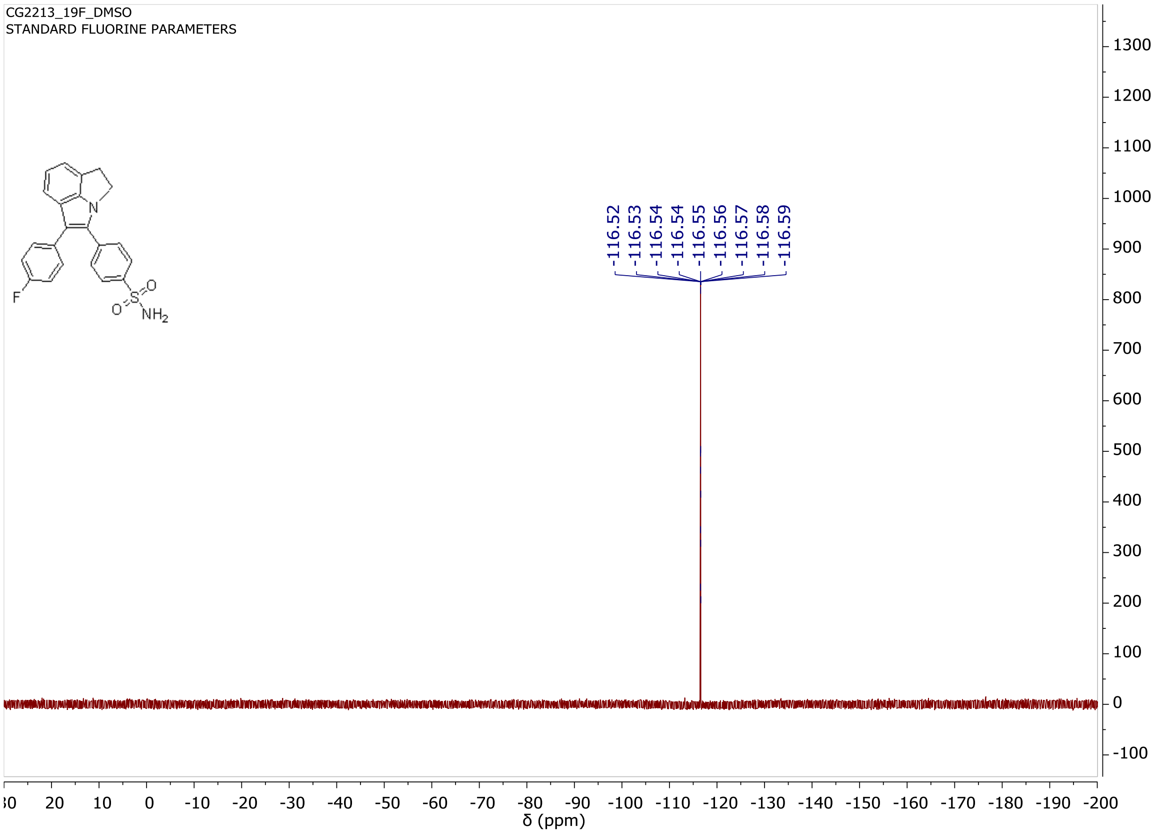


Figure S12. $^{19}$F NMR spectrum of compound **1d** in DMSO-*d*6

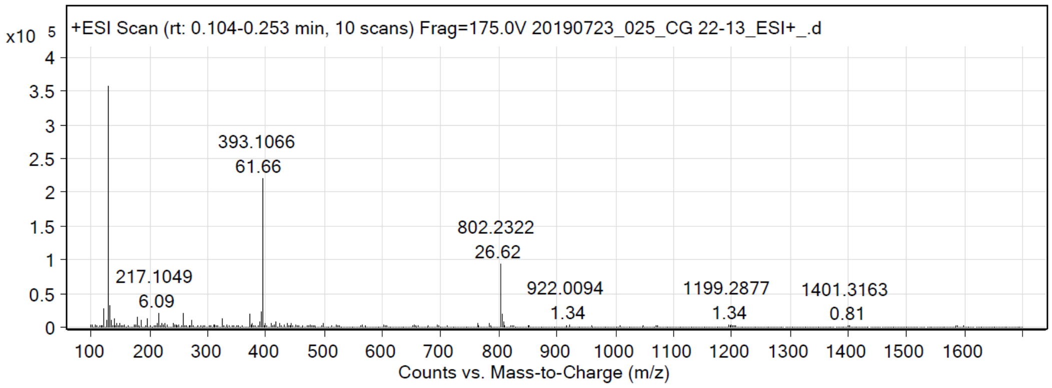


**Peak List**

| *m/z* | z | Abund |
|---|---|---|
| 121.0509 | | 26966.77 |
| 130.1591 | 1 | 357419.66 |
| 131.1624 | 1 | 31763.78 |
| 217.1049 | | 21763 |
| 391.2841 | 1 | 22836.37 |
| 392.0988 | | 21993.41 |
| 393.1066 | 1 | 220392.3 |
| 394.1097 | 1 | 53514.43 |
| 802.2322 | 1 | 95149.47 |
| 803.2352 | 1 | 46947.27 |

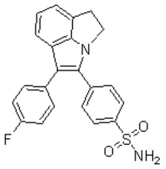

Figure S13. HRMS spectrum of compound **1d**

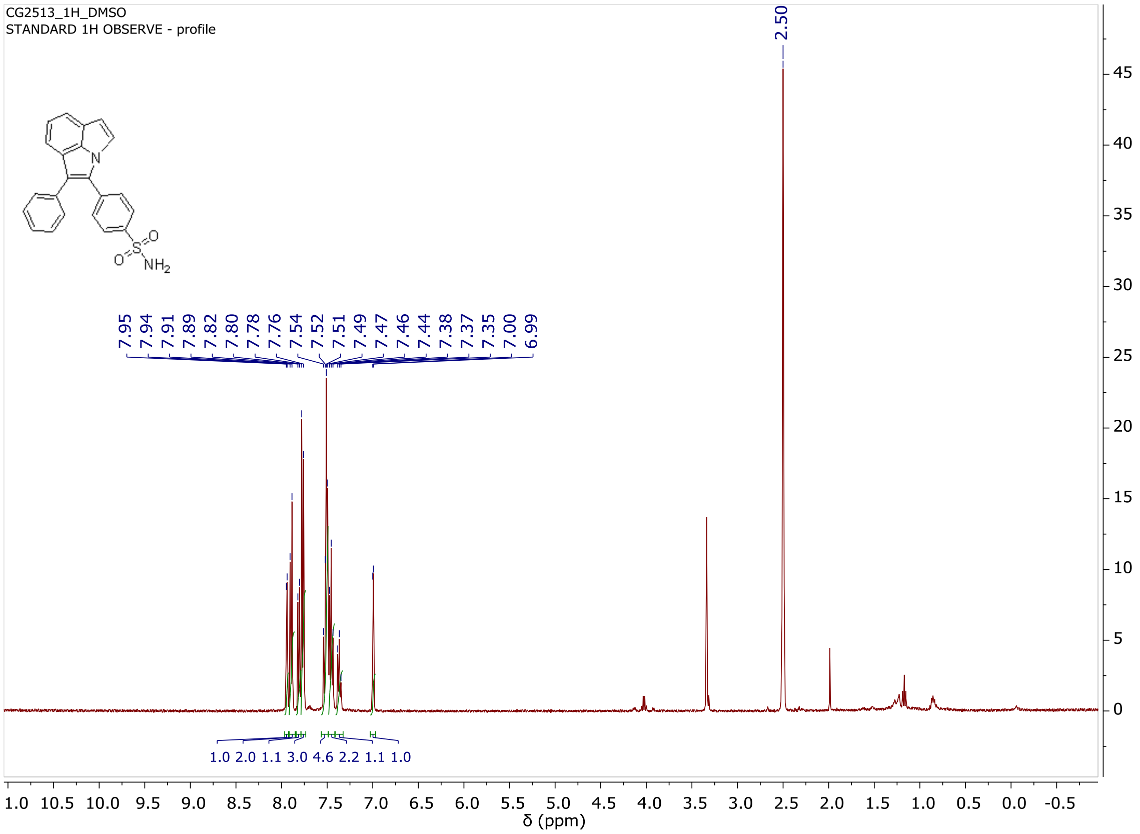


Figure S14. $^{1}H$ NMR spectrum of compound **2a** in DMSO-$d_6$

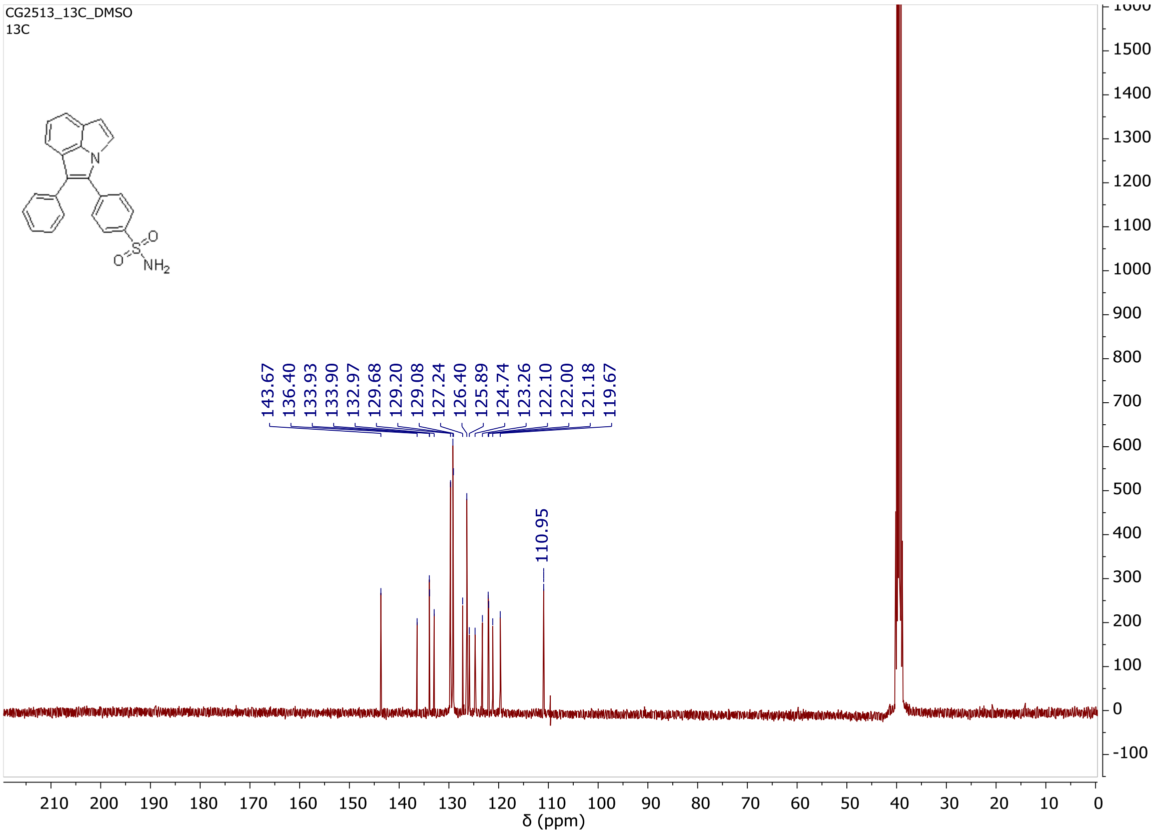


Figure S15. $^{13}$C NMR spectrum of compound **2a** in DMSO-*d*6

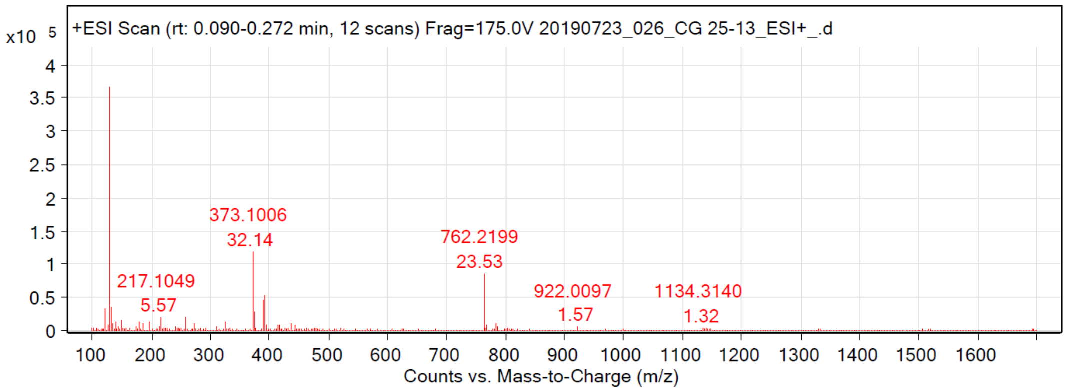


**Peak List**

| *m/z* | z | Abund |
|---|---|---|
| 121.0509 | | 32618.89 |
| 130.1591 | 1 | 366586.25 |
| 131.1625 | 1 | 34552.07 |
| 371.3158 | 1 | 32308.4 |
| 373.1006 | 1 | 117810.12 |
| 374.1037 | 1 | 29382.16 |
| 390.1271 | 1 | 46245.35 |
| 391.2844 | 1 | 52596.21 |
| 762.2199 | 1 | 86261.83 |
| 763.223 | 1 | 42440.09 |

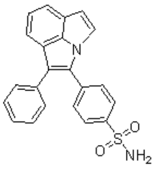

Figure S16. HRMS spectrum of compound **2a**

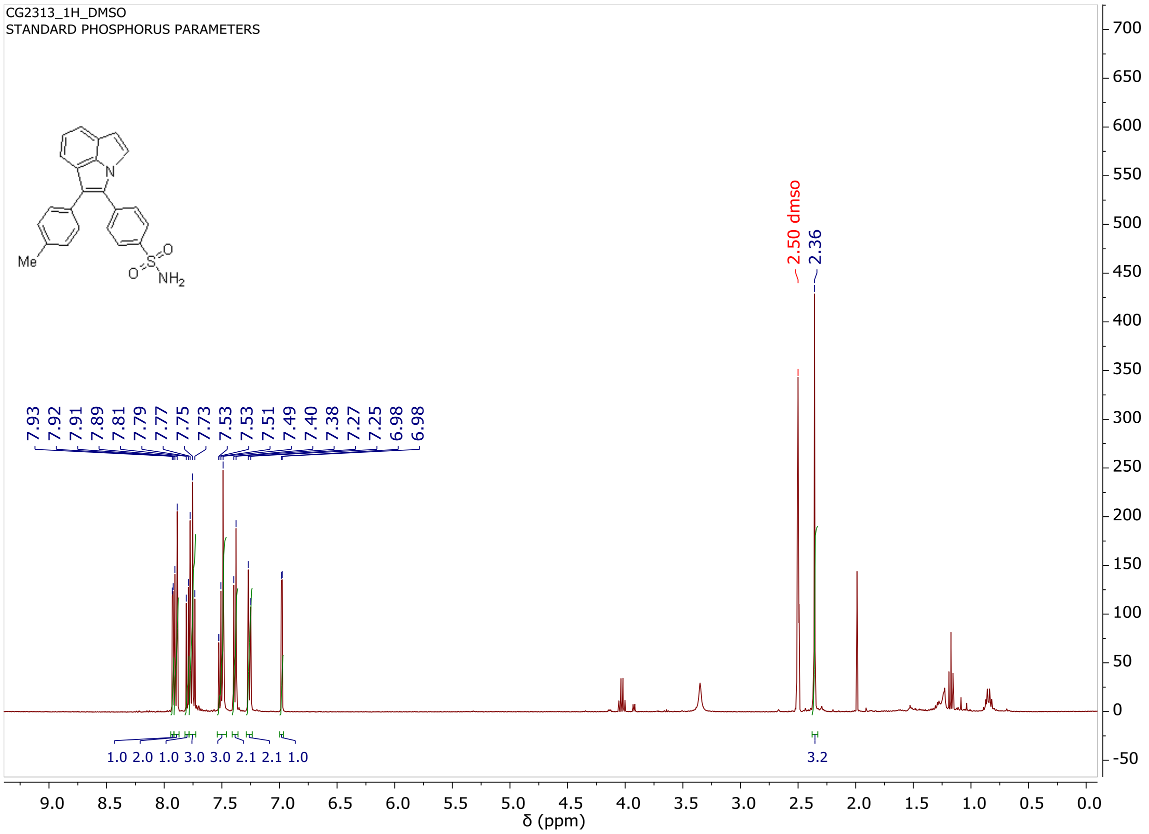


Figure S17. $^{1}$H NMR spectrum of compound **2b** in DMSO-$d_6$

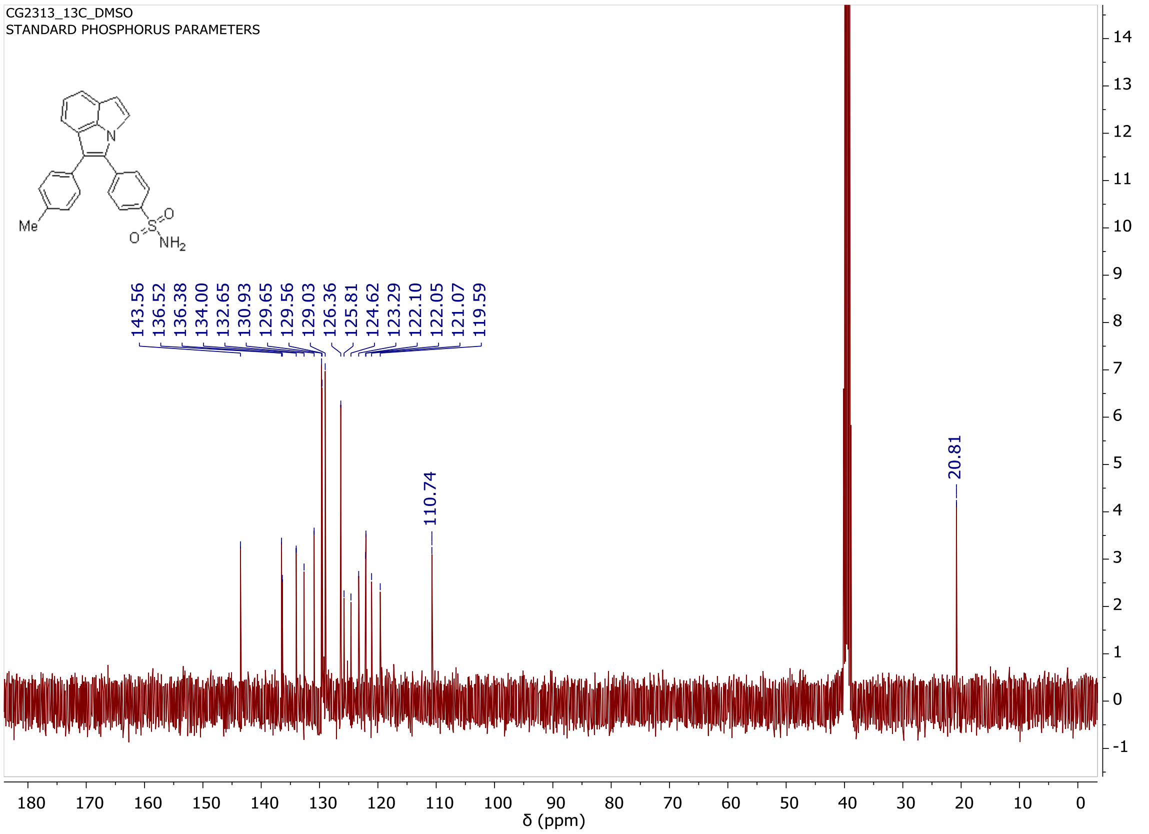


Figure S18. $^{13}$C NMR spectrum of compound **2b** in DMSO-$d_6$

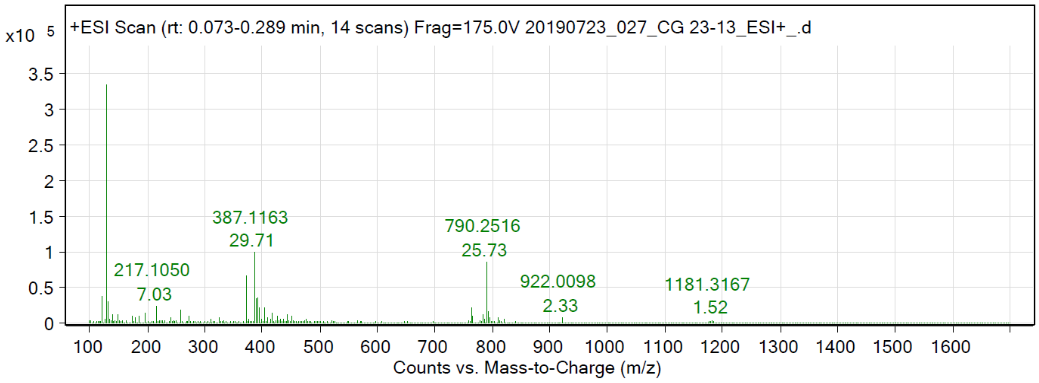


**Peak List**

| *m/z* | z | Abund |
|---|---|---|
| 121.0509 | | 38264.94 |
| 130.1591 | 1 | 334447.66 |
| 131.1625 | 1 | 30977.04 |
| 371.3158 | 1 | 65639.97 |
| 387.1163 | 1 | 99357.01 |
| 388.1195 | 1 | 24373.81 |
| 388.3423 | 1 | 34945.52 |
| 391.2845 | 1 | 35373.11 |
| 790.2516 | 1 | 86056.86 |
| 791.2546 | 1 | 44931.02 |

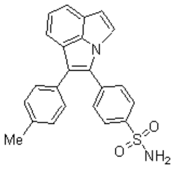


Figure S19. HRMS spectrum of compound **2b**

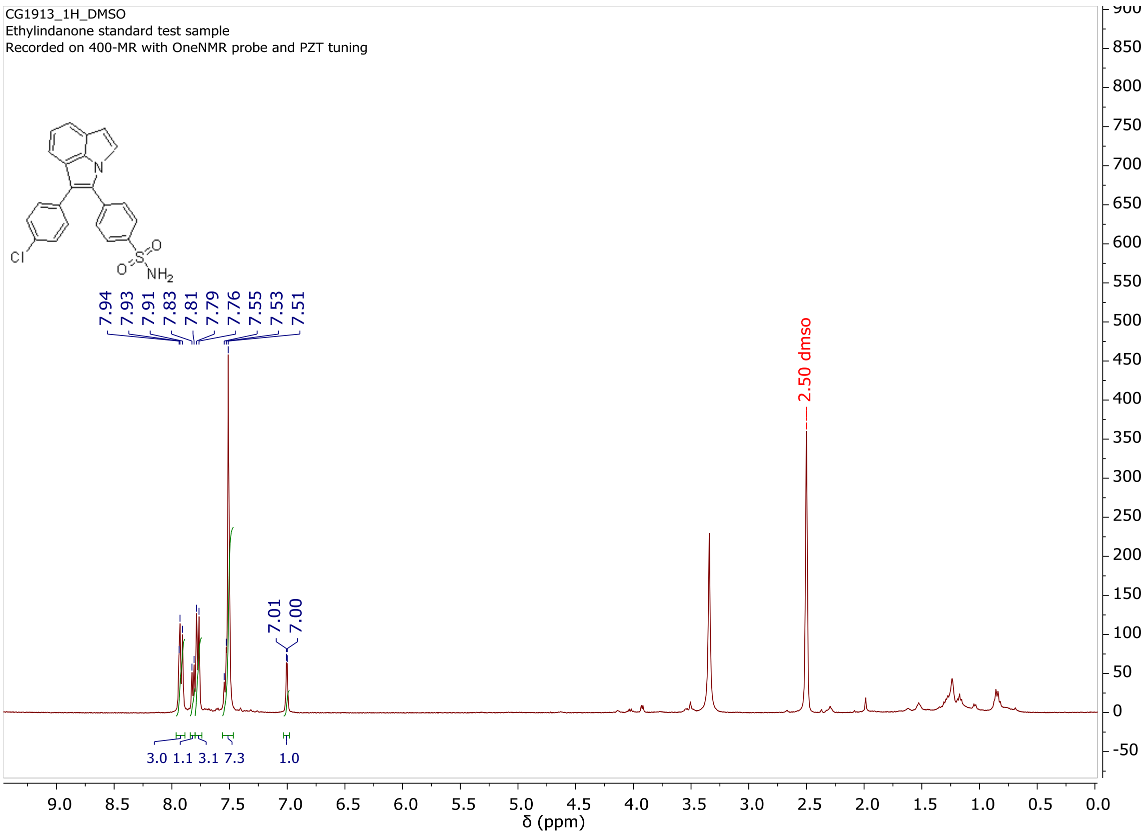


Figure S20. $^1$H NMR spectrum of compound **2c** in DMSO-$d_6$

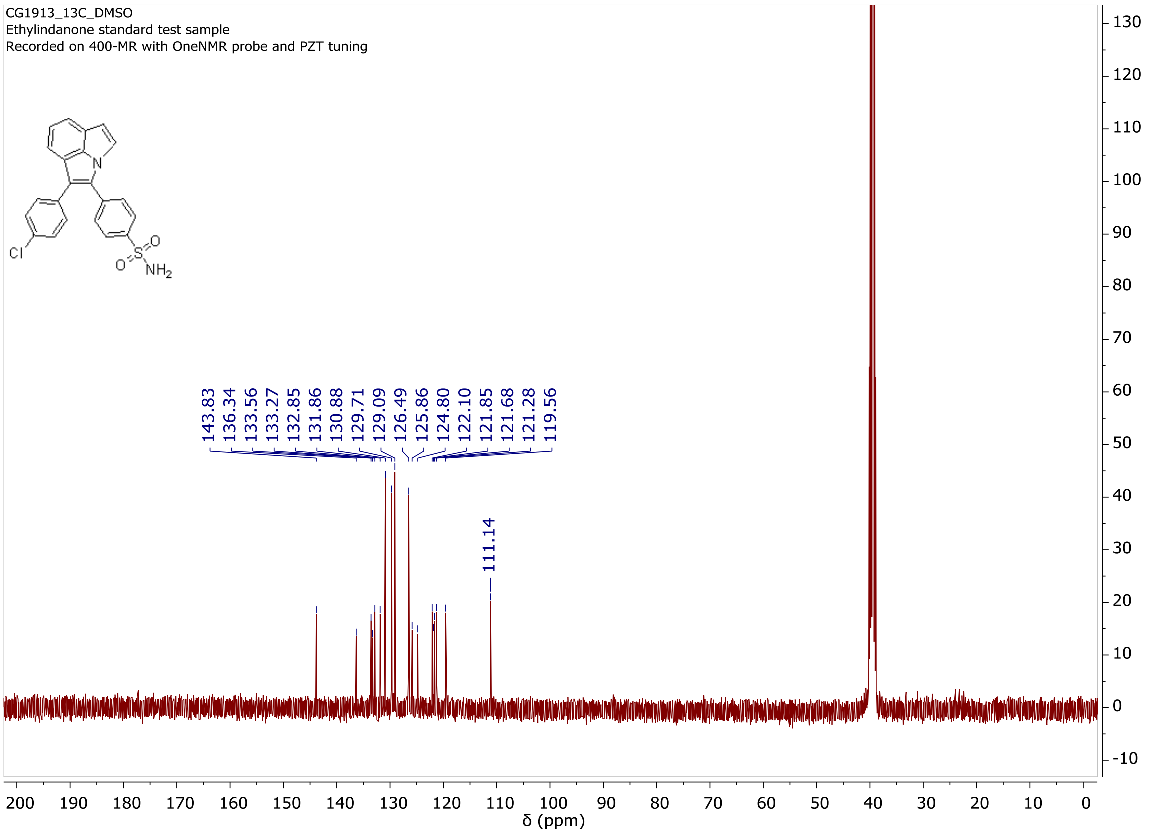


Figure S21. $^{13}$C NMR spectrum of compound **2c** in DMSO-$d_6$

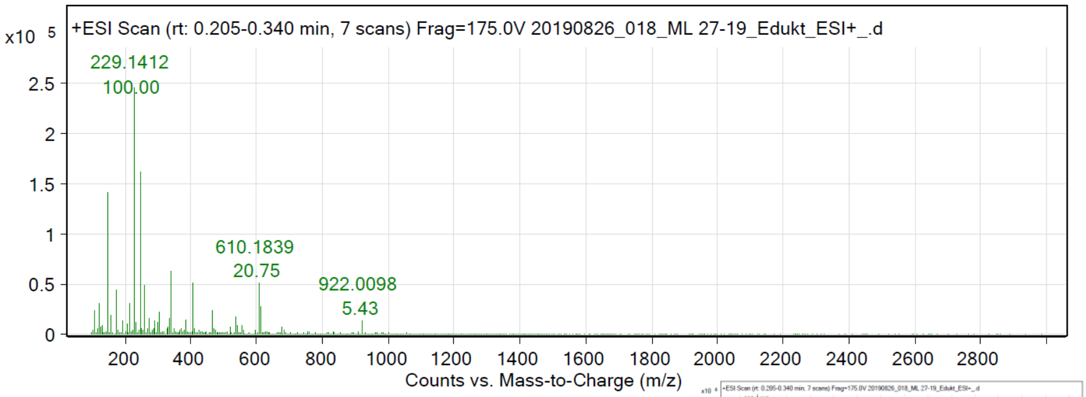


**Peak List**

| *m/z* | z | Abund |
|---|---|---|
| 121.0509 | | 30939.54 |
| 149.0234 | 1 | 141272.8 |
| 177.0547 | 1 | 44773.76 |
| 217.1048 | | 31618.14 |
| 229.1412 | 1 | 246345.47 |
| 245.0786 | 1 | 162709.48 |
| 261.1311 | 1 | 49169.85 |
| 338.3418 | 1 | 63847.57 |
| 407.0613 | 1 | 51024.46 |
| 610.1839 | 1 | 51125.37 |

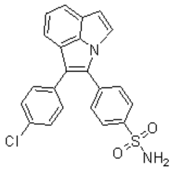

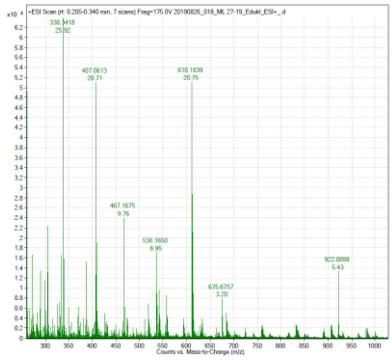

Figure S22. HRMS spectrum of compound **2c**

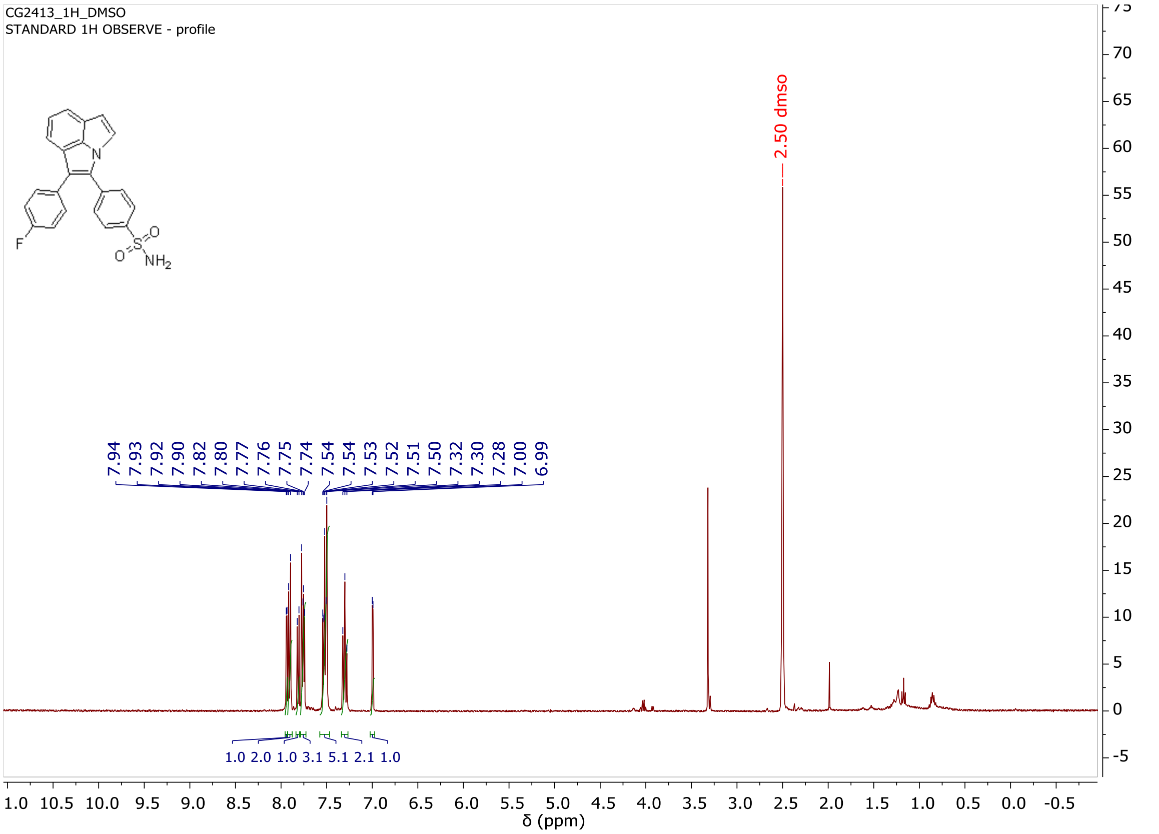


Figure S23. $^{1}$H NMR spectrum of compound **2d** in DMSO-*d*6

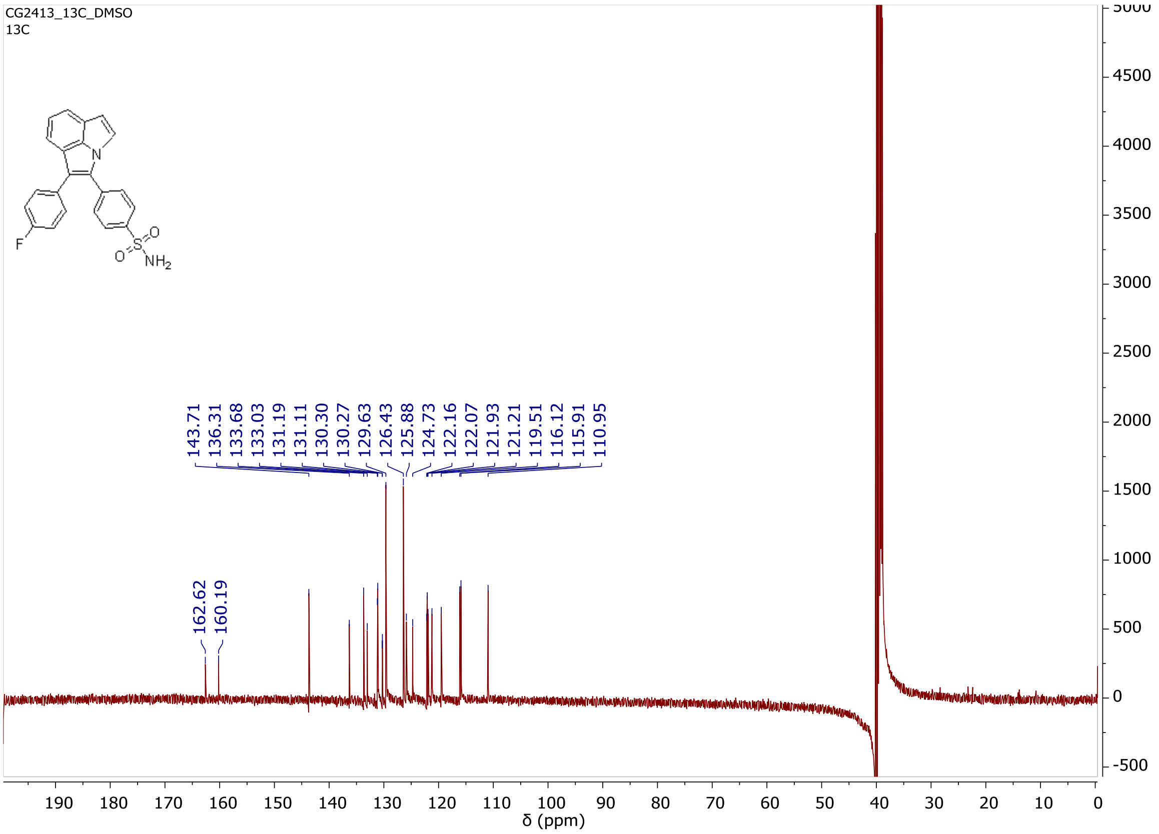


Figure S24. $^{13}$C NMR spectrum of compound **2d** in DMSO-*d*6

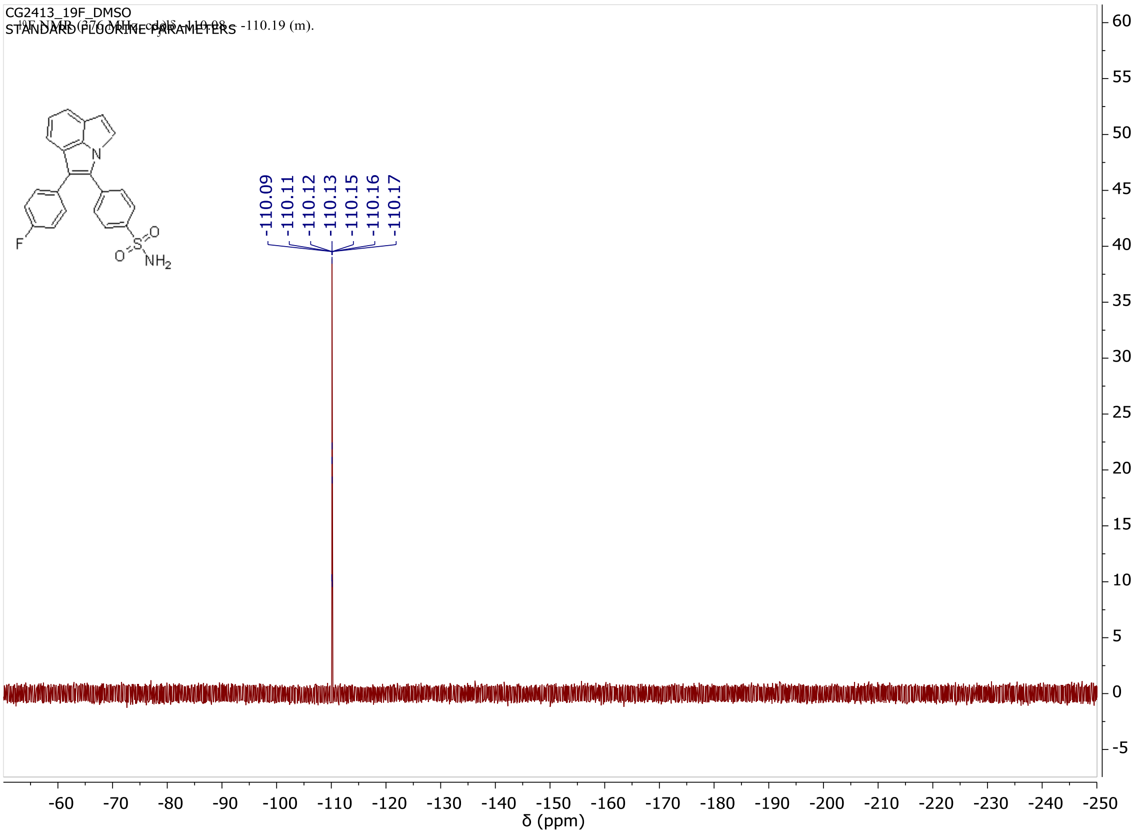


Figure S25. $^{19}$F NMR spectrum of compound **2d** in DMSO-*d*6

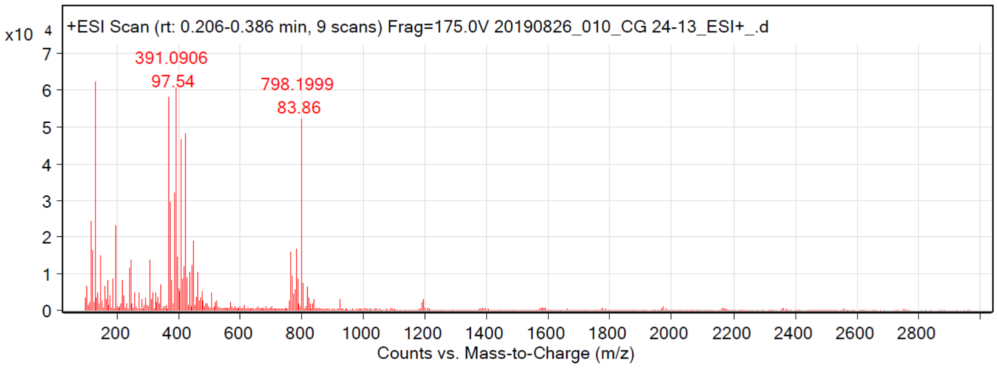


**Peak List**

| *m/z* | z | Abund |
|---|---|---|
| 130.159 | 1 | 62308.33 |
| 371.3151 | 1 | 58025.08 |
| 376.259 | 1 | 29556.12 |
| 388.3415 | 1 | 32174.27 |
| 391.0906 | 1 | 60777.33 |
| 391.2837 | 1 | 52361.62 |
| 393.2876 | 1 | 53565.48 |
| 408.117 | 1 | 46610.83 |
| 421.3167 | 1 | 48193.05 |
| 798.1999 | 1 | 52254.48 |

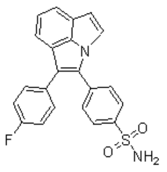

Figure S26. HRMS spectrum of compound **2d**

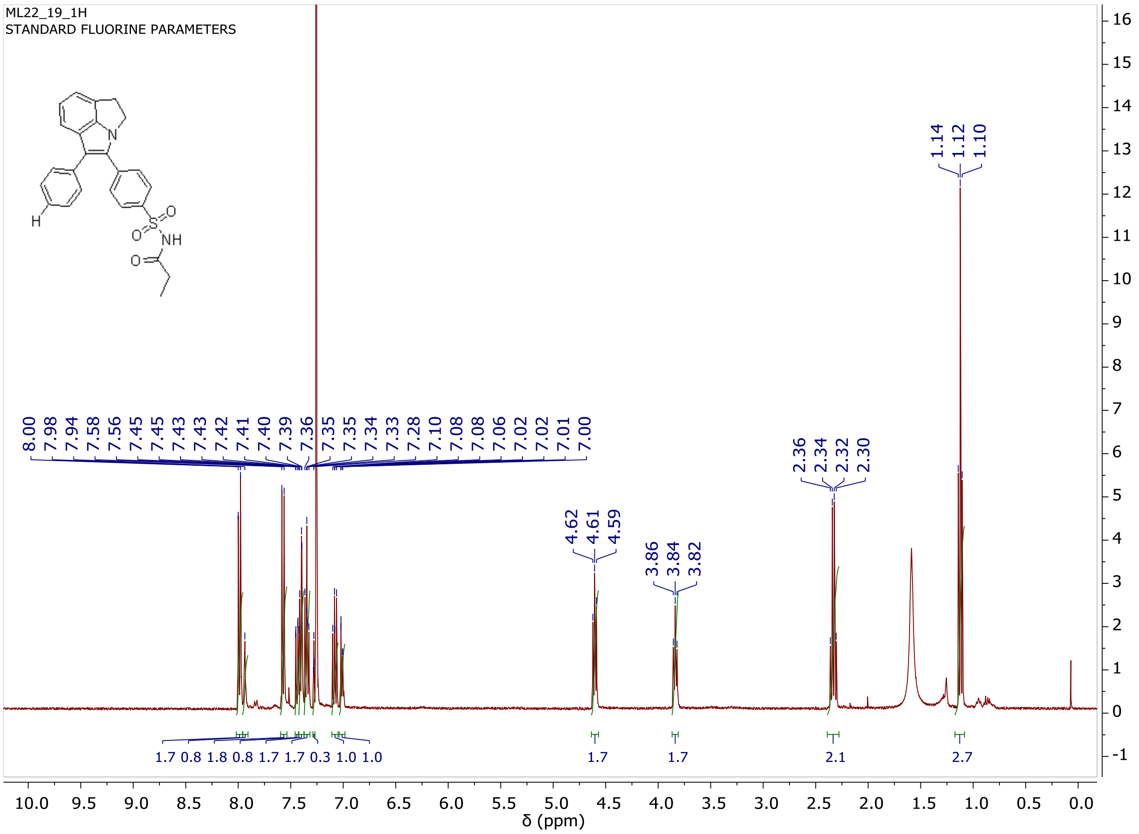


Figure S27. $^{1}$H NMR spectrum of compound **3a** in $CDCl_3$

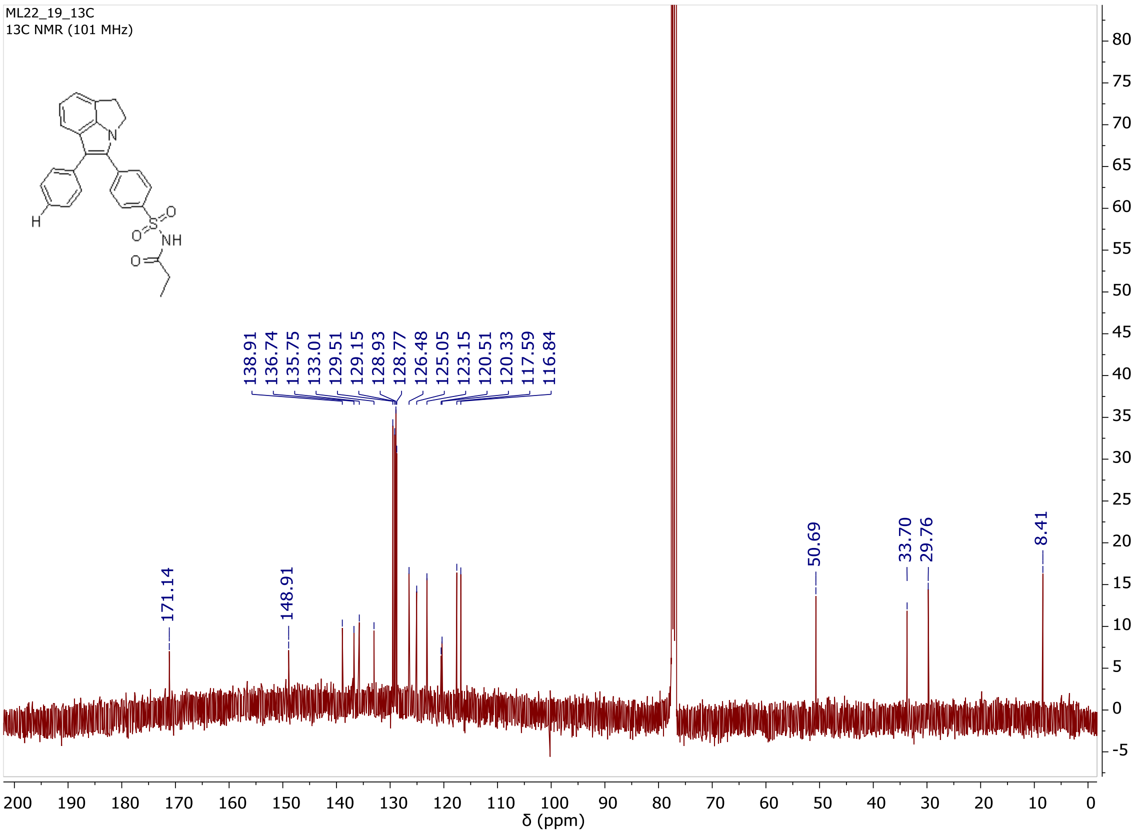


Figure S28. $^{13}$C NMR spectrum of compound **3a** in $CDCl_3$

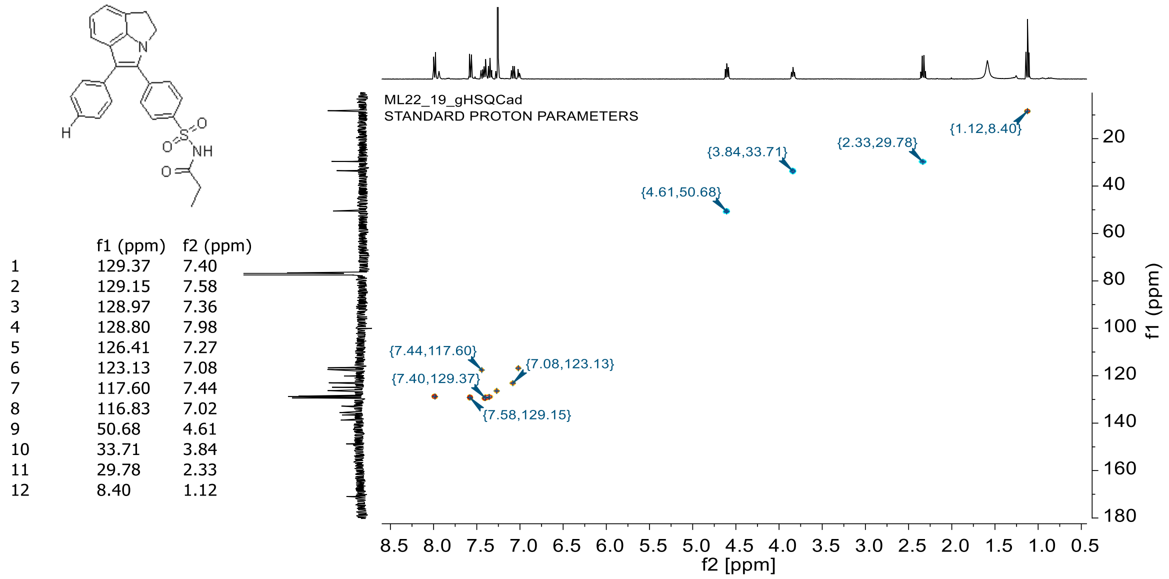


Figure S29. HSQC spectrum of compound **3a** in $CDCl_3$

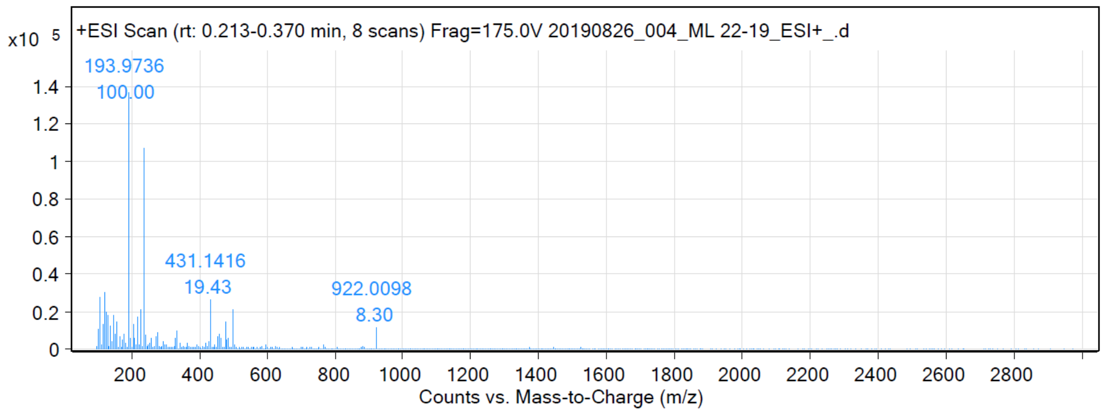


**Peak List**

| *m/z* | z | Abund |
|---|---|---|
| 106.0044 | | 27924.8 |
| 121.0509 | | 30735.99 |
| 125.9862 | | 19984.91 |
| 130.159 | | 18119.27 |
| 147.0308 | | 17996.67 |
| 193.9736 | 1 | 136905.94 |
| 229.1408 | 1 | 21039.79 |
| 235.0002 | 1 | 107047.13 |
| 431.1416 | 1 | 26596.59 |
| 500.9322 | 1 | 21391.08 |

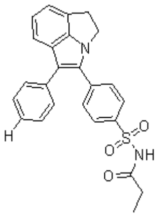

Figure S30. HRMS spectrum of compound **3a**

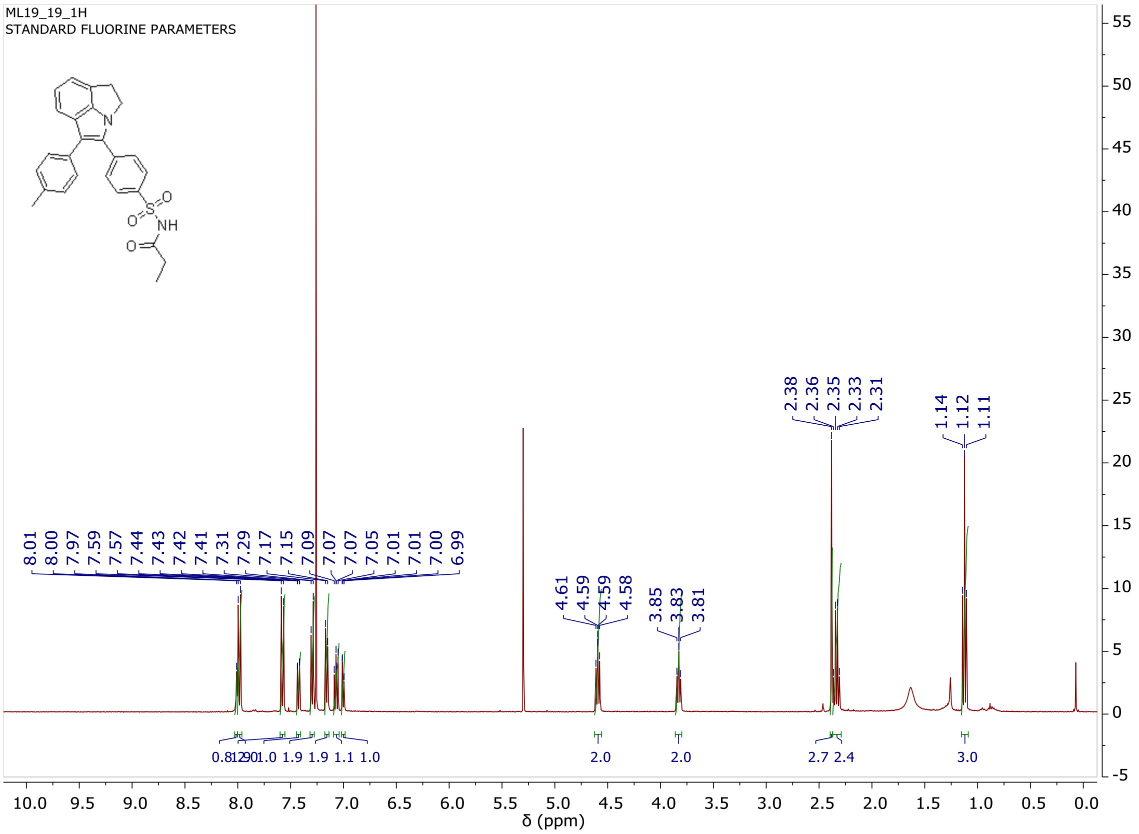


Figure S31. $^{1}$H NMR spectrum of compound **3b** in $CDCl_3$

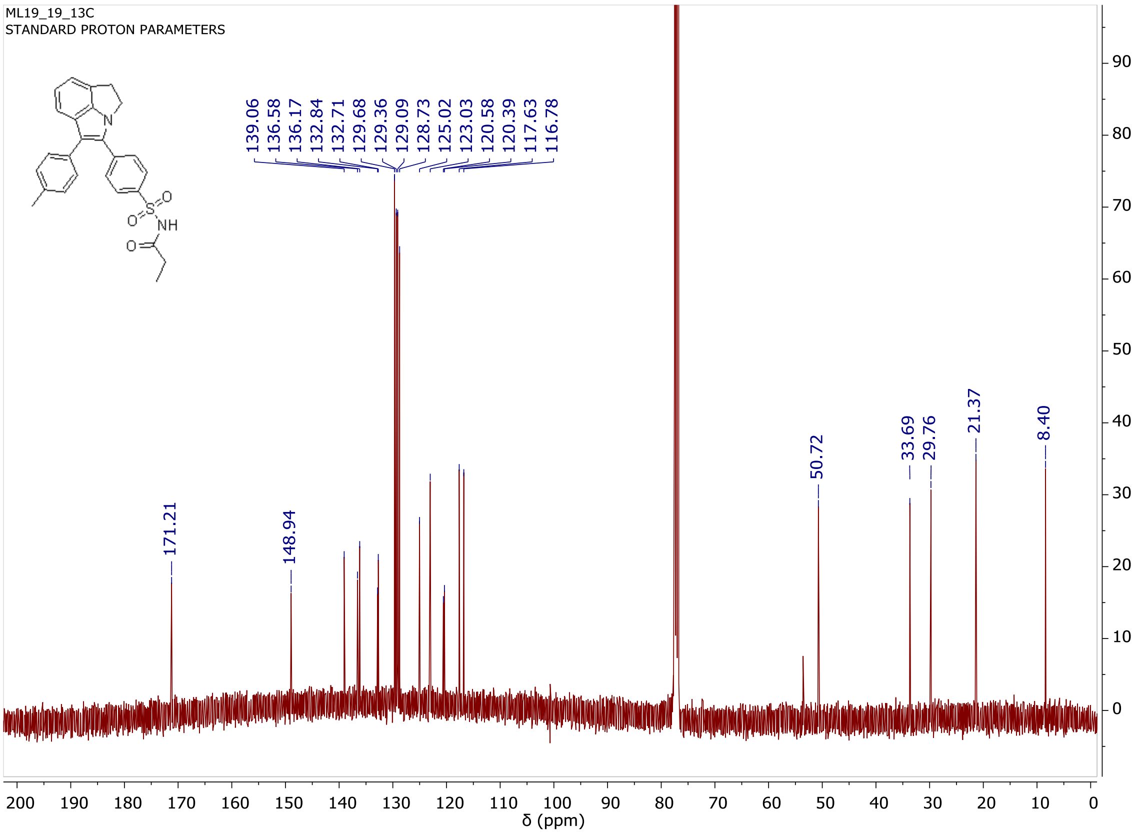


Figure S32. $^{13}$C NMR spectrum of compound **3b** in $CDCl_3$

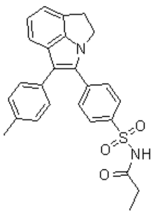

| | f1 (ppm) | f2 (ppm) |
|---|---|---|
| 1 | 129.63 | 7.16 |
| 2 | 129.34 | 7.29 |
| 3 | 129.04 | 7.57 |
| 4 | 128.72 | 7.98 |
| 5 | 123.01 | 7.07 |
| 6 | 117.61 | 7.42 |
| 7 | 116.75 | 7.00 |
| 8 | 50.72 | 4.59 |
| 9 | 33.67 | 3.82 |
| 10 | 29.74 | 2.33 |
| 11 | 21.36 | 2.38 |
| 12 | 8.40 | 1.12 |

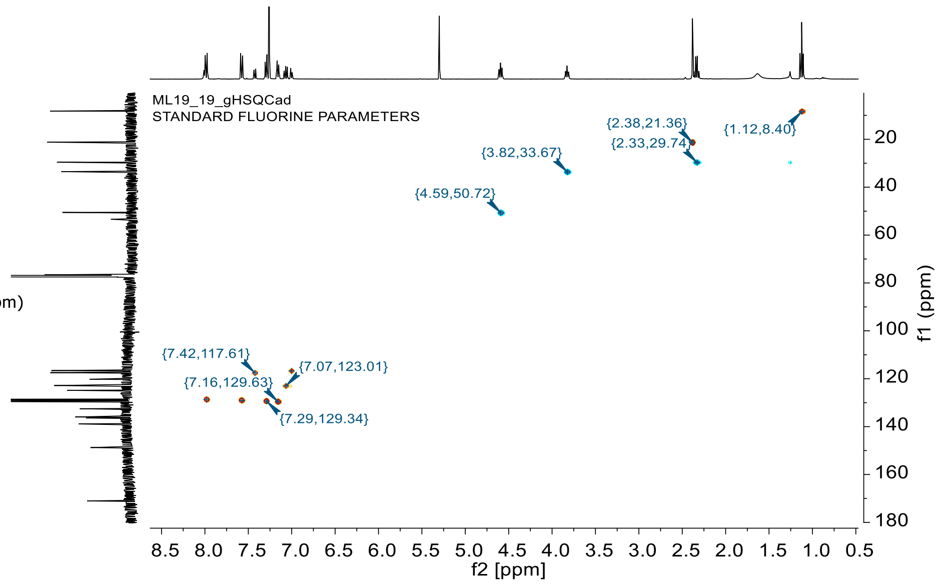


Figure S33. HSQC spectrum of compound **3b** in $CDCl_3$

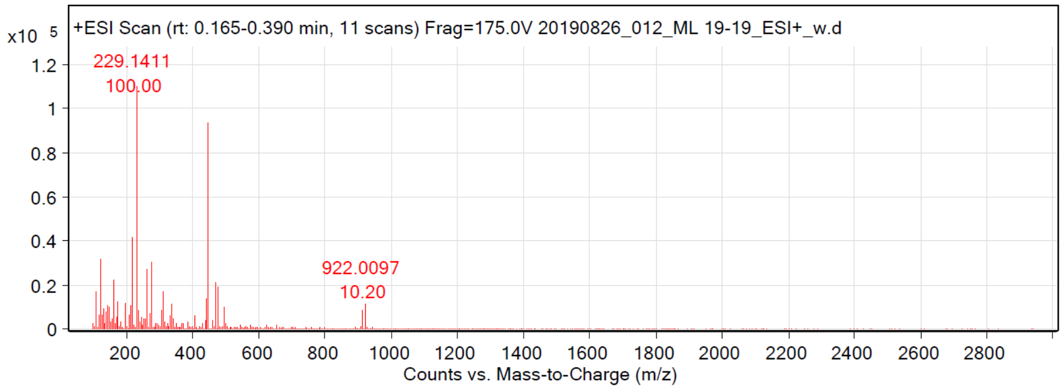


**Peak List**

| *m/z* | z | Abund |
|---|---|---|
| 121.0509 | 1 | 31624.99 |
| 158.964 | | 22749.56 |
| 217.1047 | 1 | 41589.95 |
| 229.1411 | 1 | 110270.3 |
| 261.131 | 1 | 27135.38 |
| 273.1673 | 1 | 30355.43 |
| 445.1576 | 1 | 93525.45 |
| 446.1606 | 1 | 27179.08 |
| 467.1395 | 1 | 21082.94 |
| 477.1475 | 1 | 19456.43 |

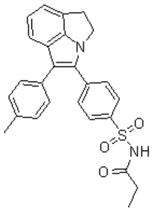

Figure S34. HRMS spectrum of compound **3b**

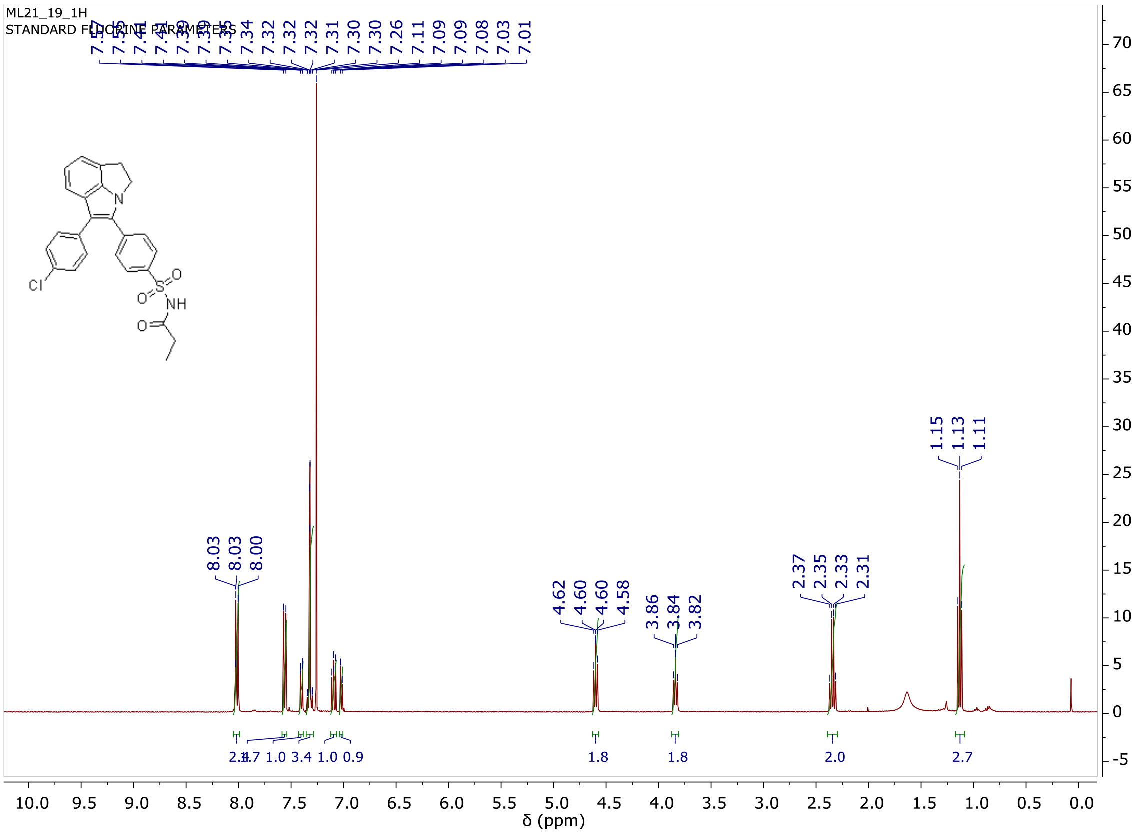


Figure S35. $^{1}$H NMR spectrum of compound **3c** in $CDCl_3$

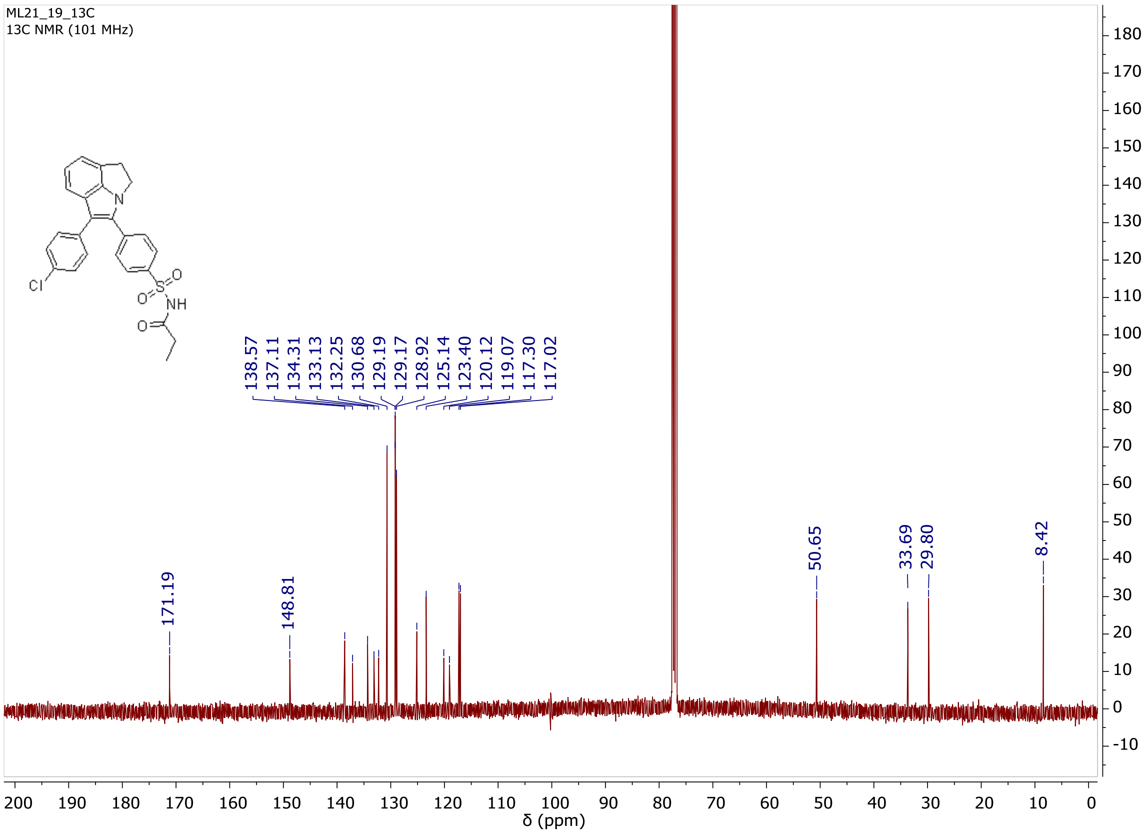


Figure S36. $^{13}$C NMR spectrum of compound **3c** in $CDCl_3$

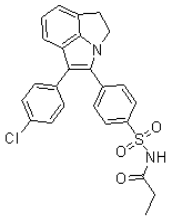


| | f1 (ppm) | f2 (ppm) |
|---|---|---|
| 1 | 130.65 | 7.33 |
| 2 | 129.22 | 7.33 |
| 3 | 129.19 | 7.56 |
| 4 | 128.92 | 8.01 |
| 5 | 123.38 | 7.09 |
| 6 | 117.31 | 7.40 |
| 7 | 117.00 | 7.03 |
| 8 | 50.65 | 4.60 |
| 9 | 33.70 | 3.84 |
| 10 | 29.80 | 2.34 |
| 11 | 8.43 | 1.13 |

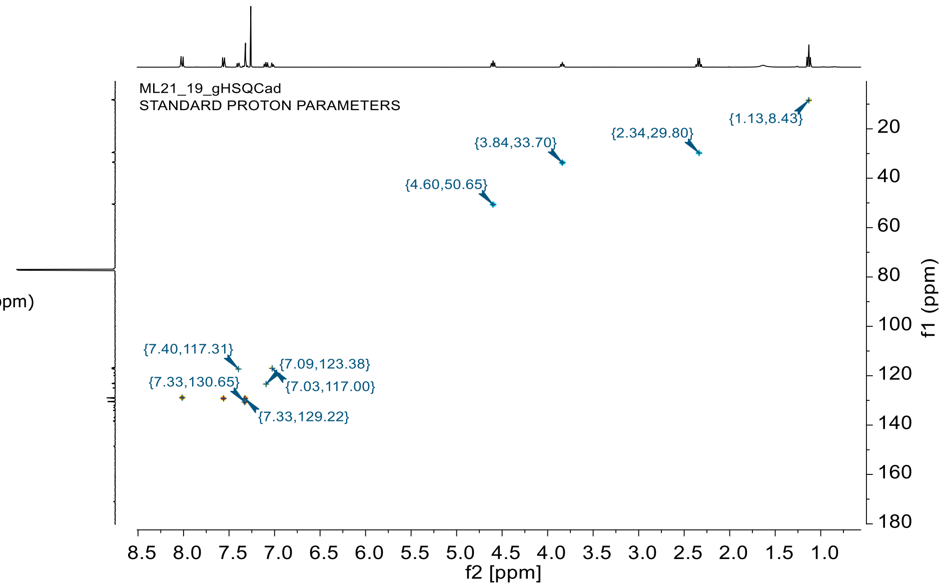


Figure S37. HSQC spectrum of compound **3c** in $CDCl_3$

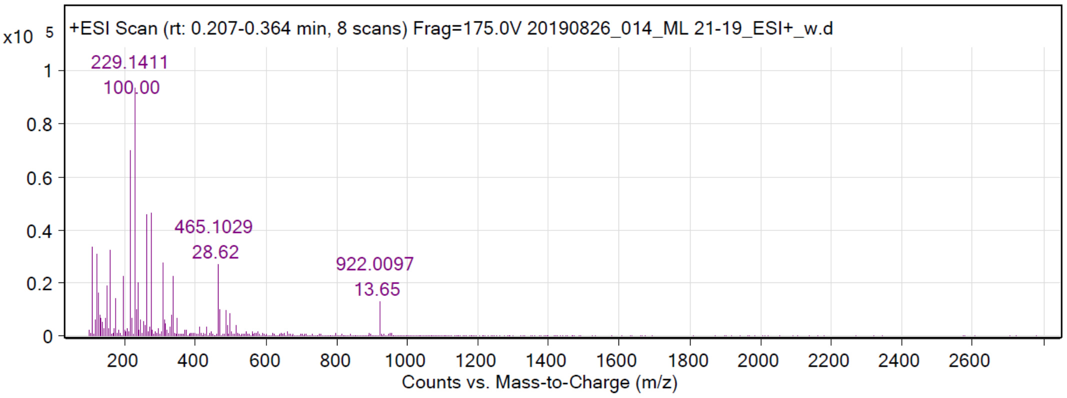


**Peak List**

| *m/z* | z | Abund |
|---|---|---|
| 106.0044 | | 33744.67 |
| 121.0509 | | 30595.82 |
| 158.964 | | 32376.59 |
| 217.1047 | 1 | 70016.06 |
| 229.1411 | 1 | 93579.58 |
| 261.131 | 1 | 45712.89 |
| 273.1672 | 1 | 46580.42 |
| 305.1571 | 1 | 27645.86 |
| 335.0042 | 1 | 22481 |
| 465.1029 | 1 | 26784.51 |

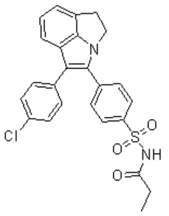


Figure S38. HRMS spectrum of compound **3c**

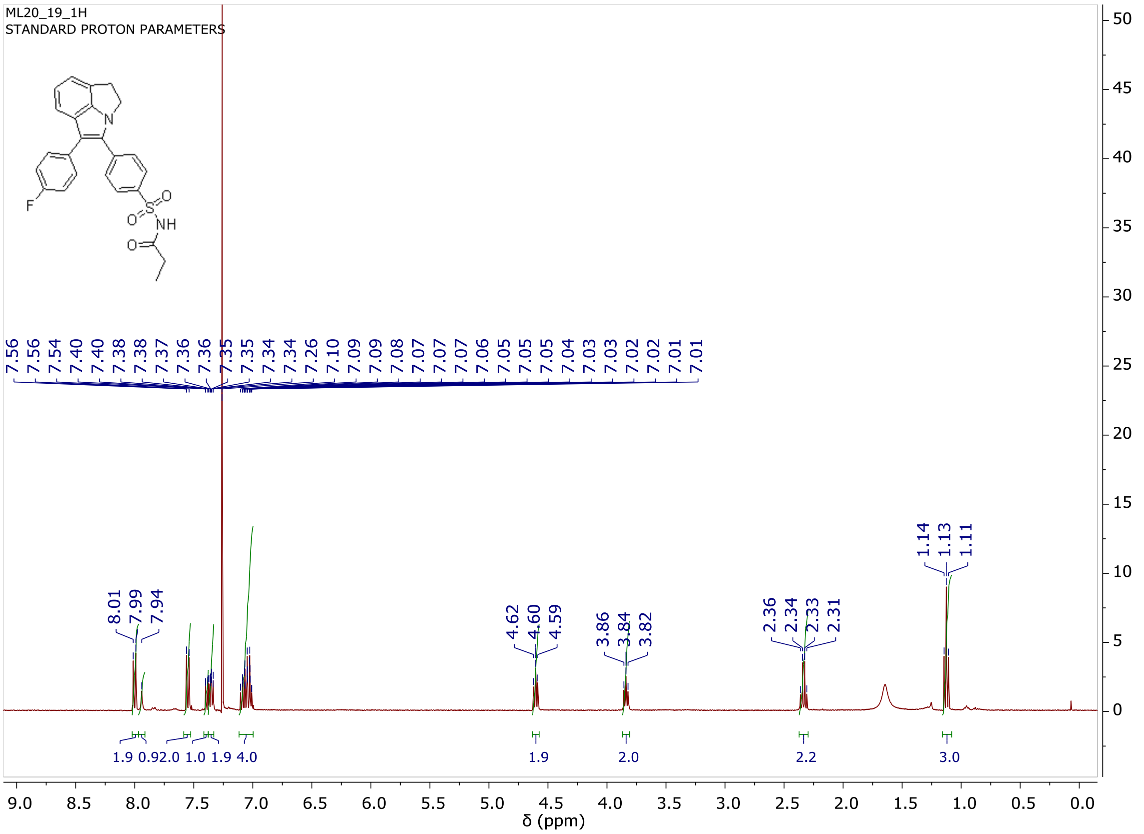


Figure S39. $^{1}$H NMR spectrum of compound **3d** in $CDCl_3$

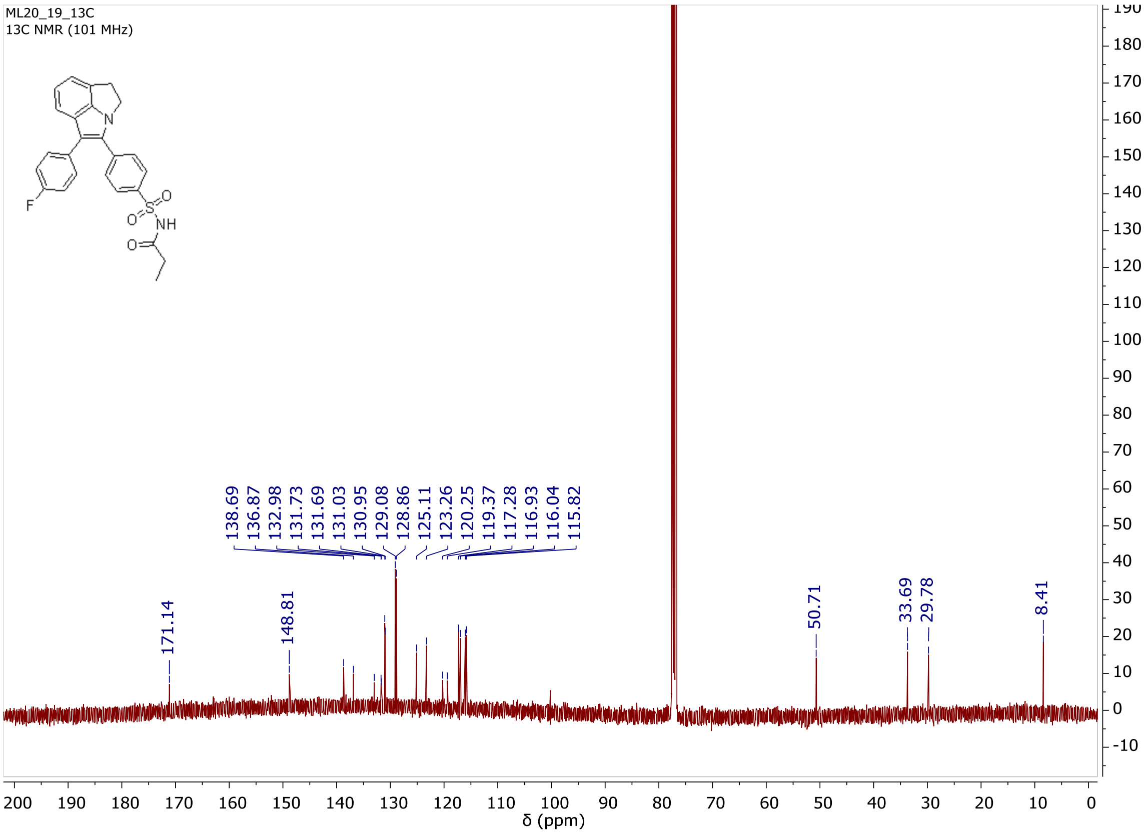


Figure S40. $^{13}$C NMR spectrum of compound **3d** in $CDCl_3$

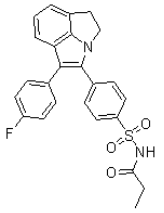

| | f1 (ppm) | f2 (ppm) |
|---|---|---|
| 1 | 131.01 | 7.35 |
| 2 | 129.10 | 7.55 |
| 3 | 128.89 | 7.99 |
| 4 | 123.30 | 7.08 |
| 5 | 117.29 | 7.38 |
| 6 | 115.95 | 7.04 |
| 7 | 50.71 | 4.60 |
| 8 | 33.70 | 3.83 |
| 9 | 29.78 | 2.33 |
| 10 | 8.41 | 1.12 |

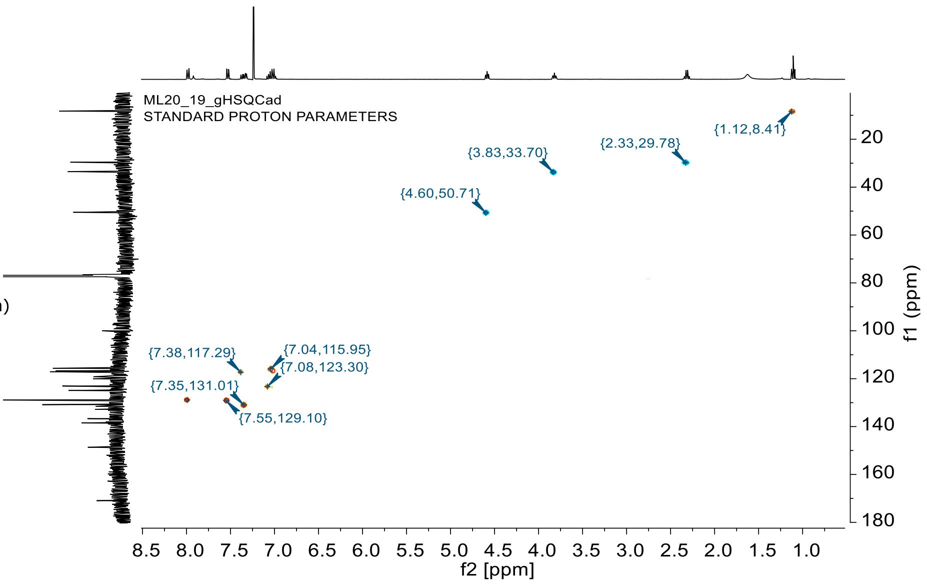


Figure S41. HSQC spectrum of compound **3d** in $CDCl_3$

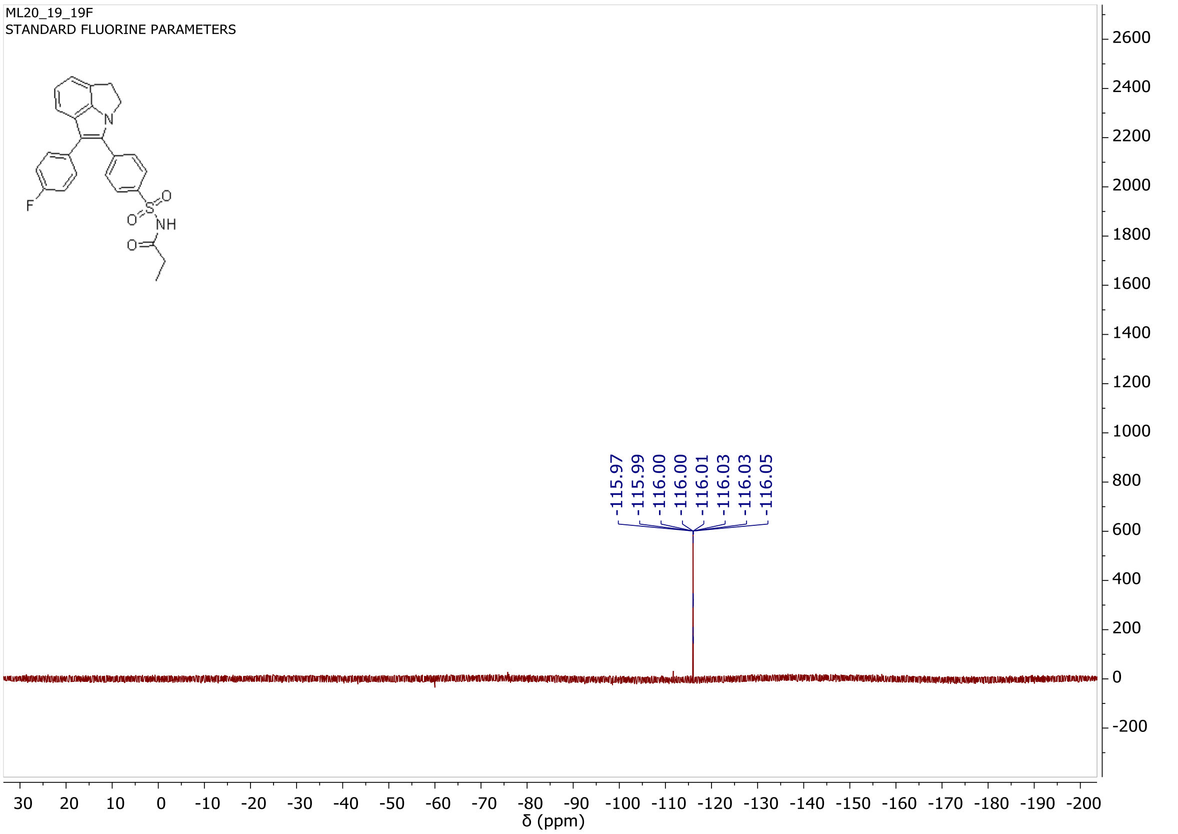


Figure S42. $^{19}F$ NMR spectrum of compound **3d** in $CDCl_3$

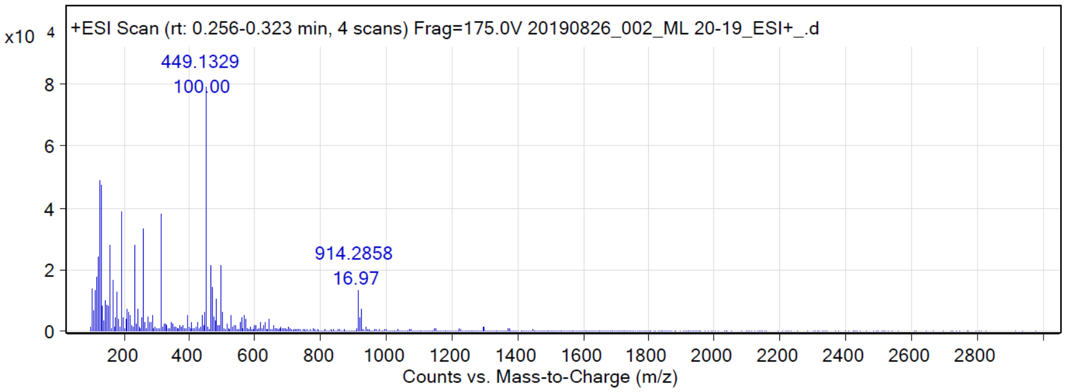


**Peak List**

| m/z | z | Abund |
|---|---|---|
| 121.0509 | | 24270.76 |
| 125.9863 | | 48989.84 |
| 130.159 | 1 | 47452.37 |
| 158.0029 | | 28017.24 |
| 193.9736 | | 39126.17 |
| 235.0004 | | 28141.35 |
| 258.0929 | 1 | 33404.07 |
| 312.1186 | 1 | 37871.18 |
| 449.1329 | 1 | 79288.77 |
| 497.1177 | 1 | 21572.33 |

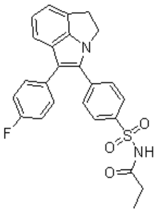

Figure S43. HRMS spectrum of compound **3d**

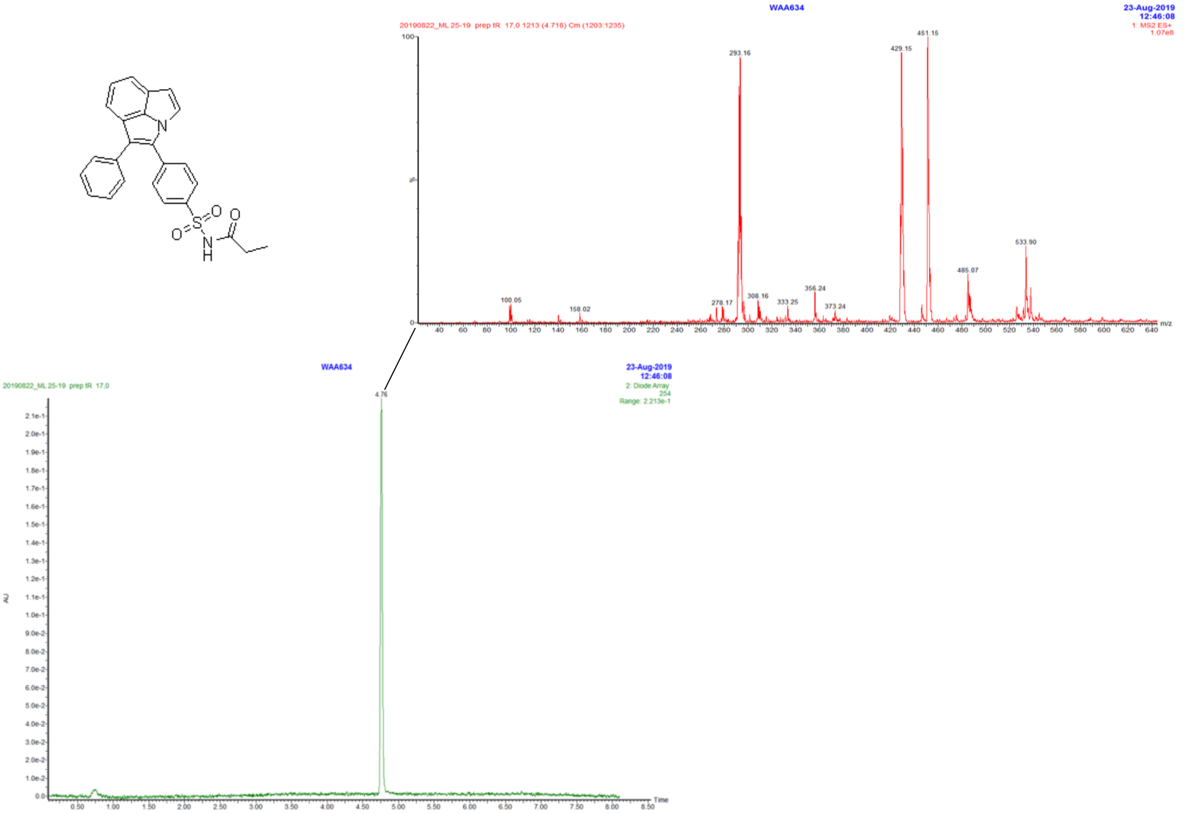


Figure S44. UPLC-MS chromatogram and spectrum of compound **4a**

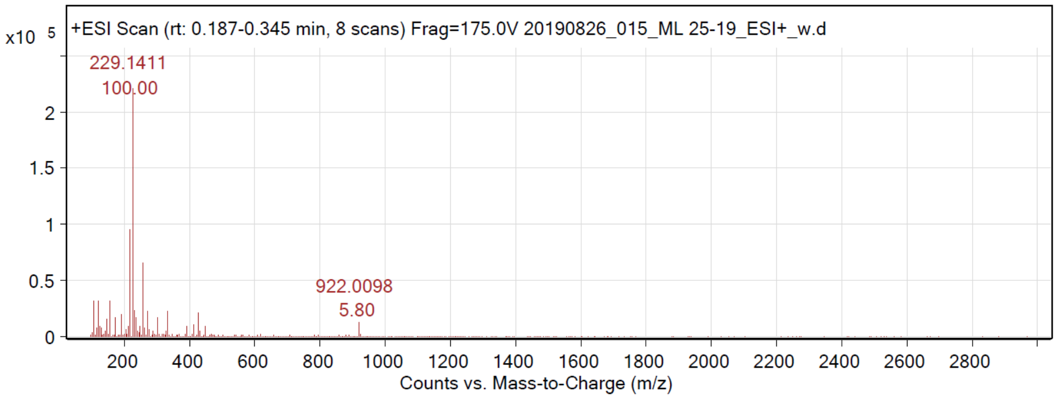


**Peak List**

| *m/z* | z | Abund |
|---|---|---|
| 106.0044 | | 31731.66 |
| 121.0509 | 1 | 32320.74 |
| 158.964 | | 31221.07 |
| 217.1047 | 1 | 95616.11 |
| 229.1411 | 1 | 220864.17 |
| 230.1444 | 1 | 23971.44 |
| 261.131 | 1 | 66121.91 |
| 273.1671 | 1 | 22571.94 |
| 335.0041 | 1 | 22657.35 |
| 429.1262 | 1 | 20862.11 |

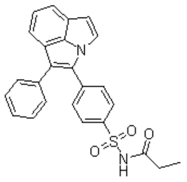

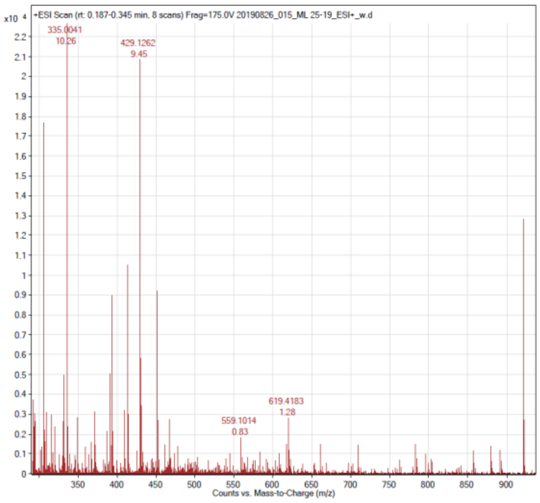


Figure S45. HRMS spectrum of compound **4a**

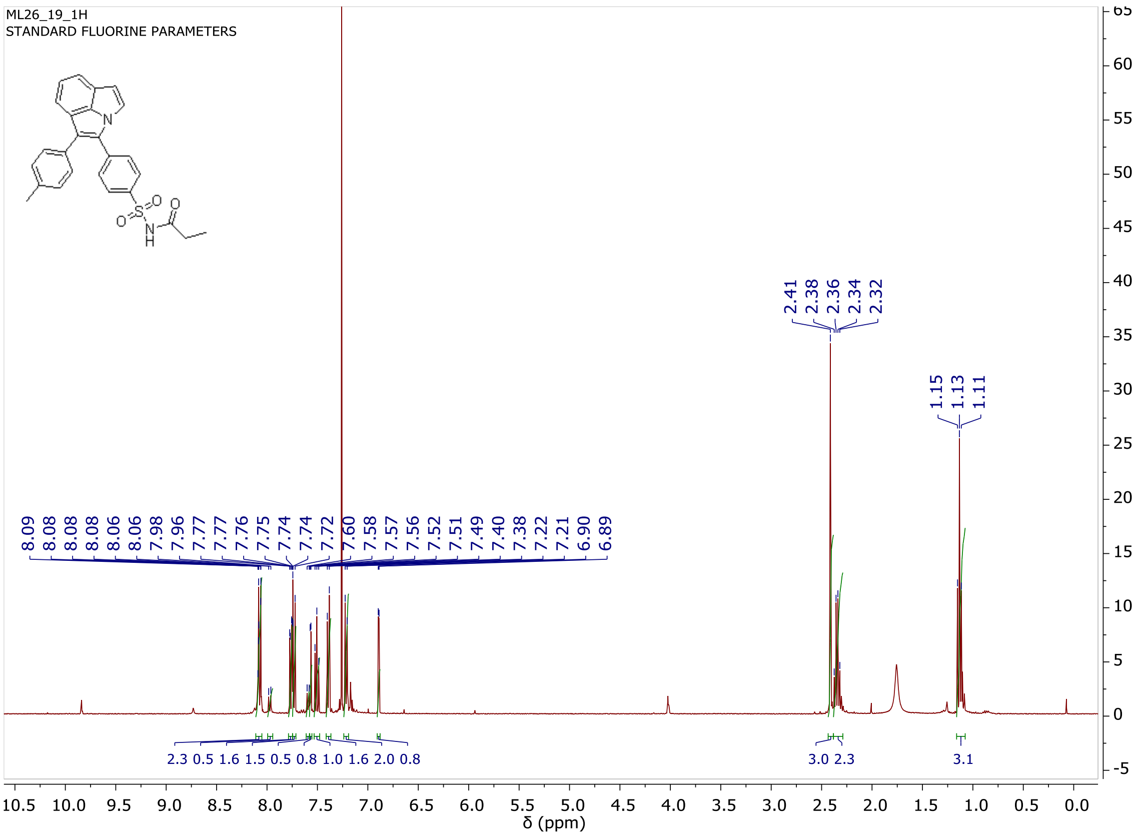


Figure S46. $^{1}$H NMR spectrum of compound **4b** in $CDCl_3$

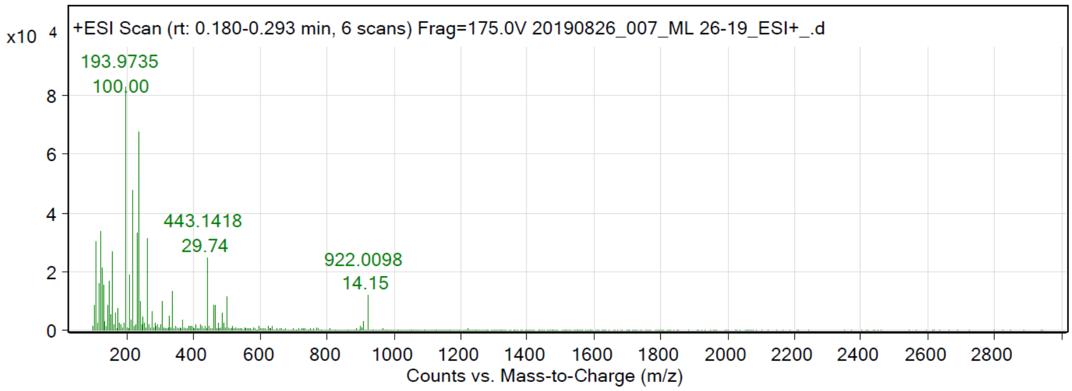


**Peak List**

| *m/z* | z | Abund |
|---|---|---|
| 106.0043 | | 30195.72 |
| 121.0509 | 1 | 33797.78 |
| 125.9862 | | 21587.03 |
| 158.0028 | | 27033.29 |
| 193.9735 | 1 | 83212.66 |
| 217.1046 | 1 | 47688.3 |
| 229.1409 | 1 | 33173.68 |
| 235.0003 | 1 | 67918.05 |
| 261.1309 | 1 | 31159.07 |
| 443.1418 | 1 | 24750.84 |

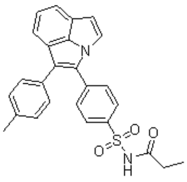


Figure S47. HRMS spectrum of compound **4b**

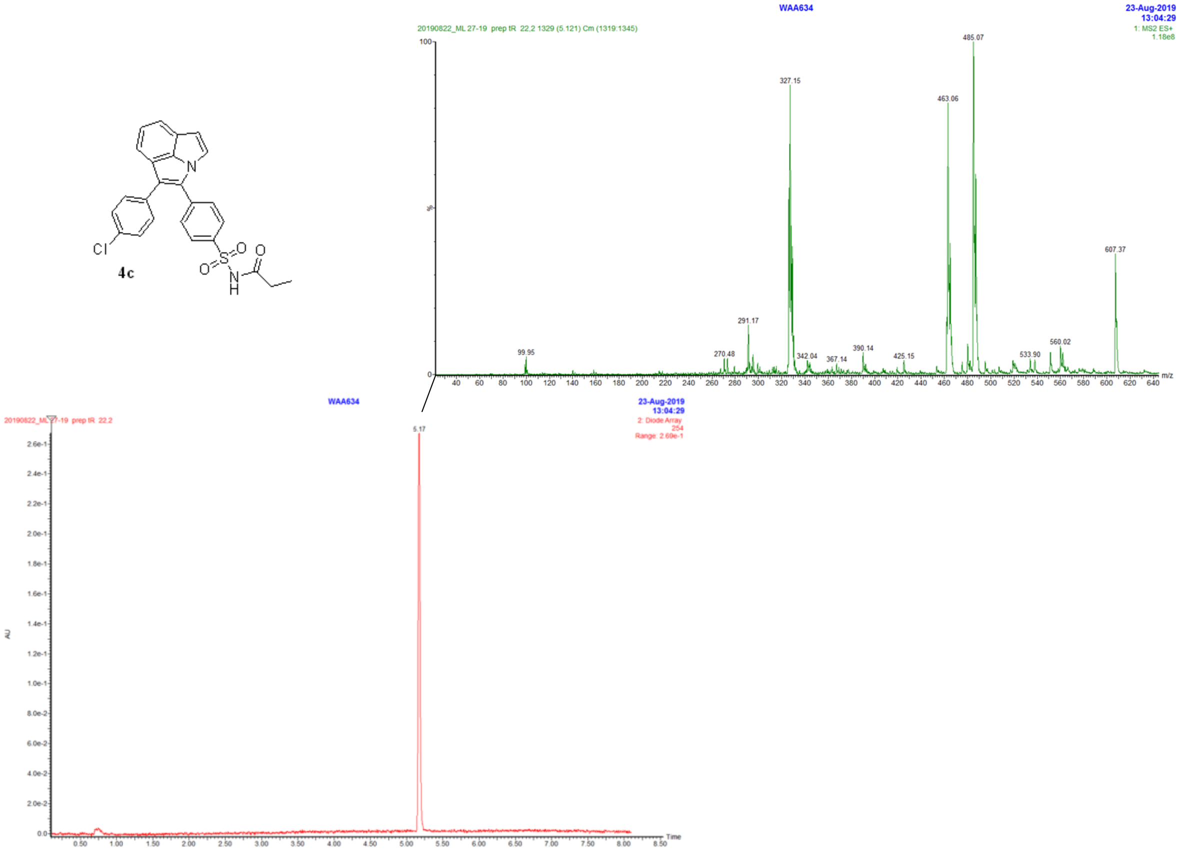


Figure S48. UPLC-MS chromatogram and spectrum of compound **4c**

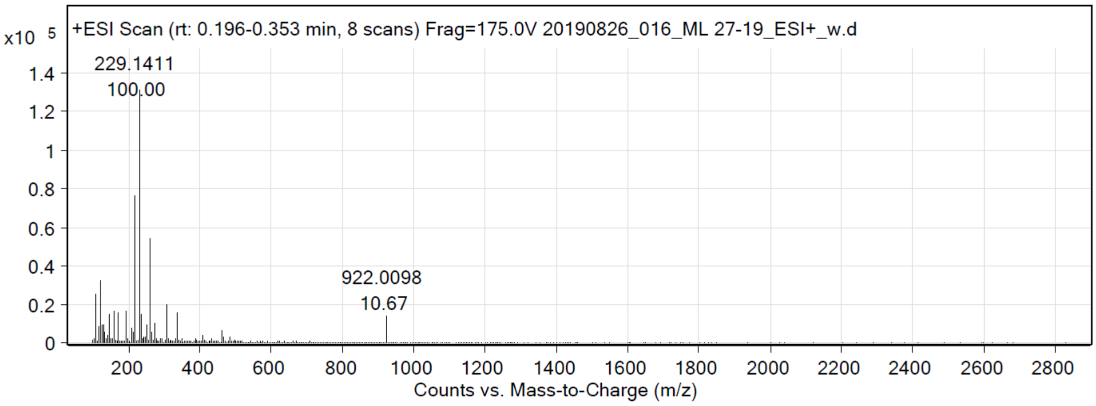


**Peak List**

| *m/z* | z | Abund |
|---|---|---|
| 106.0044 | | 25123.54 |
| 121.0509 | 1 | 32327.33 |
| 158.9638 | | 16616.79 |
| 173.0783 | | 15839.42 |
| 193.9735 | | 16413.49 |
| 217.1047 | 1 | 76198.04 |
| 229.1411 | 1 | 131354.94 |
| 261.1309 | 1 | 54065.89 |
| 305.1571 | 1 | 20059.2 |
| 335.004 | 1 | 15615.05 |

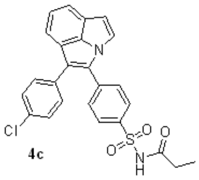


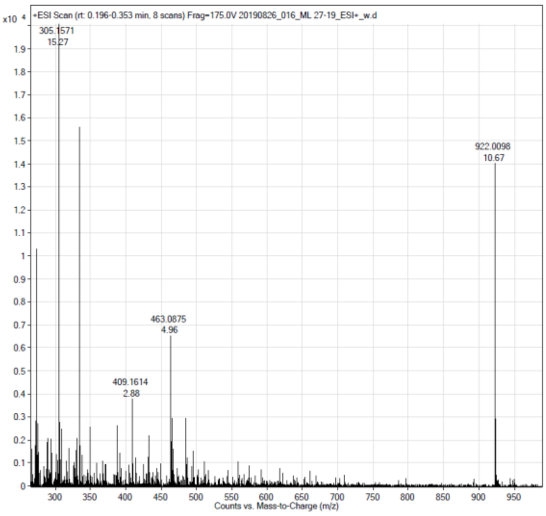


Figure S49. HRMS spectrum of compound **4c**

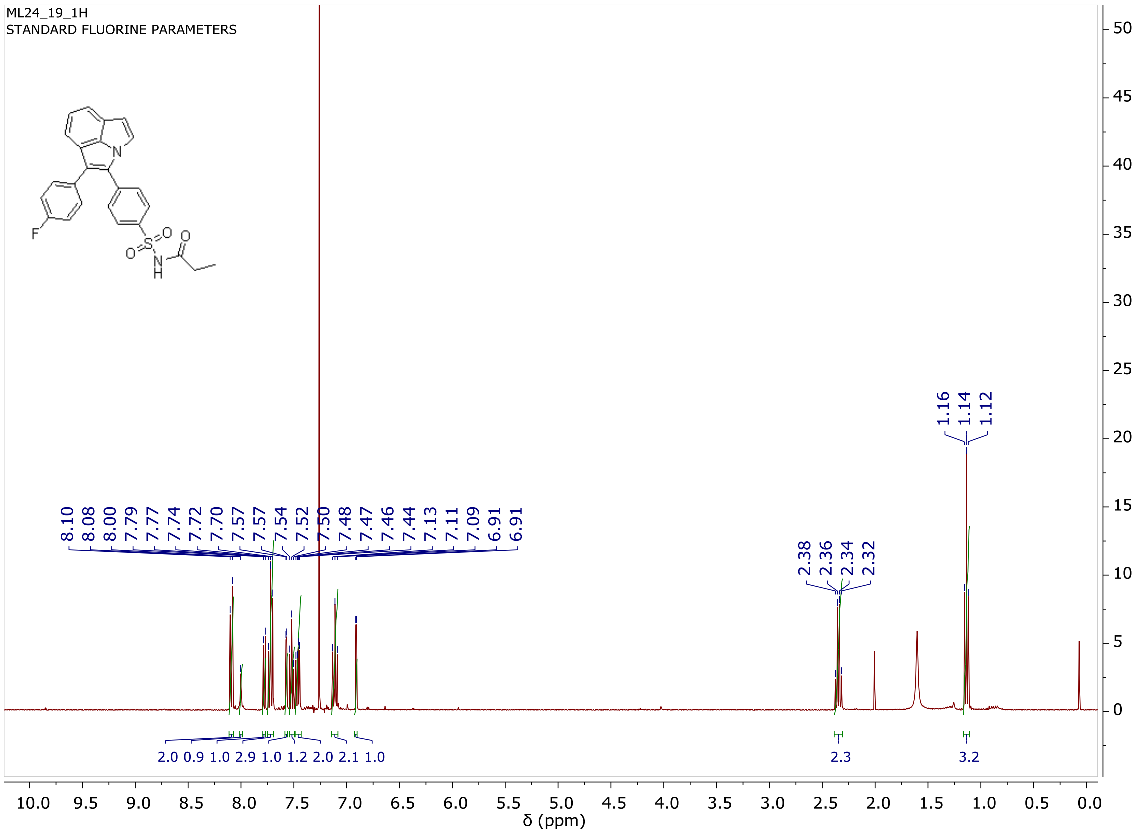


Figure S50. $^{1}$H NMR spectrum of compound **4d** in $CDCl_3$

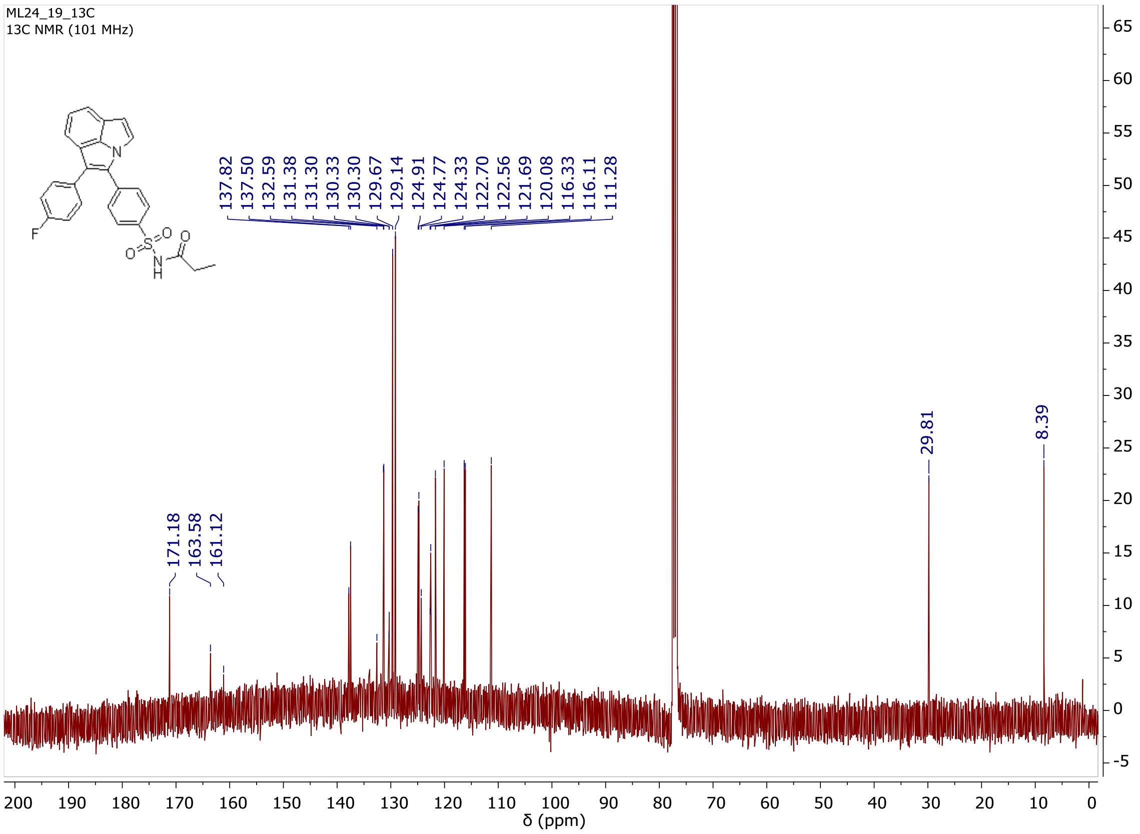


Figure S51. $^{13}$C NMR spectrum of compound **4d** in $CDCl_3$

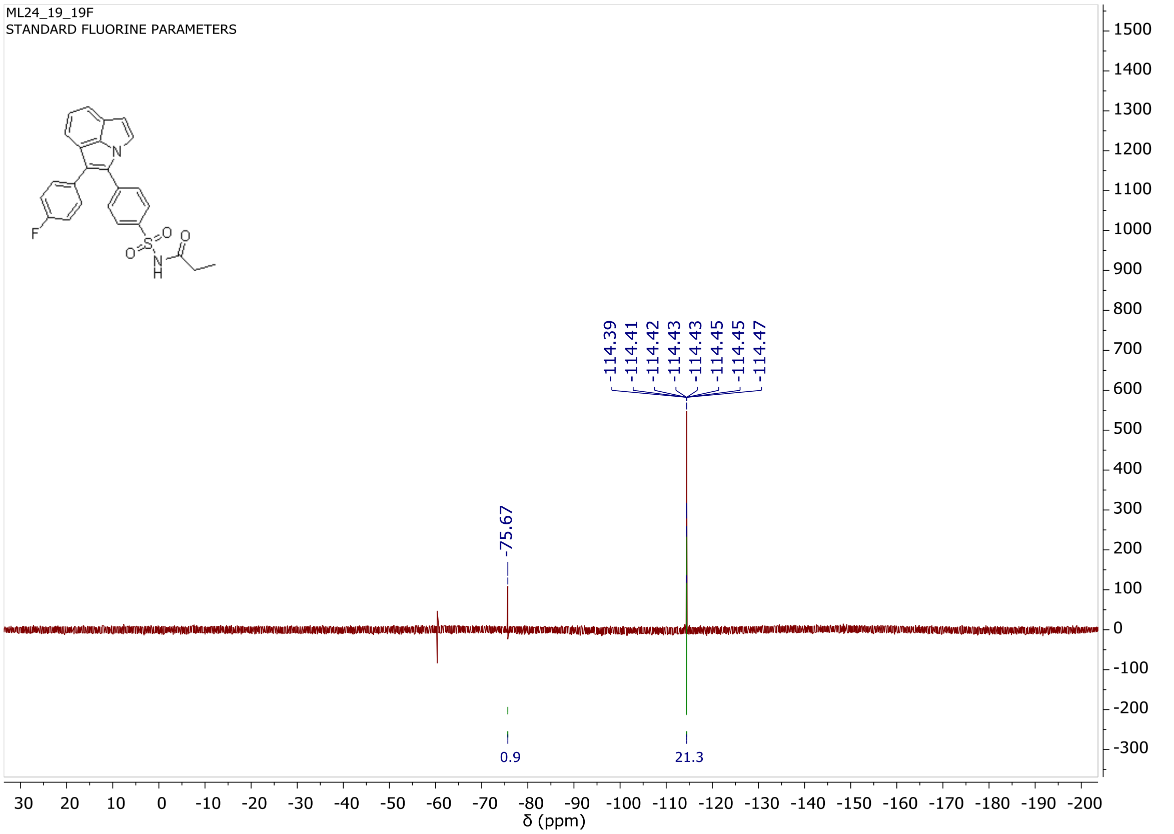


Figure S52. $^{19}$F NMR spectrum of compound **4d** in $CDCl_3$

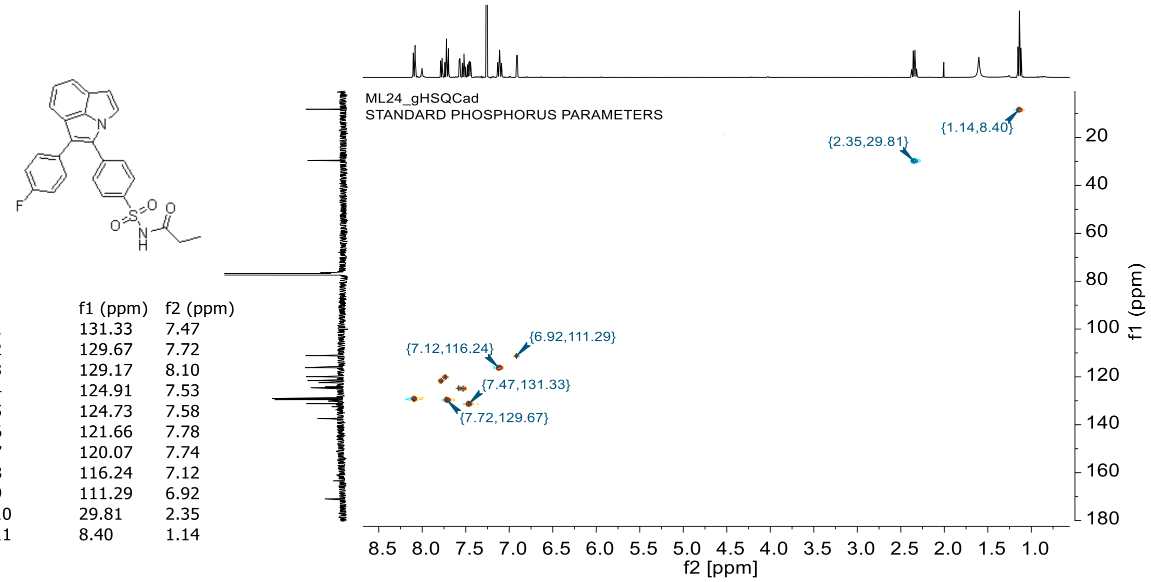


| | f1 (ppm) | f2 (ppm) |
|---|---|---|
| 1 | 131.33 | 7.47 |
| 2 | 129.67 | 7.72 |
| 3 | 129.17 | 8.10 |
| 4 | 124.91 | 7.53 |
| 5 | 124.73 | 7.58 |
| 6 | 121.66 | 7.78 |
| 7 | 120.07 | 7.74 |
| 8 | 116.24 | 7.12 |
| 9 | 111.29 | 6.92 |
| 10 | 29.81 | 2.35 |
| 11 | 8.40 | 1.14 |

Figure S53. HSQC spectrum of compound **4d** in $CDCl_3$

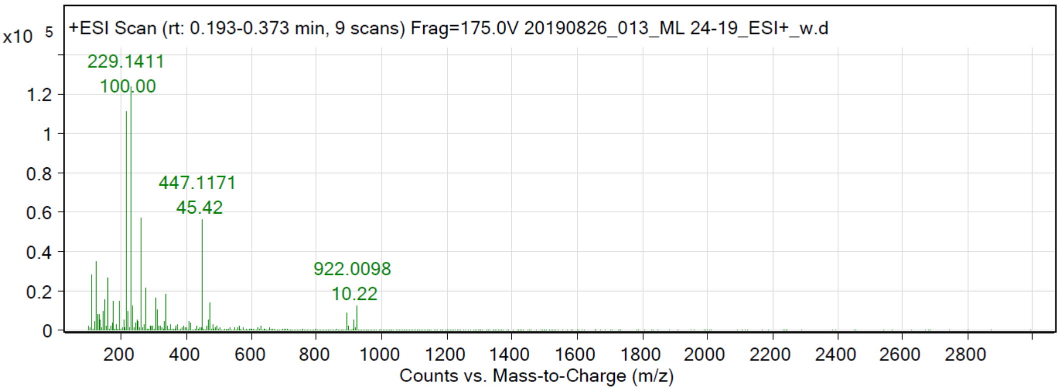


**Peak List**

| *m/z* | z | Abund |
|---|---|---|
| 106.0043 | | 27947.44 |
| 121.0509 | 1 | 33566.79 |
| 123.0916 | | 35098.75 |
| 158.9639 | | 27044.28 |
| 217.1047 | 1 | 111031.84 |
| 229.1411 | 1 | 123962.66 |
| 261.131 | 1 | 57119.01 |
| 273.1674 | 1 | 21577.87 |
| 335.0043 | | 18749.91 |
| 447.1171 | 1 | 56299.96 |

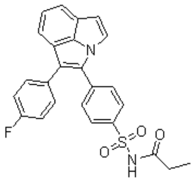

Figure S54. HRMS spectrum of compound **4d**

## Optimization of *N*-propionamide formation

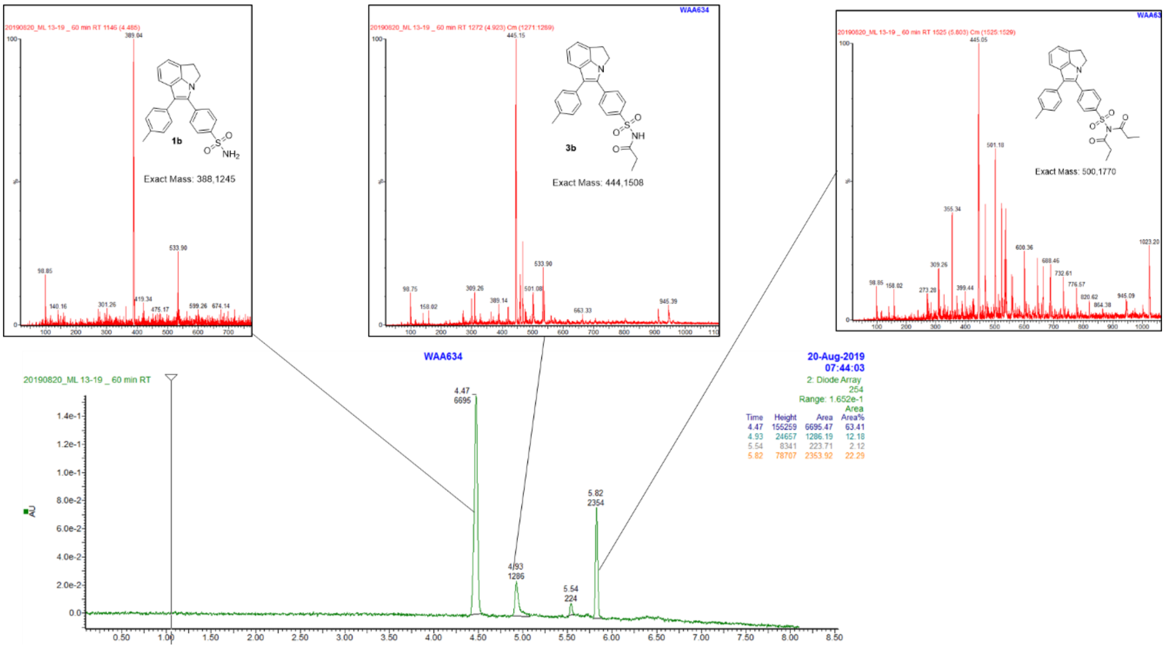


Figure S55. Exemplary UPLC-MS chromatogram resulting from the synthesis of **3b** using triethylamine as base.

The following procedure was used: The (dihydro)pyrrolo[3,2,1-*hi*]indole **1b** (1.39 mg, 3.58 µmol, 1.0 equiv) and triethylamine (0.65 µL, 4.71 µmol, 1.32 equiv) were dissolved in anhydrous DCM (108 µL). Then, propionyl chloride (0.82 µL, 0.87 mg, 9.38 µmol, 2.62 equiv) was added and the solution was stirred at room temperature for 60 min. The analytical HPLC sample was taken, the solvent was removed and the sample redissolved in acetonitrile for further analysis by UPLC-MS.

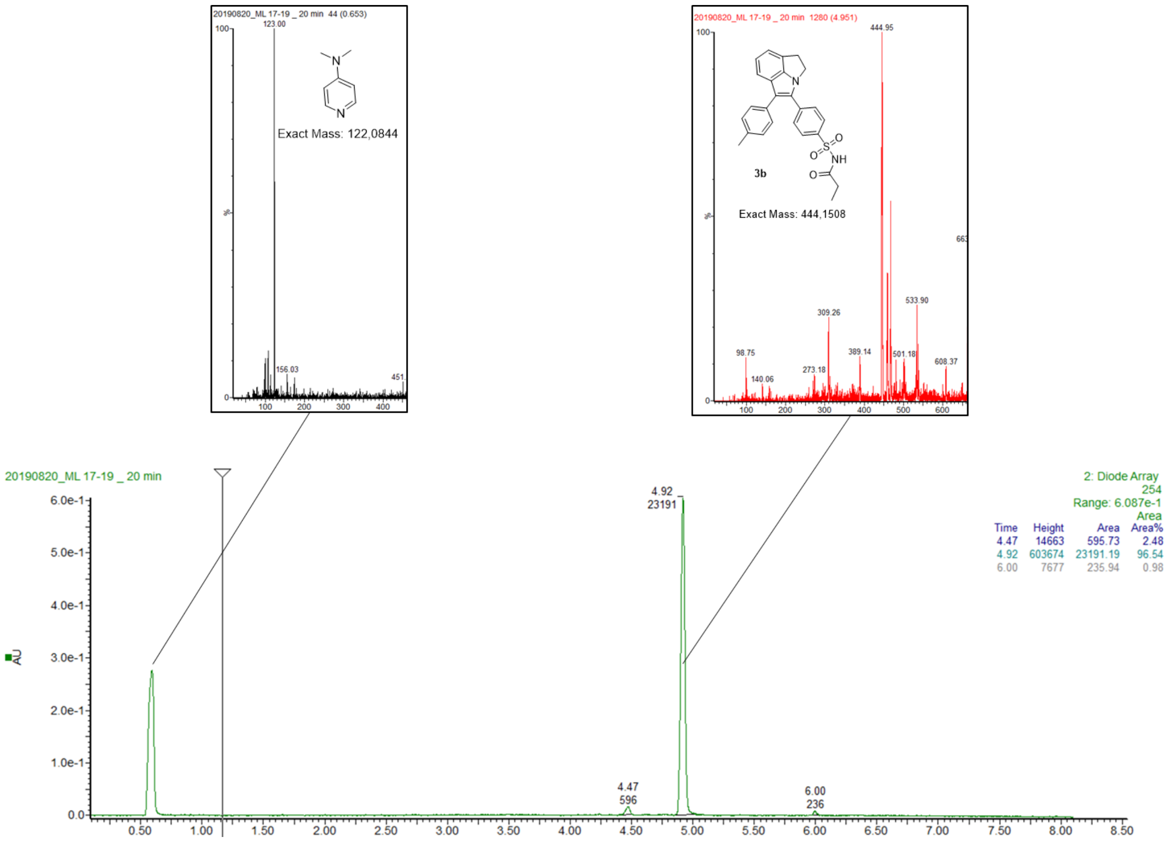


Figure S56. Exemplary UPLC-MS chromatogram resulting from the synthesis of **3b** using 4-dimethylaminopyridine as base.

The following procedure was used: The (dihydro)pyrrolo[3,2,1-*hi*]indole **1b** (1.76 mg, 4.53 µmol, 1.0 equiv) and dimethylaminopyridine (1.38 µL, 11.33 µmol, 2.50 equiv) were dissolved in anhydrous THF (137 µL) and DCM (137 µL). Then, propionyl chloride (0.46 µL, 0.49 mg, 5.94 µmol, 1.31 equiv) was added and the solution was stirred at room temperature for 20 min. The analytical HPLC sample was taken, the solvent was removed and the sample redissolved in acetonitrile for further analysis by UPLC-MS.

© 2019 by the authors. Submitted for possible open access publication under the terms and conditions of the Creative Commons Attribution (CC BY) license (http://creativecommons.org/licenses/by/4.0/).
